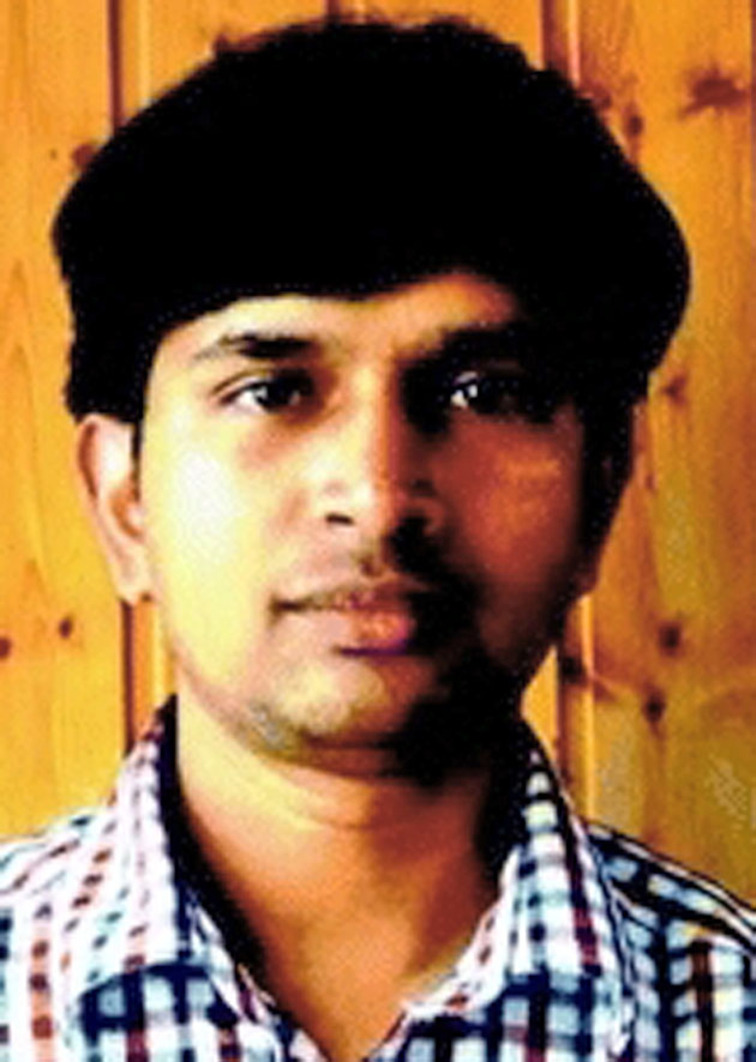# Recent Advances in the Domain of Cyclic (Alkyl)(Amino) Carbenes

**DOI:** 10.1002/asia.202101301

**Published:** 2022-02-26

**Authors:** Saroj Kumar Kushvaha, Ankush Mishra, Herbert W. Roesky, Kartik Chandra Mondal

**Affiliations:** ^1^ Department of Chemistry Indian Institute of Technology Madras Chennai 600036 India; ^2^ Institute of Inorganic Chemistry Tammannstrasse 4 D-37077 Göttingen Germany

**Keywords:** Catalysis, Cyclic (alkyl)(amino) carbenes, Luminescence, Radical chemistry, Carbene metal amides

## Abstract

Isolation of cyclic (alkyl) amino carbenes (cAACs) in 2005 has been a major achievement in the field of stable carbenes due to their better electronic properties. cAACs and bicyclic(alkyl)(amino)carbene (BicAAC) in essence are the most electrophilic as well as nucleophilic carbenes are known till date. Due to their excellent electronic properties in terms of nucleophilic and electrophilic character, cAACs have been utilized in different areas of chemistry, including stabilization of low valent main group and transition metal species, activation of small molecules, and catalysis. The applications of cAACs in catalysis have opened up new avenues of research in the field of cAAC chemistry. This review summarizes the major results of cAAC chemistry published until August 2021.

## Graphical abstract

**The present review** highlights recent developments in the domain of cyclic (alkyl) (amino) carbenes with the emphasis on their applications in the field of catalysis, activation of small molecules, radical chemistry, organic light‐emitting diodes (OLEDs), medicinal chemistry, and single‐molecule magnets (SMMs).

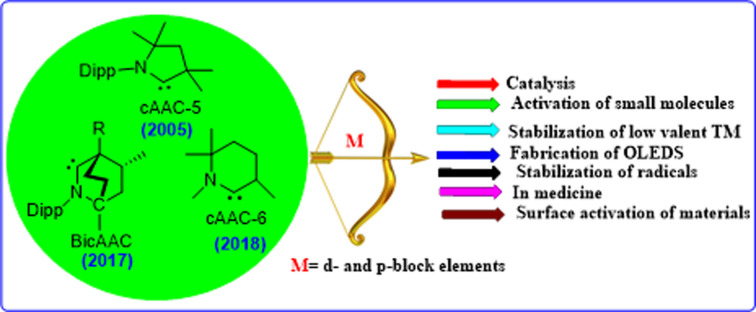

## 1 Introduction

Carbenes are neutral compounds containing a divalent carbon atom with six valence electrons which act as reaction intermediates in various chemical processes. These intermediates have been considered transient species for a long time. As carbenes have no electron octet and are coordinatively unsaturated, therefore, they act as highly reactive species. The works by Öfele^[1]^ and Wanzlick^[2]^ in 1968 has introduced simple routes for carbenes chemistry, which later on led to the isolation of stable carbenes.^[3]^ Despite many attempts to isolate uncoordinated stable carbene for decades, it remained elusive until pioneering works in the 1980s and early 1990s.^[4]^ However, in 1988 Bertrand et al.^[5]^ in a seminal work reported the preparation of [bis(diisopropylamino)phosphino](trimethylsilyl) carbene (**1**), the first isolable carbene as a liquid stabilized by favorable interactions with adjacent phosphorus and silicon substituent. The unusual stability of this carbene is due to electron transfer from the hetero‐atom to the empty π‐orbital of the carbene center.^[5]^ This discovery prompted many researchers to try different substituents for the synthesis of stable carbenes. In 1991, Arduengo^[6]^ synthesized crystalline storable imidazol‐2‐ylidene (**2**), a stable carbene, in a moisture and oxygen‐free environment, which became the first representative of such a class of stable carbenes called N‐heterocyclic carbenes (NHCs). Since then several NHCs have been reported and extensively studied for their applications especially in the catalytic transformations that have opened up new avenues of research. The pioneering work by Herrmann^[7]^ established NHCs as excellent ligands for transition metal‐based catalysis.^[8]^ The silver complexes of NHCs have even found applications in medicine as antitumor agents.^[9]^

**Figure 1:**
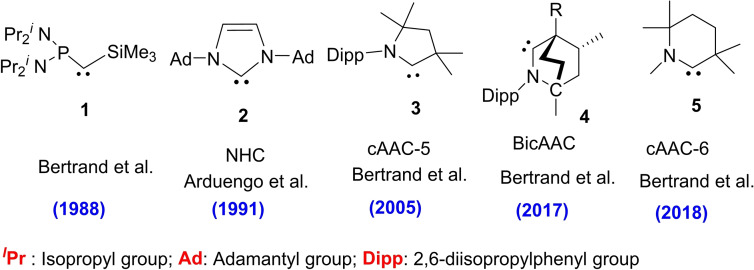
First reported members of NHC, cAAC‐5, BicAAC, and cAAC‐6.

In 2005, Bertrand's research group reported the preparation of a new class of stable five‐membered carbenes called cyclic(alkyl)(amino)carbenes (cAAC‐5) (**3**)^[10]^ (or in general cAACs), which possess better electrophilic and nucleophilic properties compared to NHCs. In May 2017, Bertrand et al. reported the synthesis of bicyclic(alkyl)(amino)carbene (BicAAC) (**4**)^[11]^ by modifying the cAAC skeleton. BicAACs have geometry similar to NHCs and are claimed to display enhanced nucleophilicity and electrophilicity compared to cAACs and NHCs.^[11]^ Surprisingly, in June 2018, after one year of synthesis of BicAACs, Bertrand's research group^[12]^ reported another striking six‐membered cAACs (cAAC‐6) (**5**) which has the least HOMO‐LUMO gap among all known stable carbenes (Figure 1).

Therefore, cAACs (cAAC‐5, BicAAC, and cAAC‐6) are better than NHCs in stabilizing paramagnetic species^[13]^ and activating small molecules and enthalpically strong bonds.^[14]^ Nowadays, cAACs have emerged as the most powerful tools in different branches of chemistry and are being utilized in catalysis, medicine, and material science.^[15]^ The comparative reactivity of different stable carbenes has been performed and cAACs have emerged as important ligands.^[16]^ Consequently, cAACs have been employed to stabilize paramagnetic complexes with metals in lower or even zero oxidation states.^[17]^ The cAAC chemistry of results published till 2017 has been reviewed[^18^, ^19^] elsewhere in detail; therefore, a generalized view of cAAC chemistry having details of recently published literature will be covered.

## 2 Comparison Between NHCs and cAACs

**Figure 2:**
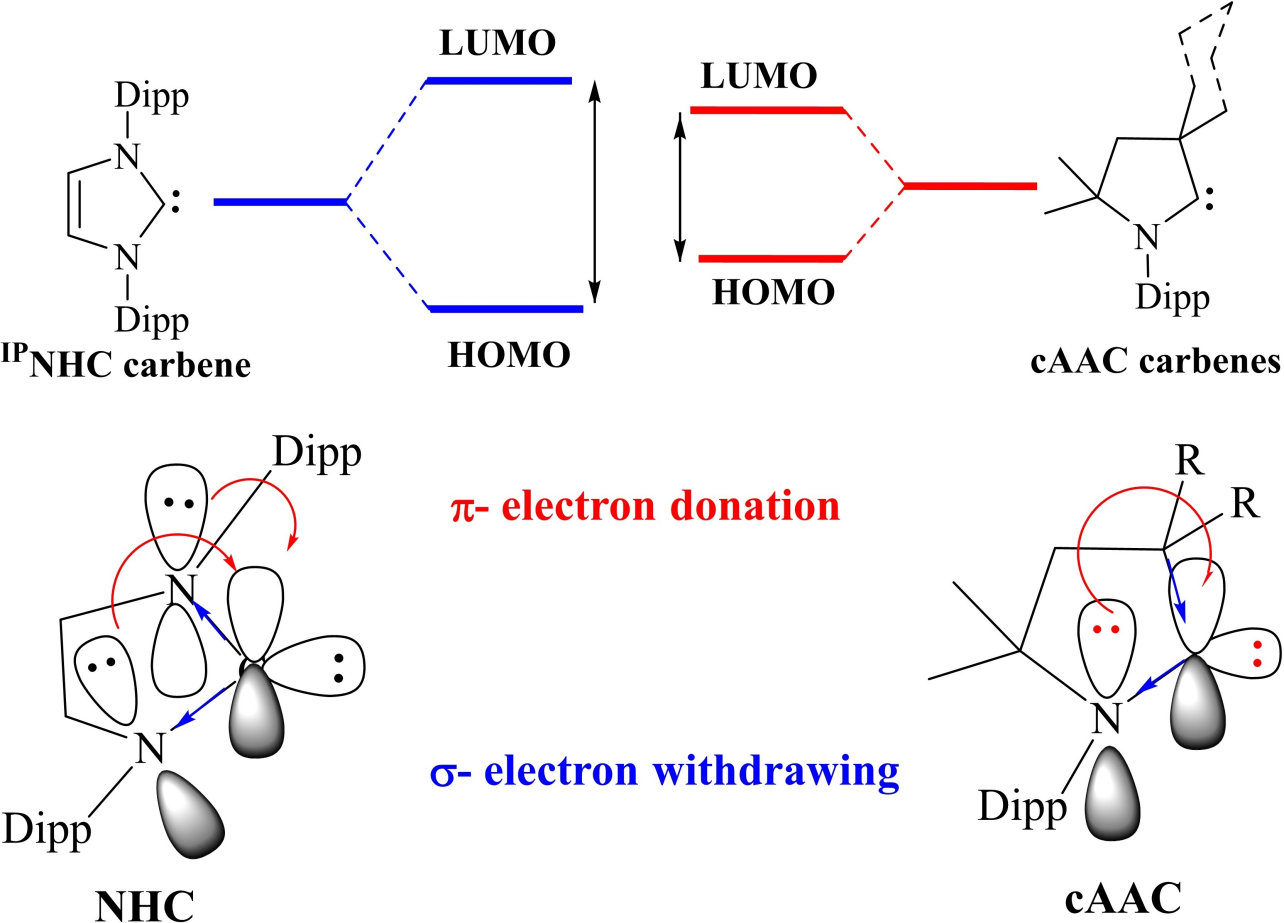
Schematic comparison between NHC and cAAC carbenes. Reproduced with permission from Ref. [23]. Copyright (2015) The Royal Society of Chemistry.

**Figure 3:**
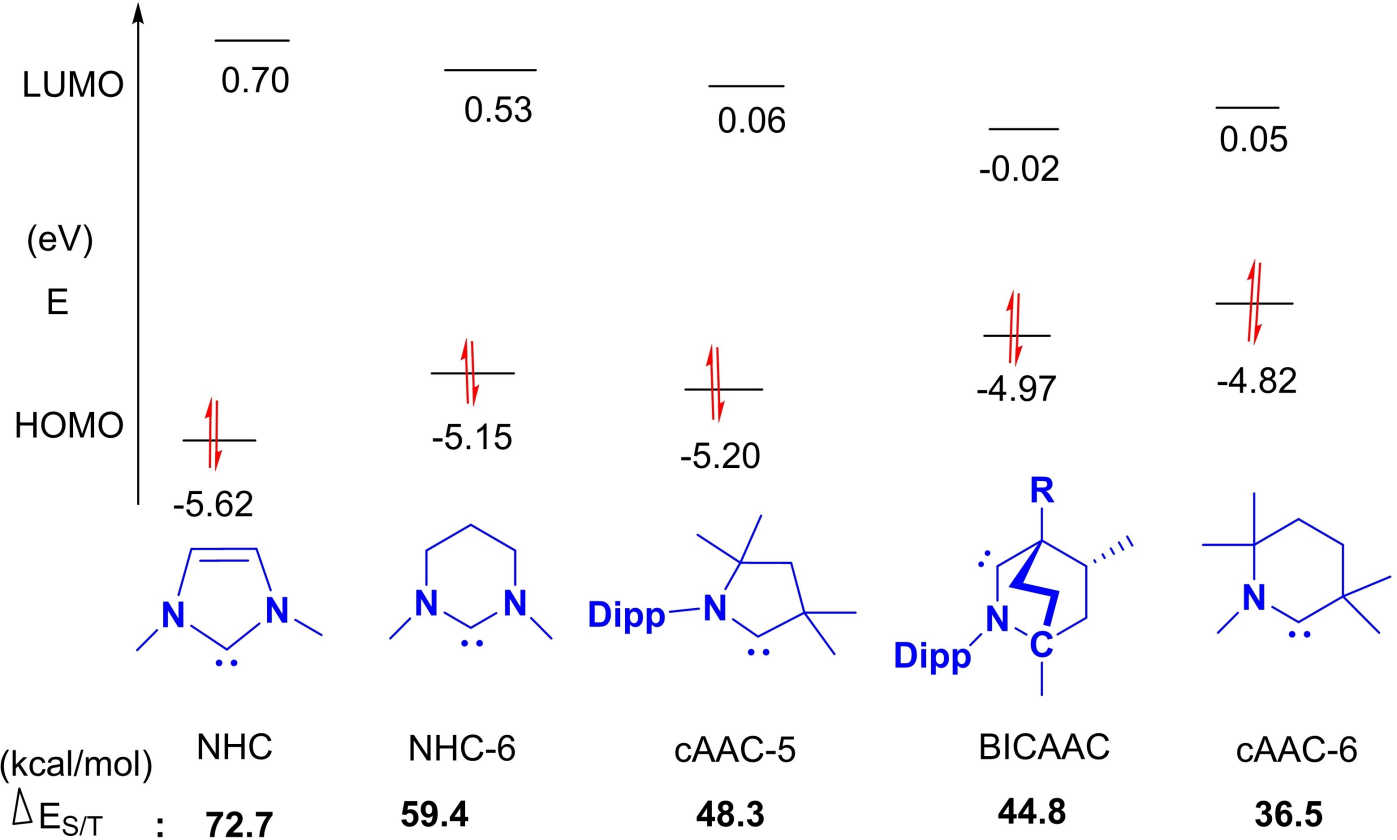
HOMO‐LUMO gap (eV) and ΔE_**S/T**_ (kcal/mol) of NHC, NHC‐6, cAAC‐5, BicAAC, and cAAC‐6 (Calculated at the B3LYP/def2‐TZVPP level of theory). Reproduced with permission from Ref. [12]. Copyright (2018) American Chemical Society.

NHCs are defined as heterocyclic species containing a carbene carbon and at least one nitrogen atom within the ring structure.^[20]^ The substitution of a π‐donating amino group in NHCs by σ‐donating alkyl group gives cAAC carbenes. This replacement leads to the lower‐ lying LUMO that in fact reduces the HOMO‐LUMO energy gap,^[21]^ which makes the carbon center of cAACs more electrophilic (π‐accepting) as well as more nucleophilic (σ‐donating) than the corresponding carbon atom of NHCs.^[15]^ Computational studies reveal that the HOMO‐LUMO and the singlet‐triplet energy gaps (ΔE_S/T_) in cAACs are slightly smaller than those of NHCs.[^14^, ^22^] Recently reported BicAACs and six‐membered cyclic(alkyl)(amino)carbenes (cAAC‐6)^[12]^ have even smaller HOMO‐LUMO and singlet‐triplet energy (ΔE_S/T_) gaps when compared with those of five‐membered cAACs (cAAC‐5) and NHCs. Therefore, it is obvious that different types of cyclic(alkyl)(amino)carbenes (cAAC‐5, BicAACs, and cAAC‐6) reported till to date are exhibiting better electronic ligand properties that make them better ligands when compared with NHCs (Figures 2 and 3).^[12]^

**Figure 4:**
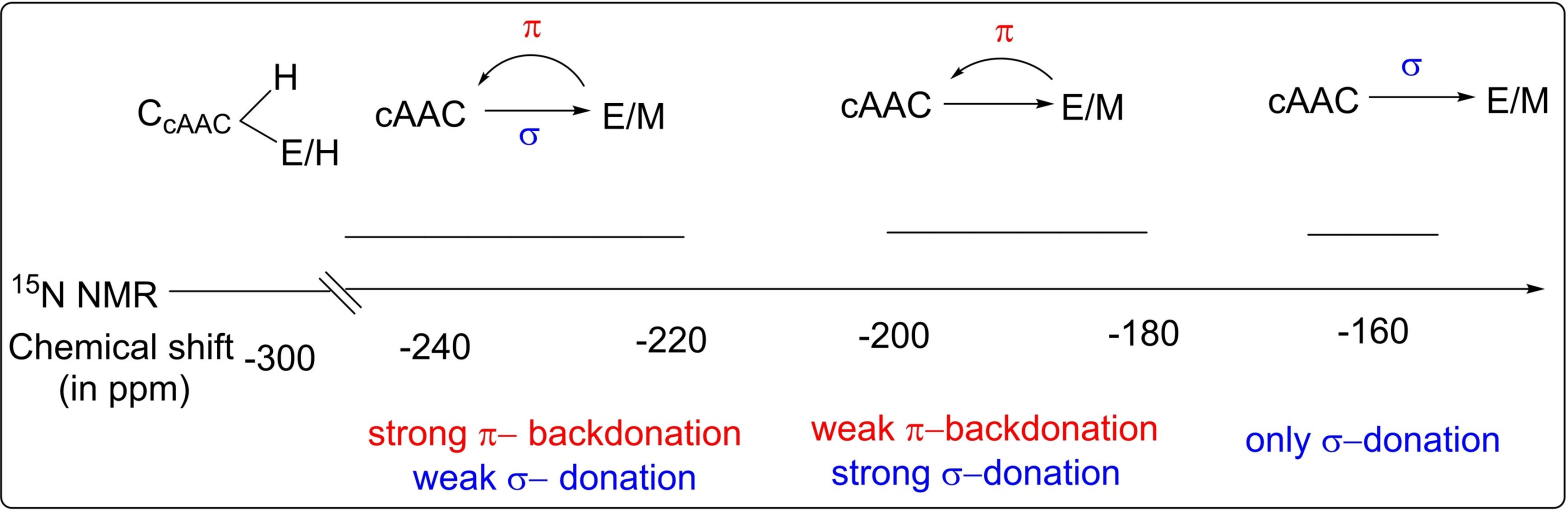
^15^N NMR scale for the prediction of the extent of σ/π‐donation for E/M (^15^N NMR chemical shift values, in ppm). Reproduced with permission from Ref. [15]. Copyright (2017) Viley‐VCH Verlag GmbH & Co. KGaA Weinheim.

Better donating properties of cAACs have also been experimentally confirmed using Tolman electronic parameters (TEP)^[24]^ that are based on complexes of cAACs and NHCs with model compounds‐Rh(CO)_2_Cl and Ir(CO)_2_Cl. TEP was originally developed for phosphine complexes in which the electron‐donating ability of ligands is evaluated by measuring infra‐red stretching frequencies of carbonyl ligands. However, it is found that the donation ability of cAACs is only slightly better than that of NHCs by studying the IR stretching frequencies of bonded CO ligands.^[25]^ Moreover, the TEP value is the measure of the overall donation ability of a ligand (σ‐donation minus π‐acidity). The π‐accepting properties of cAACs have also been measured by different methods such as by using ^31^P NMR chemical shift of phenylphosphinidene‐carbene adducts^[26]^ and ^77^Se NMR chemical shift of carbene‐selenium adducts.[^27^, ^28^] It is important to note that the latter method is advantageous owing to the broader ^77^Se NMR scale (Δδ >850 ppm). However, ^77^Se is toxic and ^77^Se NMR is not easily available like ^31^P NMR.^[15]^ We have been able to utilize the HMBC ^15^N NMR scale to predict the extent of π‐back bonding of elements (main group E and metal M) to the carbene center (E/M→cAAC) as well as σ‐donation from the carbene center to E/M (cAAC→E/M).^[29]^ In order to evaluate donation properties, a series of cAAC−E/M adducts were studied and ^15^N NMR chemical shift values were assigned (Figure 4). The chemical shift value for ^15^N nuclei is in the range of −130 ppm to −315 ppm and it predicts whether σ‐donation of carbene or π‐donation of E/M is stronger. When the chemical shift value is obtained in the range of −170 ppm to −200 ppm then σ‐donation (cAAC→E/M) of cAAC is stronger and when it is observed below −220 ppm then π‐backdonation is stronger than σ‐donation.^[29]^ This scale, in contrast to other methods, uses the E/M variant instead of carbene^[30]^ Therefore, it cannot be used to compare donation properties of different classes of free carbenes. However, it can be used to evaluate the properties of E/M.

## 3 Areas of cAAC Chemistry

Since the discovery of cAACs in 2005, the chemistry of this class of ligands has been explored in different directions. The important domains of cAAC chemistry are discussed in the following sub‐headings.

### 3.1 cAAC‐Stabilized Metal Complexes in Organic Catalysis

**Scheme 1:**
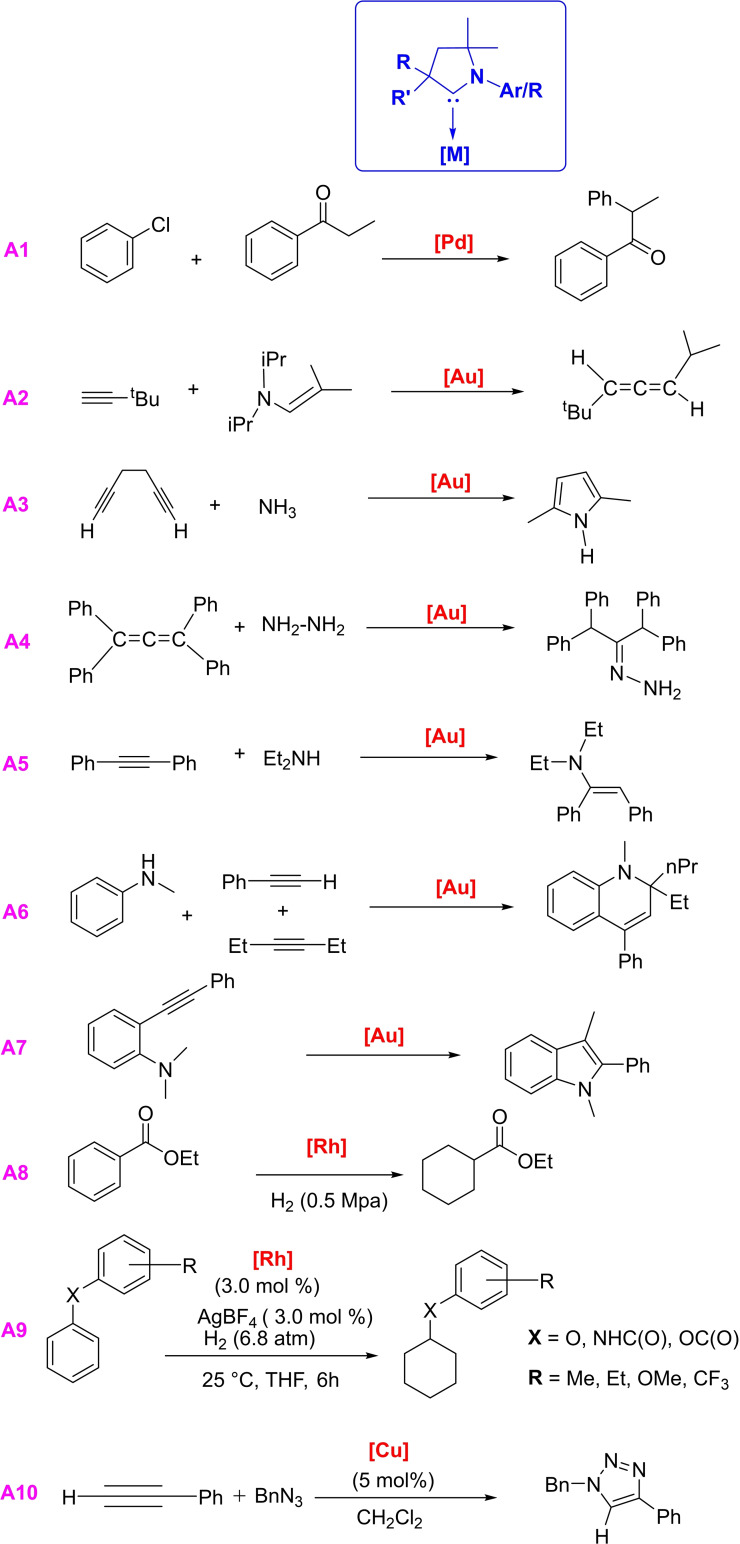
Reactions catalysed by cAACs supported metal complexes.

Transition metal ions/atoms stabilized by stable singlet carbenes have played a vital role in different fields of organometallic and organic chemistry, especially towards the catalytic organic transformations. NHCs based complexes have been widely utilized in transition metal catalysis in the recent past, but the catalytic applications of cAAC complexes are not that much explored.[^8^, ^31^] However, better electronic properties and steric nature of cAACs have been used to improve established chemical transformations and some new processes catalyzed by cAACs complexes have been published. In 2005, Bertrand et al. reported that (cAAC)PdCl(allyl) complexes catalyze the α‐arylation of propiophenone with aryl chloride (**A1**) (Scheme 1). Moreover, this reaction can be carried out under much milder conditions than with phosphine and NHCs containing complexes, and the turnover number (TON) is attained up to 7000.^[10]^ It has also been demonstrated that higher steric bulk in cAACs, than in NHCs and phosphine, also increases the catalytic activity of cAACs complexes, owing to sp^3^‐hybridization of the α‐carbon.^[15]^ According to the report by Grunwald et al., cAAC stabilized palladium complexes can undergo oxidative addition with water, alcohols, and amines and can be a useful catalyst for O−H and N−H bond activation.^[32]^ Gold(I)‐complexes bearing cAACs as ligands are probably the most thoroughly studied complexes with a broad range of applications in catalysis. In 2007, Bertrand's group demonstrated that cationic cAACs−Au (I) complexes can promote the coupling of terminal alkynes with enamines to produce allenes,^[33]^ while with other catalysts this coupling gives propargyl amines (**A2**). Further, these cAACs−Au(I) complexes can also catalyze hydroamination of unactivated alkynes and allenes with ammonia^[34]^ (**A3**) and parent hydrazine (**A4**)^[35]^ which is otherwise challenging to accomplish. Interestingly, inter‐ and intramolecular hydroamination of allenes and alkynes with secondary amines is also catalyzed by cAACs−Au (I) complexes (**A5**).[^36^, ^37^] Gold(I)‐complexes can also promote the hydroamoniumation and methylamination of alkynes (**A6**)^[38]^ as well as single pot synthesis of 1,2‐hydroquinoline (**A7**).^[39]^ Recently cAACs−Cu(I) complexes^[40]^ and cAACs−Au(I) complexes^[41]^ have been reported to catalyze hydrohydrazination of alkynes and allenes with dimethylhydrazine. The pioneering work by Zeng's research group demostrates the utility of Rh(cAAC) complexes in selective hydrogenation of aromatic ketones and phenols(**A8**).^[42]^ Similarly, Glorius et al. also showed hydrogenation of fluoroarenes by Rh(cAAC) complexes.[^43^, ^44^] Bullock et al. reported^[45]^ Rh(cAAC) catalyzed arene hydrogenation (**A9**) of ethers, amides, and esters at room temperature and low hydrogen pressure. In this reaction, cAAC‐stabilized rhodium complex forms Rh‐nanoparticles, which carry out site‐selective electrocatalysis. Bertrand et al. have shown the hydrolytic dehydrogenation of ammonia borane by a first stable copper borohydride complex [(cAAC)CuBH_4_].^[46]^ In this catalytic reaction, the amount of hydrogen gas produced reaches 2.8 H_2_/BH_3_NH_3_ with a turnover frequency of 8400 mol_H2_ mol_cat_^−1^ h^−1^ at 25 °C. Additionally, Bertrand et al. have also shown copper‐catalyzed azide‐alkyne cycloaddition (**A10**).^[47]^ Recent studies have shown that copper and gold complexes with imine functionality in the side chain of cAAC ligands exhibit high catalytic activity for hydroarylation reactions.^[48]^ It is apparent from the above discussion that cAAC‐gold complexes are the most common catalysts for different reactions. However, other cAAC‐coinage metal complexes are not so common in catalytic applications. A few of the cAAC‐copper halide complexes have been reported which show catalytic properties. Recently, Whittelsey et al. reported a cAAC−Cu(I)F complex which catalyzes aldehyde allylation, though the yield of the reaction is low.^[49]^

**Scheme 2:**
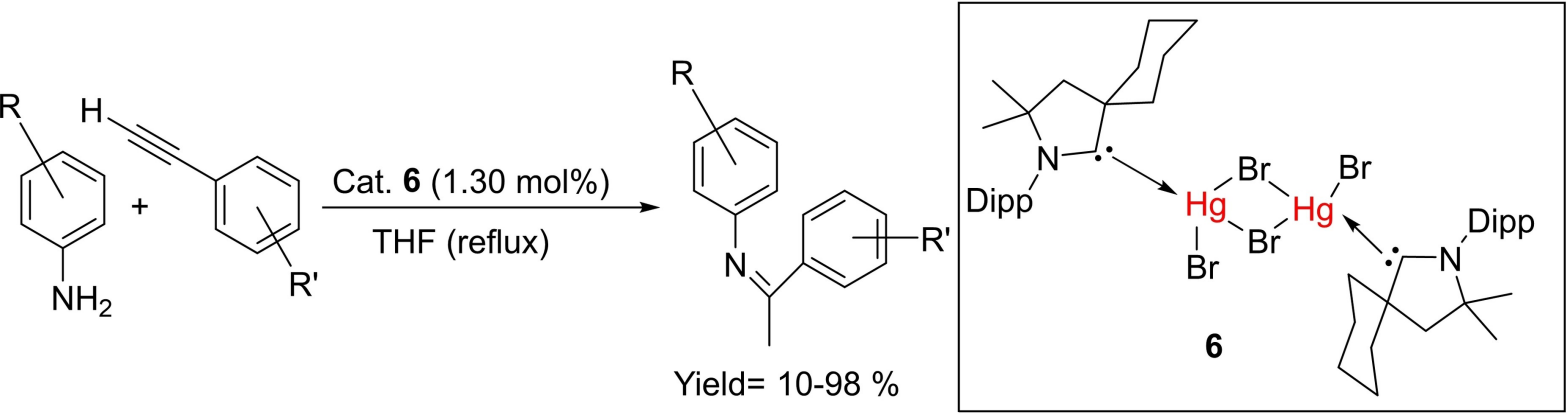
Hydroamination reaction between the derivatives of phenylacetylene and aniline catalyzed by cAAC−Hg(II) complex (**6**).

**Figure 5:**
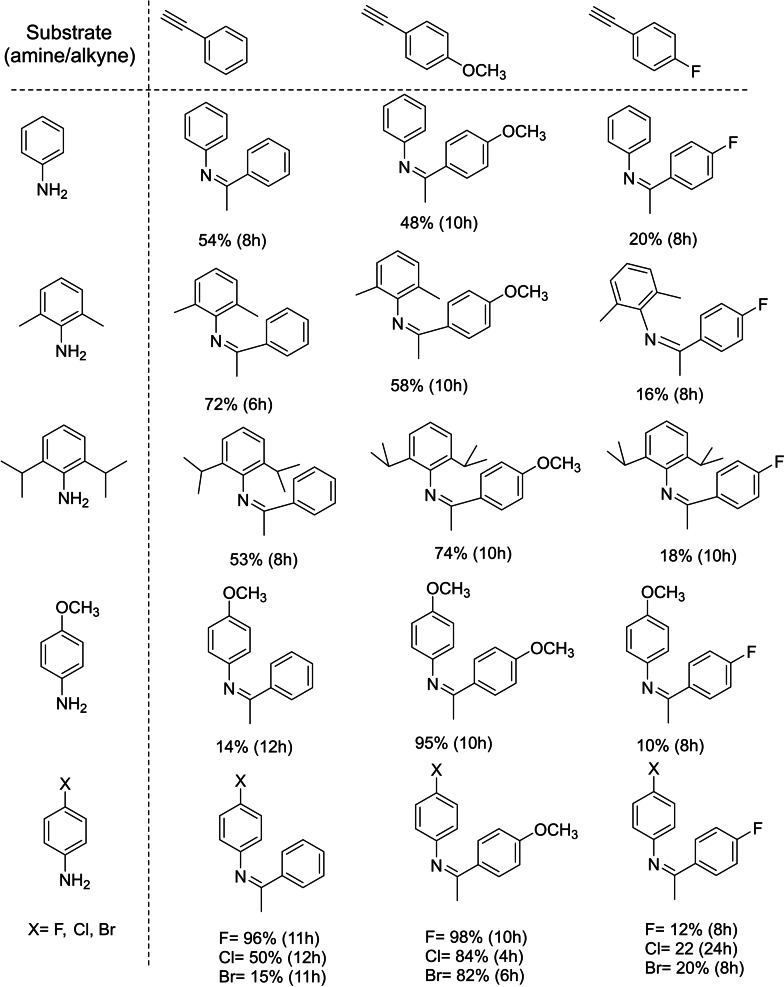
Substrate scope of intermolecular hydroamination catalysed by **6** and percentage product yields.

It is interesting to note that just like cAACs−Au(I) complexes, cAACs supported Hg(II)‐complexes also catalyze intermolecular hydroamination reactions of alkynes. The very first hydroamination reaction of alkynes catalyzed by cAAC−Hg(II) complex was reported by Singh et al. in 2018.^[50]^ In this catalytic reaction, intermolecular hydroamination of phenylacetylene with aniline in the presence of cAAC−Hg(II) complex (**6**) was demonstrated (Scheme 2).^[50]^ The reaction followed Markownikoff regioselectivity and 10–98% product yields were recorded. The catalyst has a wide substrate scope in catalyzing hydroamination with several derivatives of phenylacetylene and aniline (Figure 5).

**Scheme 3:**
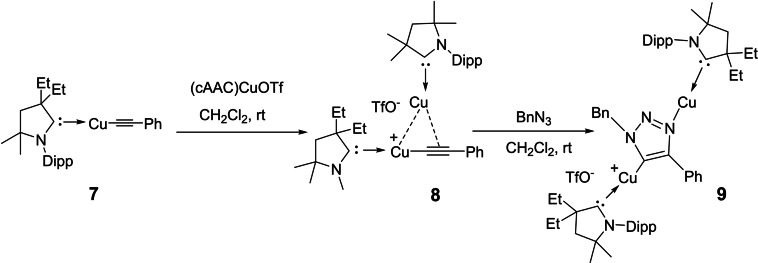
Isolation of catalytically active species (**8** and **9**) (Bn: Benzyl group).

Recently, studies have shown that cAACs can be used in the isolation of catalytically active metal species, which were earlier considered to be short‐lived intermediates. In a first such investigation, Fokin et al. studied copper‐catalyzed 1, 3‐dipolar addition of azides to terminal alkynes.^[51]^ In this reaction, the mononuclear complex (**7**) was found to be a less active catalyst than the binuclear complexes (**8**), and (**9**) (Scheme 3). However (**8**) has been described as a highly reactive intermediate that cannot be isolated.^[52]^ Using cAACs, Bertrand's group was able to isolate σ,π‐bis(copper)acetylene (**8**) and bis(metalated) triazole complex (**9)**.^[53]^ Ma et al. demonstrated catalytic properties of cAAC stabilized copper(I) complex in Friedel‐Craft reaction of N,N‐dialkylanilines with styrenes.^[54]^

**Scheme 4:**
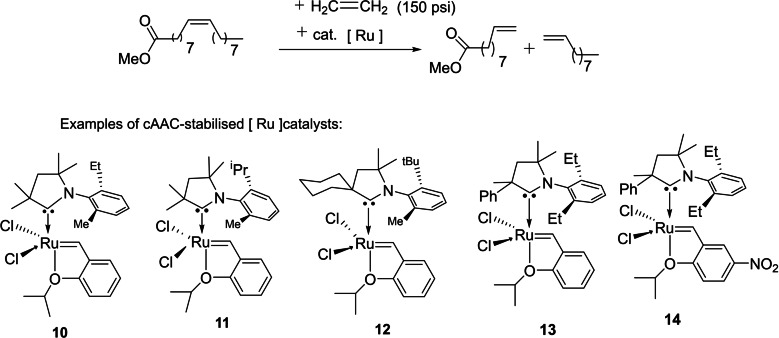
Ethenolysis of methyl oleate and cAAC‐ligated ruthenium catalysts.

Although NHC‐ligated ruthenium complexes are being used in the catalytic olefin metathesis and industry has also shown interest in using this catalyst for the manufacturing of pharmaceutical ingredients and special chemicals.^[55]^ However, the low efficiency of NHC‐based ruthenium catalysts does not allow the synthesis of some of these industrially important classes of compounds. To address this limitation, researchers were prompted to explore cAAC‐based ruthenium catalyst. Therefore, olefin metathesis is becoming one of the intensively investigated inorganic processes catalyzed by cAAC‐based ruthenium complexes. In 2007, Bertrand and Grubbs's research group collaboratively investigated the role of cAACs as ligands in ruthenium‐catalyzed olefin metathesis.[^56^, ^57^] The major success in the transition metal catalysis was realized by exchanging one phosphine ligand of first‐generation Grubbs or Hoveyda‐Grubbs catalyst with the more σ ‐donating NHCs.^[58]^ Subsequently, air‐stable precatalyst **10, 11**, and **12** were prepared by replacing PCy_3_ with cAACs ligand in the first Hoveyda‐Grubbs complexes and their successful utilization in several metathesis reactions revealed that **11** is the most active species (Scheme 4).[^59^, ^60^] Noteworthy is the point that the ethenolysis of methyl oleate was found to give the best result with TON values in the range of 200000–330000. Further studies have shown that cAAC‐bearing ruthenium catalysts **(11** and **12**) exhibit excellent efficiency in cross‐metathesis with ethylene resulting in the formation of a terminal C=C bond. Moreover, the performance of **11** and **12** were also tested for ring‐closing metathesis of diethyl malonate derivatives, and surprisingly, the best results comparable to those of NHC‐based catalysts were obtained. Therefore, they were found to be suitable candidates in the formation of internal olefins. Lately, Skowerski and co‐workers^[61]^ have demonstrated that compound **14** is capable of promoting challenging macrocyclization and cross‐metathesis with acrylonitrile reactions at very low loadings (10–20 ppm). Most recently, Grela et al. have also reported non‐glovebox ethenolysis of ethyl oleate and FAME at a larger scale utilizing a cAAC−Ru catalyst.^[62]^ The molecular modeling by computational calculations has also shown the interesting applications of cAAC based Hoveyda‐Grubbs catalyst in metathesis reactions.^[63]^ This is a remarkable achievement.

Moreover, recent developments, in the field of Ru−cAAC based catalysts have shown that this catalyst can also be used in the metathesis of renewable feedstocks such as phospholipids and vegetable oils in the protic media.^[64]^ This catalytic process gives yield up to 80% and can be carried out with catalyst loading as low as 0.05 mol % in the environmentally benign protic media. In addition to the cAAC‐5 based ruthenium complexes, cAAC‐6 ligands‐based ruthenium complexes have also been exploited for their metathesis activity.^[65]^

**Scheme 5:**
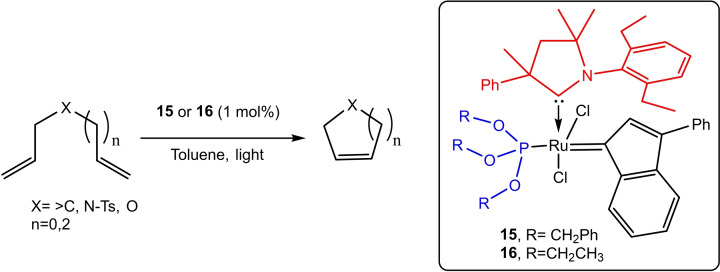
cAAC and phosphite ligand supported photoswitchable Ru metal complex catalyzed olefin metathesis.

Lemcoff et al. have synthesized photo‐switchable Ru catalyst by encasing superior electronic and steric properties of cAAC ligands with latency provided by phosphite ligands.^[66]^ The synthesized catalysts **15**, and **16** were tested for a series of olefin metathesis reactions and they gave excellent yield with high stereoselectivity (Scheme 5). The catalyst was recycled by exposing it to 405 nm light and it was again used for up to four cycles without loss of activity.

**Scheme 6:**
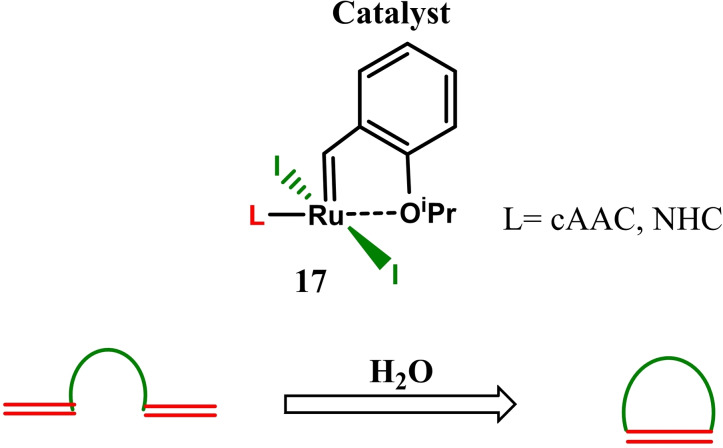
Effect of water on ring‐closing metathesis in presence of Ru catalyst.

Ruthenium catalysts are broadly used alkene metathesis and to perform this reaction high purity distilled solvents are required. In the reactions in which water is present as a co‐solvent, the rate of metathesis is degraded. In order to study the tolerance of water on ruthenium‐catalyzed olefin metathesis, Fogg et al. have investigated a series of Ru catalysts and reported that the presence of water triggers the catalyst decomposition; therefore, the yield of product is affected (Scheme 6). They found that the bulkier iodine and cAAC catalyst is the best choice.^[67]^ In continuation of their previous studies on iodine‐based Ru catalysts, Fogg et al. have synthesized second‐generation ruthenium‐diiodide catalysts (**17**) with cAAC and NHC ligands for the olefin metathesis in excellent yields.^[68]^

**Scheme 7:**
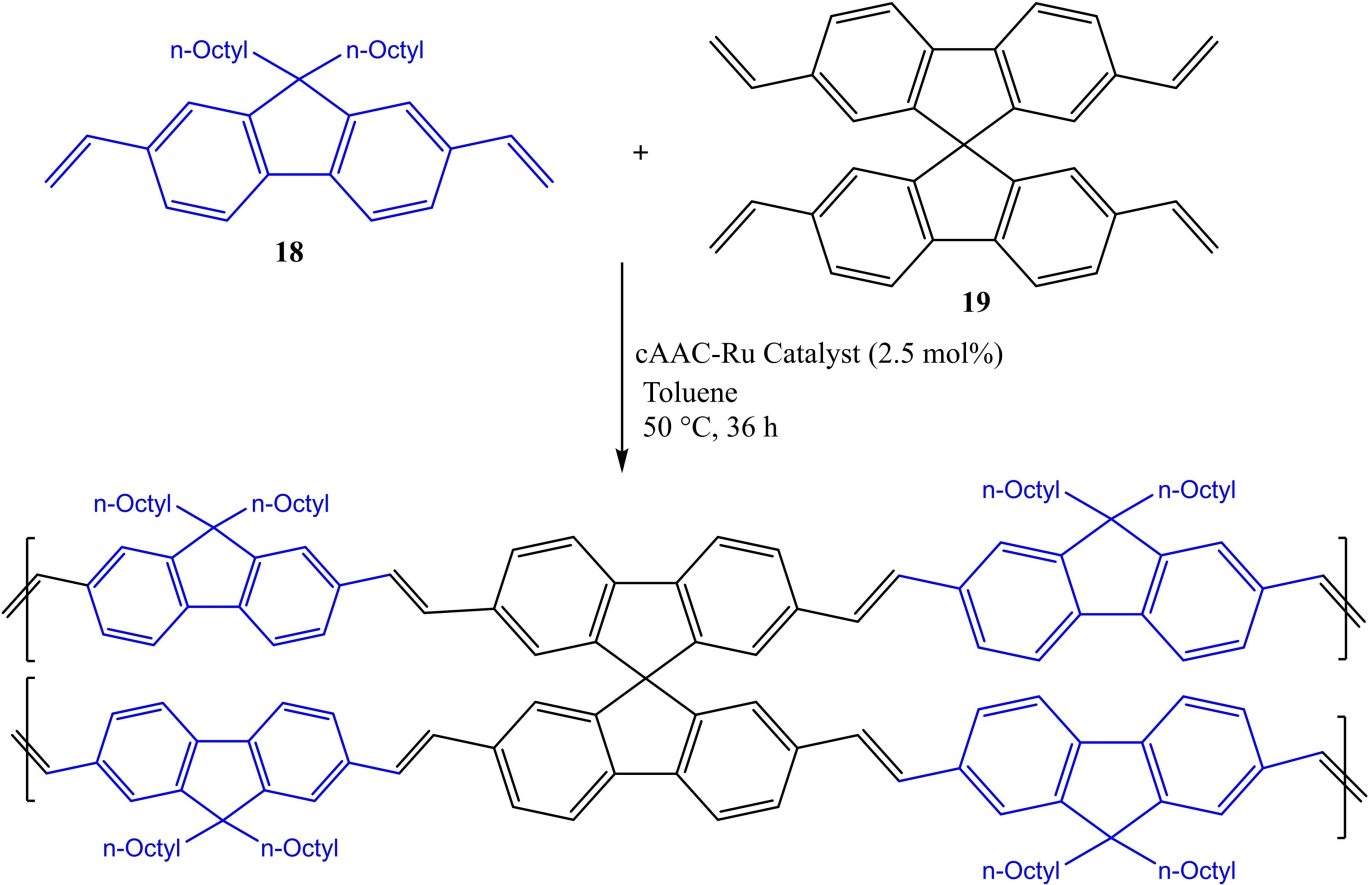
Synthesis of copolymerization product of 2,7‐divinyl‐9,9‐di‐noctylfluorene and 2,2’,7,7’‐tetravinyl‐9,9’‐spirobifluorene.

Hong et al. applied Grubbs‐type and Hoveyda‐type, cAAC stabilized ruthenium catalysts for olefin metathesis of 2,7‐divinyl‐9,9‐di‐noctylfluorene (**18**) and 2,2’,7,7’‐tetravinyl‐9,9’‐spirobifluorene (**19**) in different ratios to synthesize seven copolymerization products in good to excellent yields (Scheme 7).^[69]^ The copolymers with spirobifluorene units are better for polymer Light‐Emitting Diode (LED) devices and display improved performance like better turn‐on voltage, brightness, current, and power efficiency. Morvan et al. have summarized cAAC−Ru catalyzed olefin metathesis in a recent review.^[70]^

**Scheme 8:**
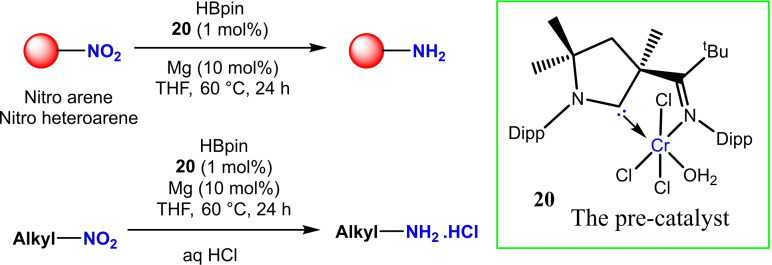
cAAC−Cr catalyzed conversion of NO_2_ in NH_2_ by deoxygenated hydroboration.

The amino group is ubiquitous in nature and there are a variety of methods available for the synthesis of amines. Since the conversion of the NO_2_ group to amine is a typical organic transformation and it generally involves alkali metals, NaBH_4_, hydrazine, and H_2_. These are the traditional reducing agents which are used for nitro to amine transformations. However, in recent times, some other robust catalysts have been reported. For instance, Zeng et al. in 2021, applied cAAC−Cr catalyst (**20**) in the presence of HBpin (pinacolborane) as a hydrogen donor via deoxygenated hydroboration reaction.^[71]^ Here, HBpin acts as a umpolung reagent, and cAAC−Cr complex promotes the reversal in polarity. They have also compared the reactivity of cAAC−Cr, NHC−Cr, cAAC−Cr−cAAC, NHC−Cr‐NHC, and bis(imino)Cr complex as well as 3d transition metal salts FeCl_3_, CoCl_2_, NiCl_2_, CuBr_2_ and PdCl_2_ in the presence of Mg as reducing agent. In the case of metal salts very poor yield of product was obtained while other metal complexes gave a moderate yield. The best yield was obtained in the case of cAAC−Cr complex with the turnover number (TON) of 1.8×10^6^. A variety of substrates like nitroarenes, heteroarenes, and nitroalkanes have been tried with HBpin (5 equiv.), catalyst 1 mol %, Mg (10 mol %) in THF (2 mL), 60 °C, 24 h, that gives ∼65–99% isolated yields. The optimized reaction conditions produce an excellent yield of amine suitable for pharmaceutical applications (Scheme 8).

**Scheme 9:**
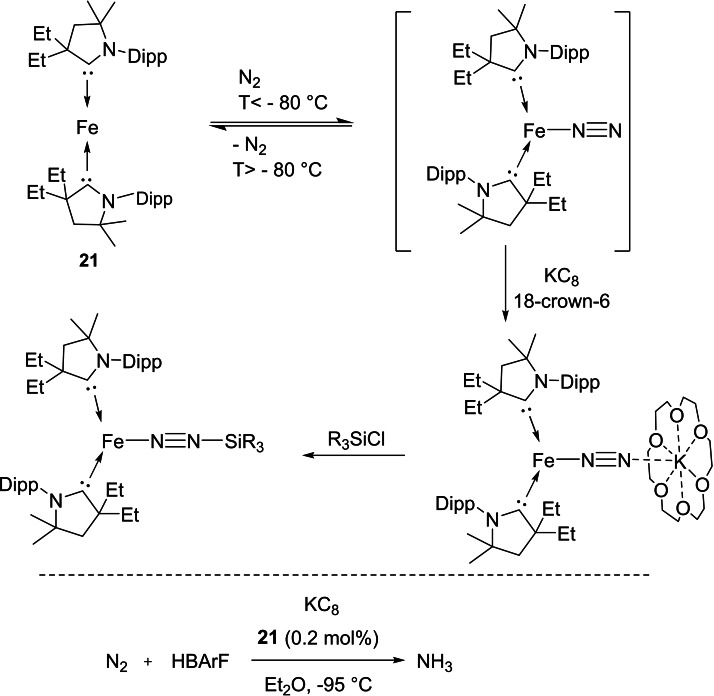
Reduction of (cAAC)_2_−Fe(0) (**21)** and dinitrogen to ammonia conversion catalyzed by **21**.

Interestingly, other cAACs‐d^8^ transition metal complexes also show catalytic activity. For instance, Peters and co‐workers have synthesized an iron(0) complex, (cAACs)_2_Fe^0^, **21** (Scheme 9) which has the ability to coordinate with N_2_ at low temperature and further undergoes reduction with KC_8_ in presence of crown ether. The reduced form of **21** can be trapped by silylating agents. Additionally, (cAACs)_2_Fe^0^ also catalyzes the formation of NH_3_ from the reaction of N_2_ with HBAr^F^_4_, respectively in the presence of KC_8_.^[72]^ In this process, the significant amount of N_2_ reduction to ammonia occurs below −78 °C which shows that this catalytic reduction is highly temperature‐dependent (Scheme 9). By now, transition metals were the only known centers to catalyze the activation and functionalization of dinitrogen. However, lately, Braunschweig et al. have shown that main group elements can also activate dinitrogen by a cAAC stabilized boron compound. Basically, they have shown that dinitrogen can be converted into ammonium chloride by a one‐pot, borylene‐mediated reaction that proceeds by a stepwise reduction‐protonation mechanism via an end‐on bridging N_2_ species.^[73]^ However, this reaction is not cyclic since one of the key steps is not spontaneous due to the high covalent character of the B−N bond.

**Scheme 10:**
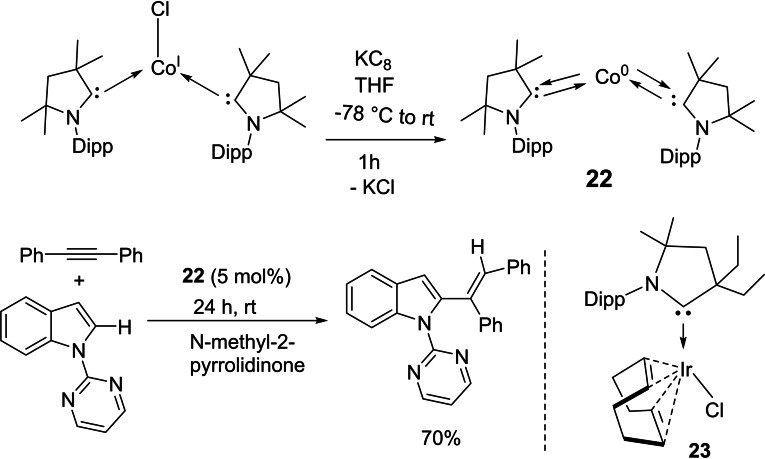
(top) Syntheses of **22** and (bottom) C_2_‐alkenylation of N‐pyrimidylindole with diphenylacetylene catalyzed by cAAC‐cobalt(0) complex (**22**) and the structure of cAAC stabilized Ir‐complexes (**23**).

The catalytic activity of cAACs‐d^9^ transition metals has been investigated.^[74]^ The Co(0)‐cAAC complex, (cAAC)_2_Co^0^ (**22**) has been used to demonstrate C_2_‐alkenylation of pyrimidylindole with diphenylacetylene and even cyclotrimerization of diphenylacetylene (Scheme 10, bottom).^[74]^

The catalyst **22** was prepared when a 1 : 1 molar mixture of (Me_2_−cAAC:)_2_Co^I^Cl and KC_8_ reacted in THF to give a dark blue solution, which gives dark shiny needles of (Me_2_−cAAC:)_2_Co^0^ with 98% yield on slow concentration under vacuum (Scheme 10, top).^[75]^ Deng et al. have also demonstrated the reactivity of cAAC‐cobalt complexes with organic halides.^[76]^ The heavier elements of the cobalt group have also been stabilized by cAAC ligands and have shown catalytic activity in commercially important conversions. For example, Zhang et al. reported a cAAC stabilized iridium complex (**23**) that shows efficient catalytic activity in methanol carbonylation to methyl acetate, at low temperature with high yield and selectivity.^[77]^ This iridium catalyst can be a potential alternative to the harsh reaction conditions for methanol carbonylation.

**Scheme 11:**
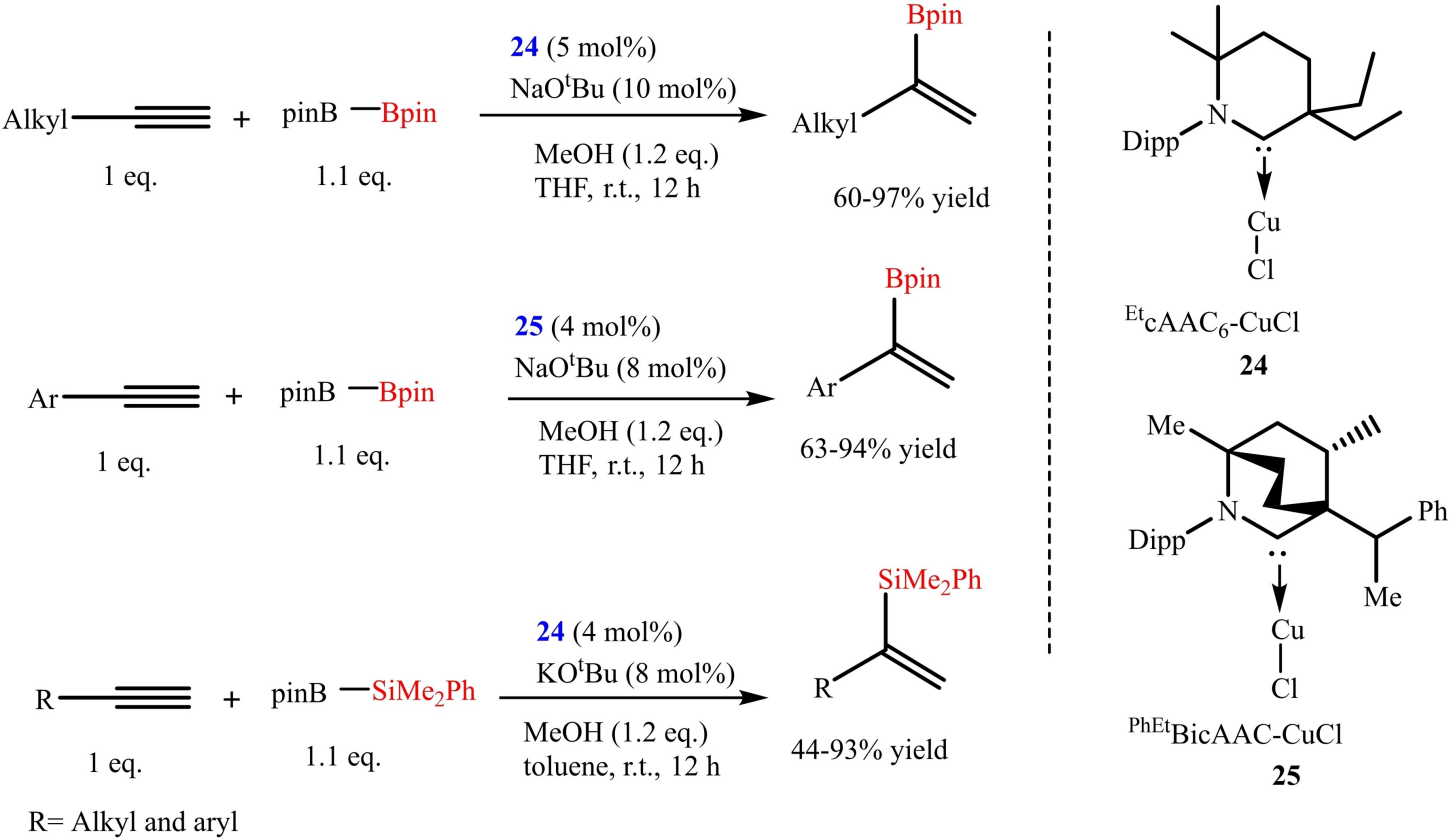
cAAC−Cu catalyzed hydroboration and hydrosilylation of terminal alkynes.

In order to synthesize alkyl borane and silane, the regioselective hydro functionalization of terminal alkynes is the easiest available approach but this protocol lacks selectivity and substrate scope. Therefore, developing a new protocol with good selectivity and wide substrate scope is desired. Gao et al. have synthesized a series of cAAC−Cu and NHC−Cu metal complexes for the selective hydroboration and hydrosilylation of terminal aliphatic and aromatic alkynes (Scheme 11). They found that cAAC−Cu complex (**24** and **25**) gave the better selectivity of the Markonikov selective hydrofunctionalized product with good to excellent yield.^[78]^ The reaction condition is highly tolerant for both alkyne as well as a variety of boryl and silyl reagents. To investigate the reaction mechanism authors performed competitive studies that revealed that a fine balance between the steric and electronic effect of the ligand is essential. In this regard, the most suitable ligand would be the one that is sterically flexible and a good electron donor.

**Scheme 12:**
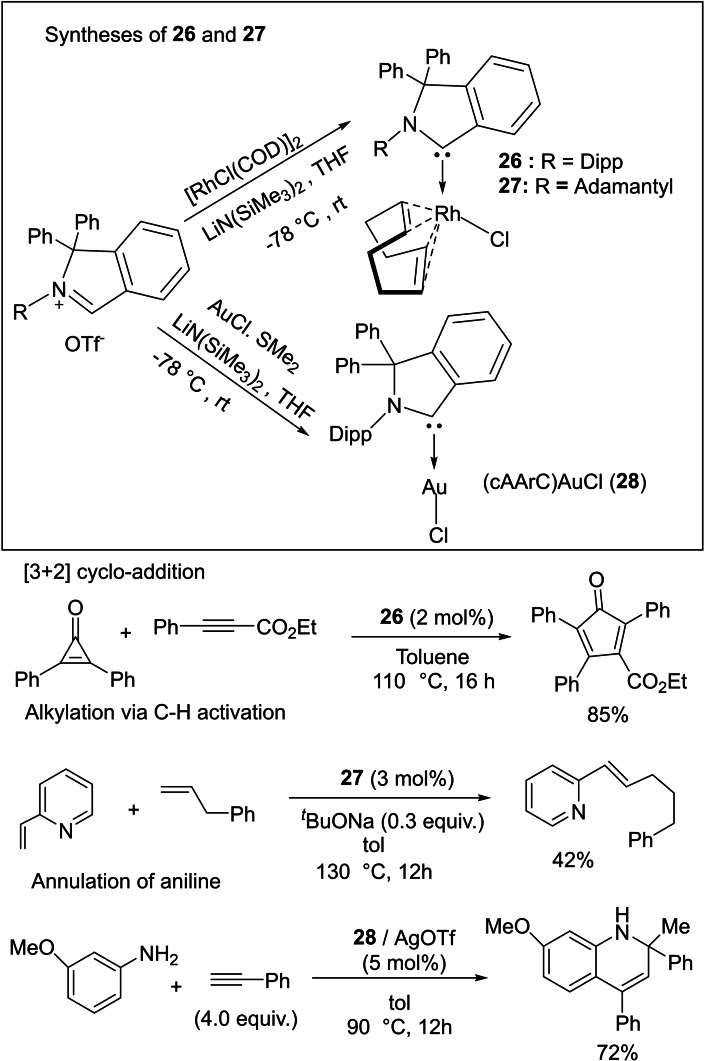
[3+2] cycloaddition, C−H bond activation and annulations of aniline catalyzed by cAArC ligated rhodium and gold complexes.

The substitution of the alkyl substituent of cAAC by an aryl group (Ar) gives increased electrophilic character to the carbene center, without affecting the high nucleophilicity of cAAC. Thus, cyclic(amino)(aryl)carbenes (cAArCs) have an even smaller singlet‐triplet energy gap than cAACs. Such singlet carbenes can be an efficient catalyst for several chemical reactions. The collaborative work of Zeng and Bertrand research groups reveals that (cAAC)rhodium complexes (**26** and **27**) catalyze the [3+2] cycloaddition of diphenylcyclopropenone with ethyl phenylpropiolate and also promote the addition of 2‐vinylpyridine to alkenes via C(sp^2^)−H bond activation (Scheme 12).^[79]^ Moreover, cAAC stabilized rhodium complexes and Rh(0) nanoparticles can catalyze chemoselective arene hydrogenation.^[80]^ It is important to note that (cAArC)gold complexes (**28**) also catalyze synthesis of 1,2‐dihydroquinolines from aniline and phenylacetylene.

**Scheme 13:**
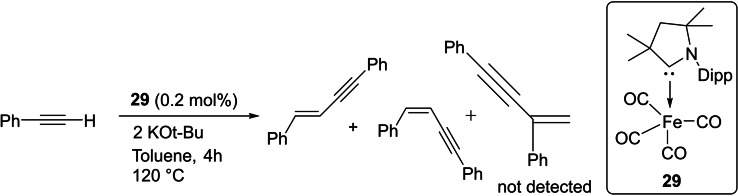
Dimerization of alkynes catalyzed by iron (0) complex (**29**) in toluene.

Some carbene complexes have been prepared by replacing a CO ligand of metal carbonyls, for example, Mandal et al. reported the synthesis of (cAAC)Fe(CO)_4_ (**29**) from Fe_2_(CO)_9_. This carbene complex (**29)** is reported to catalyze the dimerization of aryl acetylenes with E: Z ratio varying from 5 : 95 to 82 : 18 depending on the type of alkyne used: TONs were attained up to 6500 (Scheme 13).^[81]^ cAACs‐stabilized bi‐coordinated nickel complexes^[82]^ have also shown catalytic activity in the homocoupling of various unactivated aryl chlorides and fluorides with good yields of biaryls at moderate temperature.

**Scheme 14:**
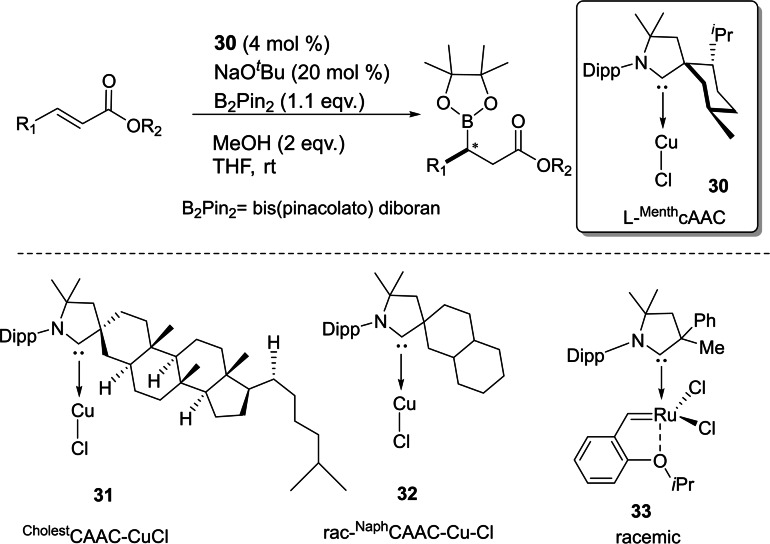
asymmetric conjugate borylation reaction catalyzed by **22** (top) and chiral Cu/Ru−cAAC complexes (bottom).

Asymmetric catalysis is an important aspect of synthetic chemistry in which the stereochemistry of the product can be controlled. However, cAAC‐based ligands and complexes have been utilized in several catalytic reactions but asymmetric catalysis by chiral cAAC ligands has not been mentioned. Most recently, Bertrand et al. have reported the first example of chiral cAAC ligands in enantioselective catalysis^[83]^ including asymmetric conjugate borylation (Scheme 14). Additionally, Bertrand's group has prepared an enantiopure L‐^Menth^cAAC (**30**) by enantio‐ or diastereoselective separation from the inexpensive (−)‐menthol. The key step of this synthesis is the propagation of the fact that the relatively bulky reactants approach the cyclohexane group selectively from the equatorial direction. However, in the recent work, Bertrand et al. have synthesized several chiral cAAC ligands such as ^Cholest^cAACCuCl (**31**) and rac‐^Naph^cAAC (**32**).^[83]^ These ligands have also been used in asymmetric catalysis. In a subsequent report, Bertrand et al. reported the isolation of cAAC stabilized ruthenium complexes (**33**), which shows excellent catalytic efficiency in asymmetric olefin metathesis and exhibits high enantioselectivity.^[84]^ The comparative study of metathesis reactions catalyzed by cAAC and NHC stabilized ruthenium complexes by Kaczanowska et al. shows that the efficiency and selectivity arehighly dependent on the types of carbenes.^[85]^ It has also been shown that the cAAC stabilized ruthenium‐based complexes can be employed in light‐activated^[86]^ and amine‐ assisted olefin metathesis.^[87]^

**Scheme 15:**
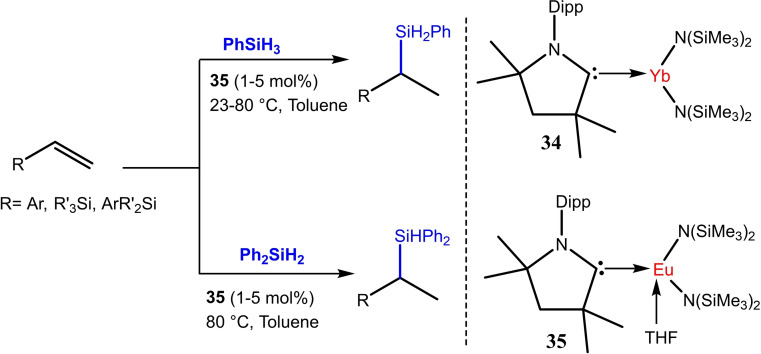
Hydrosilylation reaction catalyzed by cAAC‐stabilized Eu catalyst.

cAAC stabilized lanthanoids have also been reported that show catalytic properties. The first cAAC‐stabilized lanthanide complexes (cAAC)Yb[N(SiMe_3_)_2_]_2_ (**34**) and (cAAC)Eu[N(SiMe_3_)_2_]_2_(THF) (**35**) were reported by Cui et al. in 2021. These complexes catalyze the hydrosilylation of alkenes.^[88]^ The complex (cAAC)Eu[N(SiMe_3_)_2_]_2_(THF) regioselectivity catalyzes the hydrosilylation and gives Markovnikov product in high yields (Scheme 15).

**Scheme 16:**
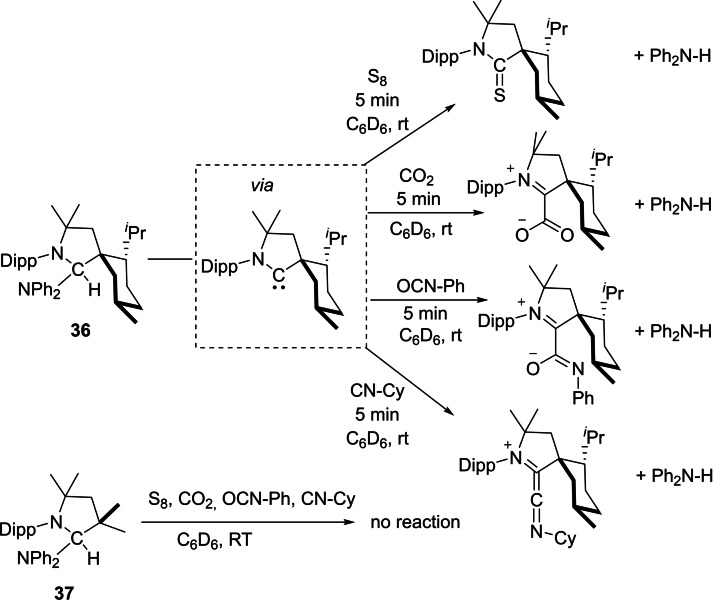
Reductive elimination of Ph_2_NH occurs from the sterically hindered **36** but not from **37**.

The ability of transition metals to switch between oxidations states is the basis of transition metal catalysis. Oxidative addition and reductive elimination are greatly influenced by steric hindrance around the metal center as well. A non‐metal equivalent to metal serving the same purpose was in search for a long time. In the meantime, it was realized that cAACs can undergo oxidative addition of strong E−H bonds (E=H, B, N, Si, P).[^14^, ^89^, ^90^] However, reductive elimination catalyzed by cAACs was not reported until Bertrand et al.^[91]^ showed cAACs can also promote reductive elimination at the carbon center just like transition metals. Bertrand et al. showed that cAACs with higher steric hindrance around carbene carbon promote reductive elimination but less hindered carbene does not promote reductive elimination (Scheme 16). The metal‐like properties of cAACs are observed due to the presence of vacant orbital and a lone pair of electrons on carbene carbon. Reductive elimination of Ph_2_NH happens from the sterically hindered **36** but not from **37**. Similarly, reductive elimination of Ph_2_P−H also occurs from the sterically crowded **36** but not from **37**.

### 3.2 Activation of Strong Bonds and Metal‐Free Synthesis Using cAACs

**Scheme 17:**
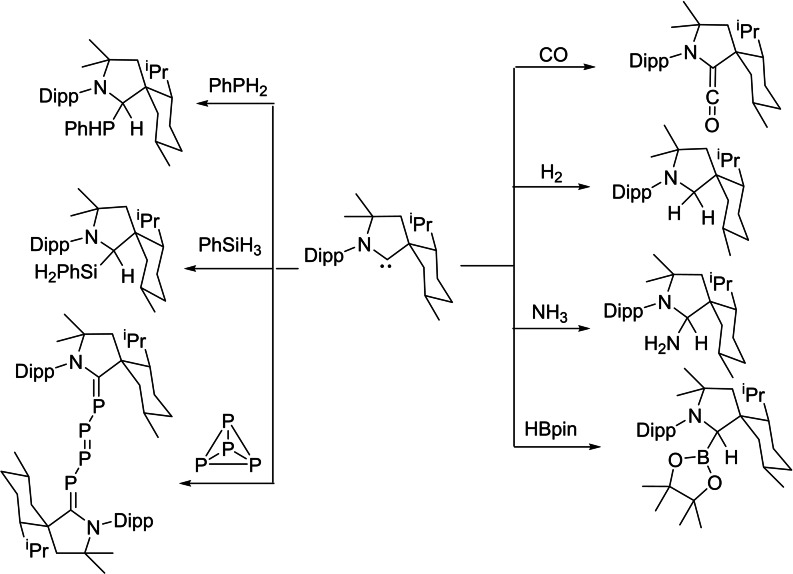
Activation of small molecules and strong bonds using metal‐free cAAC.

NHCs have been established as excellent catalysts for organocatalyzed transformations. The vast variety of organocatalyzed transformations mediated by NHCs proceeds via the process of umpolung. Pioneering work by Breslow on the thiazolium promoted benzoin condensation has demonstrated that NHCs function as excellent organocatalysts.^[92]^ On the other hand, cAACs cannot be used as organocatalysts due to their higher basicity and poor leaving ability. However, studies have shown that the divalent carbene carbon in cAACs can undergo insertion into E−H σ‐bonds similar to the transition metals, leading to the formation of new, strong C−E and C−H bonds (E=H, N, Si, B, P, C, O). Therefore, subsequent changes of products will enable them to be used in metal‐free synthesis. Seperately, due to excellent electrophilic character and smaller singlet‐triplet gap, cAACs, under mild conditions, can activate small molecules such as H_2_,^[14]^ CO^[22]^ and P_4_[^93^, ^94^] and even enthalpically strong σ‐bonds like those of Si−H, P−H, B−H, and N−H (Scheme 17).[^15^, ^95^]

**Scheme 18:**
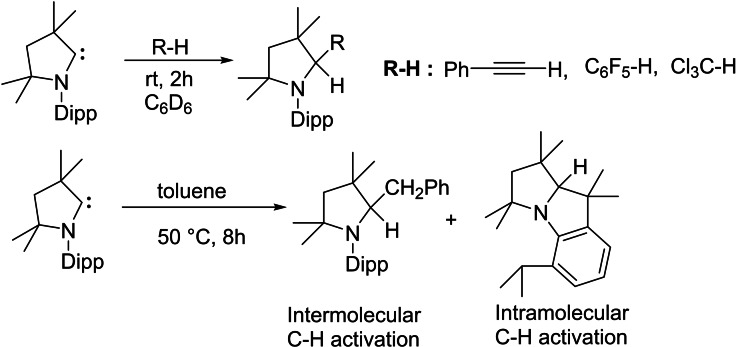
C−H bonds activation by cAACs.

Fundamentally, these results are encouraging and demonstrate the ability of cAACs to undergo oxidative addition similar to transition metals.^[96]^ The metal free catalytic organic transformations are of great demand due to the high toxicity of metal. The metal atoms/ions remains in some form as an impurities in the organic products which can not even be avoided by column chromatographic purification. Bertrand group was first to report that bulky cAACs react with CO resulting in stable ketene. This reaction was striking since Arduengo et al., through computational calculations, have denied the possibility of ketene formation.^[97]^ Despite the high strength of C−H bonds, recent reports show that cAACs can also activate some sp‐, sp^2^‐ and even sp^3^‐hybridized C−H bonds, under mild conditions (Scheme 18).^[98]^ These reactions involve nucleophilic attack of cAACs onto the rather acidic C−H bonds (pKa=16–29).^[14]^ Interestingly, toluene (pKa=43) being the least acidic substrate of the series, also undergoes inter‐ and intramolecular C−H bond insertion when heated in the range of 50–110 °C. These collective results indicate that cAACs can undergo oxidative additions similar to transition metals. Sadly, no viable catalytic system has been developed for such reactions until now, due to the high strength of C−H bond formation during this process.

### 3.3 cAAC Chemistry of s‐Block Elements

**Scheme 19:**
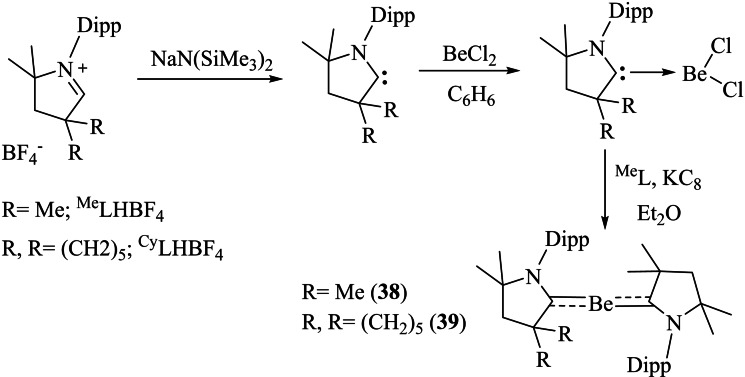
Stabilization of zero valent beryllium by cAAC ligands.

Due to the superior electronic properties of cAAC ligands, several transition metals have been stabilized in lower and even in zero oxidation states. These low valent transition metal complexes have found their usefulness as reagents as well as catalysts. The p‐block elements in their lower oxidation states have also been stabilized and their stable compounds have been isolated. However, cAAC stabilized s‐block elements in lower oxidation states are still a rarity. Stabilizing s‐block elements is very difficult due to their high reactivity. Therefore, cAAC chemistry of s‐block elements is still wide open with tremendous possibilities for stabilization of unusual bonding modes and oxidation states. Thankfully, alkaline earth metals especially beryllium could have been stabilized as neutral zero‐valent and radical species. For instance, Arrowsmith et al. stabilized zero‐valent beryllium utilizing excellent σ ‐donor and π ‐acceptor properties of cAAC ligands (**38** and **39**; Scheme 19).^[99]^ The π ‐back donation ability of electron‐rich beryllium to the π ‐system of carbene plays a crucial role in stabilizing beryllium in the zero oxidation state. Recently, alkali metals have been reacted to yield a cAACs−CO_2_ adduct at room temperature consisting of cAAC‐stabilized alkali CO_2_^−^ and CO_2_^2−^ clusters.^[100]^ These clusters show interesting one‐electron reduction chemistry with Li, Na, and K metal, producing stable monoanionic radicals having general formula [M(cAAC−CO_2_)]_n_. Whereas, the two‐electron alkali metal reduction produces dianionic clusters having the general formula [M_2_(cAAC−CO_2_)]_n_.

### 3.4 cAACs in the Stabilization of Species with Low‐Valent Transition Metals

**Scheme 20:**
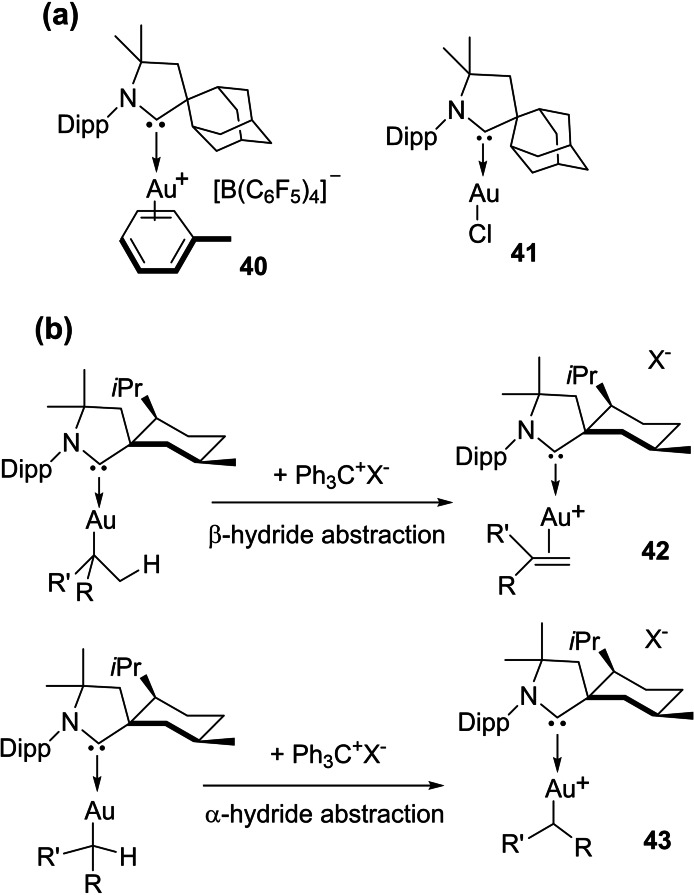
(a) Cationic gold(I) complexes (b) α‐ and β‐abstraction from cAACs stabilized gold(I) alkyl complexes.

Transition metal complexes, in which metal is capable of adopting variable oxidation states have been utilized extensively for different means of chemistry, especially in catalysis. Initially, transition metals have been stabilized in lower oxidation states with neutral ligands such as phosphines (PR_3_), carbon monoxide (CO), and olefins. However, the coordination numbers of metals in such complexes are higher since they tend to follow 18 electron closed‐shell configurations.^[75]^ Therefore, stabilization of metals in lower oxidation states and at the same time with lower coordination numbers has been an uphill task for chemists. A special ligand field electronic effect was missing for quite some time. Despite these challenges, chemists have been successful in synthesizing cAACs stabilized complexes with low valent transition metals. In 2005, Bertrand et al.^[101]^ stabilized low valent Pd and Rh complexes that followed isolation of several low valent complexes of other transition metals. For an instance, the solvent coordinated cationic gold(I) complex (**40**) was synthesized by the abstraction of halide from parent chloride complex, [AuCl(cAAC^ad^)] (**41**).^[33]^ In complex **40** the aromatic ring of bitentatively coordinated toluene is slightly perturbed, as shown by the X‐ray diffraction experiment, so the complex may be called pseudo‐naked [Au(cAAC)]^+^ cation. Interestingly compound **40** is stable for a long time in the solution as well as in solid‐state and exhibits high thermal stability. Hence, it is a well‐ qualified species for catalytic applications for unusual organic transformations. One of the common features these complexes have is that they require stronger σ‐donation and steric hindrance from the ligands. The latter helps the final product to dissociate away from the catalyst at the end of the catalytic cycle. This can be achieved by using very bulky tertiary phosphines (e. g., JohnPhos)^[102]^ or carbenes (e. g., cAAC^adamantyl^).^[103]^ Furthermore, alkylgold(I) precursor [Au(butyl)(cAAC^menthyl^) undergoes β‐hydride abstraction to give cationic cAAC‐stabilized alkene complex (**42)**. Unlike NHCs, in such reactions, β‐hydride abstraction takes place due to the strong σ‐donor properties of cAACs. Interestingly, α‐hydride abstraction that gives a new bis‐carbene‐stabilized gold(I) complex (**43**) is also possible in such complexes owing to the σ‐donor properties of cAACs (Scheme 20).^[33]^

Several cAAC‐ligated mononuclear complexes have been reported until now and are being applied in different fields of chemistry. Moreover, Bertrand et al. have reported the synthesis of cAAC ligated multi nuclear‐mixed valence Au(I)−Au(0) and Au(I)−Au(I) clusters which can catalyze carbonylation of amines.^[104]^ Furthermore, they have also reported homodimetallic Cu(I)‐alkyne and triazole complexes which help to rationalize elementary steps of the catalytic cycle of Cu‐cAAC assisted click reactions.^[53]^

**Scheme 21:**
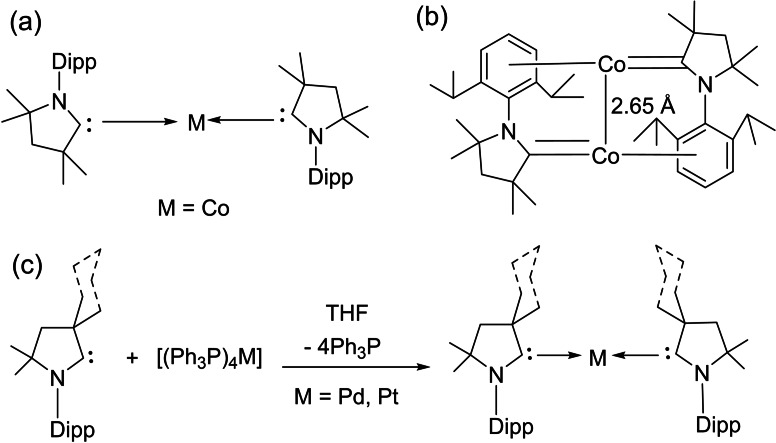
(a) Bis(cAACs) complexes of different metals (b) cAAC stabilized cobalt‐cobalt bond having distance of 2.6550 Å. (c) Synthesis of cAAC – stabilized Pd(0) and Pt(0) complexes.

Braunschweig et al. reacted cAACs‐stabilized copper, gold, and silver cations to an anionic dimetalloborylene [(η5−C_5_H_5_)(CO)Mn=B=Mn(CO)(η^5^−C_5_H_5_)]^−^ that gives a heterodimetallic compound. Notably, cAACs have found several important applications with transition metals.The cAAC ligands give an access to the bis‐cAAC‐stabilized diamagnetic and paramagnetic metal complexes with the metal atoms in lower or even zero oxidation states (Scheme 21, a). Such low valent and low valence [M(L)_n_]^n+^ complexes can play an important role as intermediates in catalytic conversions since they have vacant coordination sites at the metal centers. Thus synthetic access was desired for a long time. Isolation of such cAAC‐M species has been very crucial. Different research groups have reported the synthesis of bis (cAAC)‐complexes of cobalt, iron,^[105]^ copper,^[106]^ gold,^[107]^ chromium,^[108]^ manganese,^[109]^ nickel,^[82]^ and zinc^[110]^ complexes. Moreover, we have also shown that bivalent palladium and platinum metal can be stabilized by cAACs in zero oxidation states (Scheme 21, b).^[111]^ These bivalent complexes, [(cAAC)_2_Pd/Pt] with Pd(0) and Pt(0) can be synthesized by reacting cAAC with tetrakis(triphenylphosphine)palladium(0) and the equivalent Pt(0) precursor.^[111]^ It is interesting to note that [(cAAC)_2_Pd] complex shows crystallochromism, changing color from dark maroon to bright green owing to the bending of the C−Pd−C bond angle from 172.75(6)^0^ to166.94(6)^0^.^[111]^ As we know, the realization of metal complexes with zero or low‐valence metal atoms is a challenging task that demands skilled hands and sophisticated laboratories. To the list of existing low valent cAAC‐complexes of transition metals, Deng et al. have added an interesting account of tricoordinate zero‐valent cobalt, iron, and manganese complexes stabilized by cAAC ligand.^[112]^ The stabilization of a covalent cobalt‐cobalt bond has also been achieved by utilizing cAAC ligand; where cobalt possesses zero formal oxidation state.^[113]^ This is quite a remarkable observation. Additionally, coordinate low‐valent Cu(I) and Au(I) have also been stabilized by BicAAC.^[114]^

**Scheme 22:**
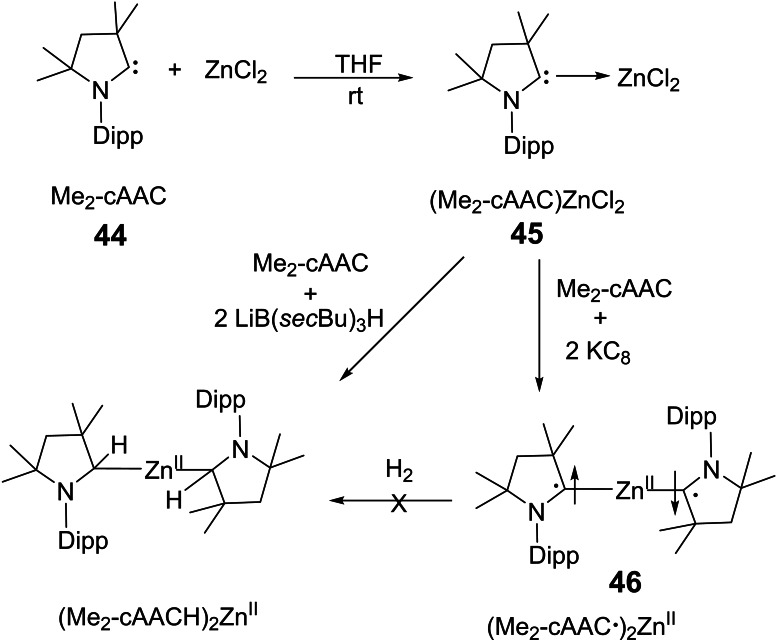
Syntheses of cAAC stabilized Zn(II) complexes.

**Scheme 23:**
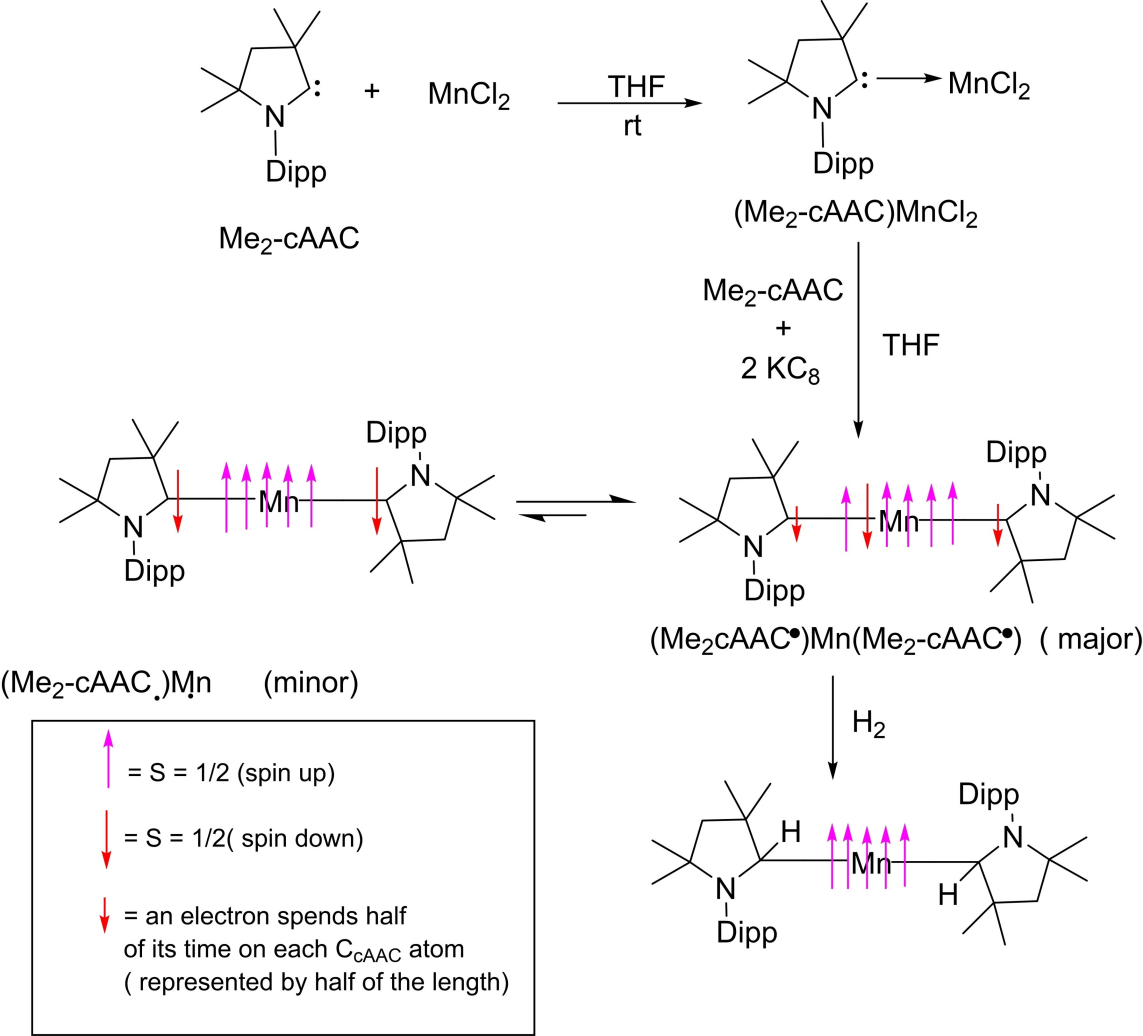
Synthesis of cAAC‐stabilized Mn complexes(L−Mn−L) and facile splitting of dihydrogen by L−Mn−L.

In order to synthesize (cAACs)_2_M complex (M=Mn and Zn), we initially prepared an adduct (Me_2_−cAAC)MCl_2_ by reacting Me_2_−cAAC (**44**) with anhydrous M(II)Cl_2_ in 1 : 1 molar ratio in THF (Scheme 22 and 23). Here (Me_2_−cAAC)ZnCl_2_ (**45**) was reduced in the presence of two equivalents of KC_8_ to form dark blue colored (Me_2_−cAAC⋅)Zn^II^ (**46**) (Me_2_−cAAC⋅=radical anion on carbene carbon atom) or dark purple solution of (Me_2_−cAAC)_2_Mn in low yield. The yield increases when the reaction is performed in the presence of one more equivalent of Me_2_−cAAC.^[110]^ The spectroscopic studies reveal that (Me_2_−cAAC⋅)_2_Zn^II^ is EPR inactive whereas (Me_2_−cAAC)_2_Mn is EPR active. Moreover, (Me_2_−cAAC)_2_Mn is capable of activating H_2_.^[109]^

**Scheme 24:**
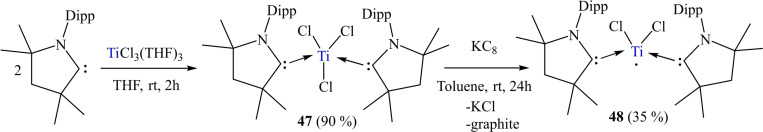
Synthesis of cAAC‐supported Ti(III) chloride complexes (**47** and **48**).

In 2008, the first homoleptic cationic bis‐cAACs‐coordinated gold(I) complex was reported^[115]^ and subsequently a homoleptic neutral bis‐cAAC‐stabilized complex was also published in 2013.^[110]^ Additionally, Bertrand and Roesky et al. have even reported homoleptic dinuclear complexes such as [Co(cAAC)_2_]^[13]^ and [Au_2_(cAAC)_2_].^[116]^ Ma et al. have synthesized a mononuclear cAAC‐supported titanium(III) chloride complex [(cAAC)_2_TiCl_3_] (**47**)^[117]^ by treatment of free cAAC with TiCl_3_(THF)_3_. Further reduction of **47** with potassium graphite (KC_8_) affords (cAAC)_2_TiCl_2_ complex (**48**), which features a small singlet‐triplet energy gap (Δ E_S/T_) (Scheme 24).

### 3.5 cAAC Chemistry of Group 13 elements

**Scheme 25:**
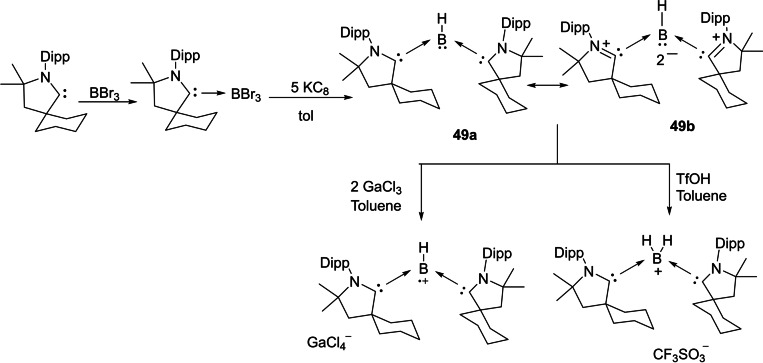
Synthesis of borylene‐ bis(cAAC) adduct (**49**) and its subsequent oxidation into a radical cation.

Group 13 elements, in general, adopt +3 oxidation states in most of the cases hence producing electron‐deficient compounds. To compensate for electron deficiency, they promptly form adducts and undergo hyper‐valent bonding, therefore they act as Lewis acids. In the last decade, several cAACs have been reported to form adducts with group 13 elements. However, cAAC‐chemistry with boron has been extensively explored. Probably, one of the prominent uses of cAACs in boron‐chemistry was to synthesize tricoordinate organoboron compounds **49 a** and **49 b** by the reaction of a parent borylene with the cAAC, in which on boron features +1 oxidation state. Noteworthy is the fact that compound **49** is valence isoelectronic with amine and phosphine and it can be further oxidized to give the corresponding stable radical cation and it can also be protonated to give conjugate acid (Scheme 25).^[118]^ Parent borylene, due to the presence of two vacant orbitals and a lone pair of electrons on the boron atom is highly reactive, and therefore, it forms borylene‐biscAAC adduct, BH(cAAC)_2_. The ^1^H‐decoupled ^11^B NMR spectrum of **49** shows a broad resonance at 12.5 ppm and a half‐width of 261 Hz. The cyclic voltammogram suggests that BH(cAAC)_2_ can be oxidized to it corresponding radical cation.^[118]^ Thus boron species have been compared with N‐compounds and shown to form radical cation similar to the N‐containing species.

**Scheme 26:**
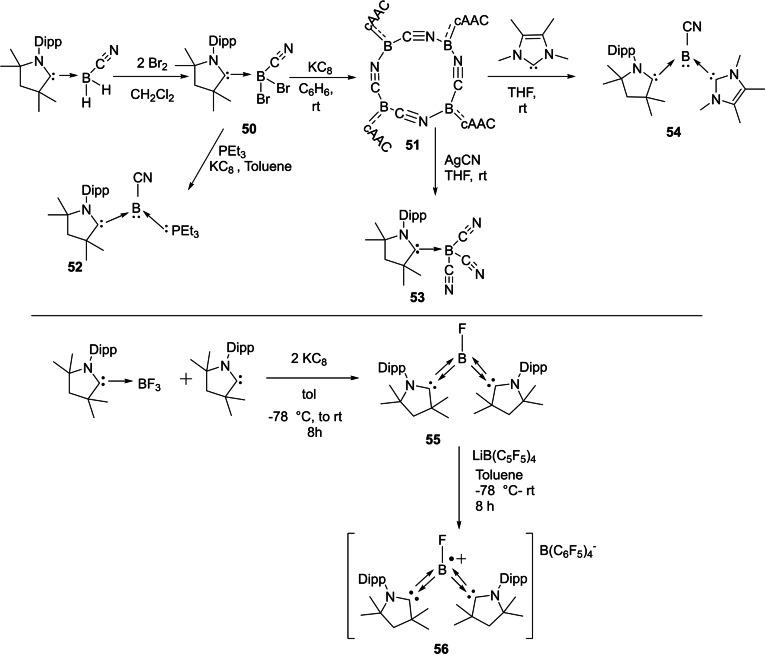
Isolation of cAACs stabilized borylenes.

Braunschweig et al. have presented interesting and profound experimental results in the cAAC chemistry of boron. Recently, they have shown that reduction of a cAAC−B(CN)Br_2_ adduct **50** at room temperature in the presence of triethylphosphine gives (cAAC)(PEt_3_)BCN **52**.^[119]^ Interestingly, reduction of cAAC−B(CN)Br_2_ in the absence of phosphine led to the formation of cyanoborylene tetramer **51** (Scheme 26).^[119]^ The reaction was performed at rt in the presence of excess KC_8_ in benzene and a deep blue suspension, which turned dark red over time, was obtained. Further filtration, extraction, and solvent removal have been carried out to obtain a red‐colored compound **51**. ^11^B‐NMR spectrum of **51** shows a single broad resonance at −4.16 ppm. It has been observed that compound **51** upon addition of NHC produces (cAAC)(NHC)BCN derivative (**54**). Surprisingly, the same reaction with analogous bis(NHC)B_2_ results in decomposition.^[120]^ Most recently, our research group reported synthesis and isolation of cAAC stabilized fluoroborylene ((Me−cAAC)_2_BF) (**55**)^[121]^ and its radical cation [(Me−cAAC)_2_BF]^.+^[B(C_6_F_5_)_4_]^−^ (**56**) which have been fully characterized by single‐crystal XRD, multinuclear‐NMR spectroscopy, cyclic voltammetry and EPR spectroscopic studies (Scheme 26, bottom).

**Scheme 27:**
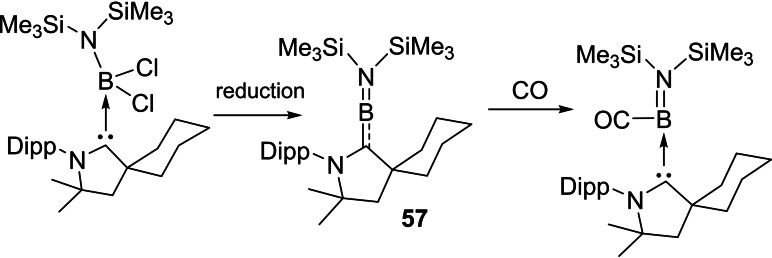
Linear metal‐free borylene.

Quite recently, some more exciting results in borylene (:BR) chemistry, in which unique reactivity of boron atom in low oxidation state is explored; have been reported.[^122^, ^123^] Earlier, isolation of borylene was very difficult and thus limited to the corresponding transition metal complexes only; however, recent advances have enabled isolation of several metal‐free tricoordinate borylenes (L_2_BR) that exhibit significant nucleophilicity at the boron atom.[^124^, ^125^] In contrast to that, mono‐ and most di‐coordinated borylenes are transient species that can only be generated in harsh reducing conditions and can only be characterized spectroscopically. In this context, Bertrand's research group has reported the first linear metal‐free amino‐borylene compound [(^cy^cAAC)BN(SiMe_3_)_2_] (**57**) (Scheme 27) which can be considered as a zwitterionic hetero‐allene (C=B=N). Surprisingly, this compound was found to break molecular hydrogen and bind CO at B‐centre similar to the transition metals; however, exhibits reduced reactivity due to its push‐pull π‐electronic stabilization. **57** can also activate H_2_ molecule at B‐centre.

**Scheme 28:**
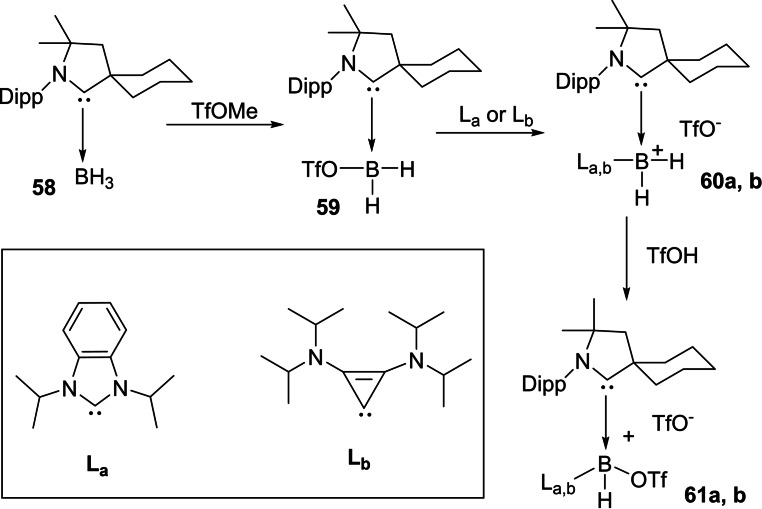
Synthesis of triflate derivatives of borylene.

Moreover, Braunschweig and co‐workers have also reported coordination of two equivalents of CO to a borylene fragment in a manner similar to that of transition metals.^[126]^ It is interesting to note that exposure of one borylene‐CO complex to the UV‐radiation prompts intra‐molecular C−C bond activation.^[127]^ Bertrand et al. reported the synthesis of an asymmetrically substituted derivative of borylene of type (L_1_)(L_2_)BH in which the boron atom is bound with the cAAC as well as triflate groups. For the synthesis of cAAC‐mono triflate borane complex **59**, cAAC‐borane complex **58** was reacted with the trifluoromethane sulfonate and white powder of desired product in 95% yield was isolated (Scheme 28)^[125]^ ^11^B NMR spectrum of **59** exhibits a broad single resonance at −6.1 ppm which shifted downfield from complex **58** (−28.5 ppm); additionally, ^19^F NMR spectrum shows a resonance at −76.2 ppm that confirms the presence of triflate group covalently bonded with boron. Interestingly, compound **59** on further reaction with carbene **L_a_** and **L_b_** forms the desired bis(carbene) brominium salts **60 a** and **60 b** which were isolated in 95 and 80% yields, respectively.^[125]^ In order to enhance the acidity of boron bonded protons, one more triflate group was added by reacting **60 a** and **60 b** with triflic acid. As a result, compounds **61 a** and **61 b** were obtained as white solids in high yields.

**Scheme 29:**
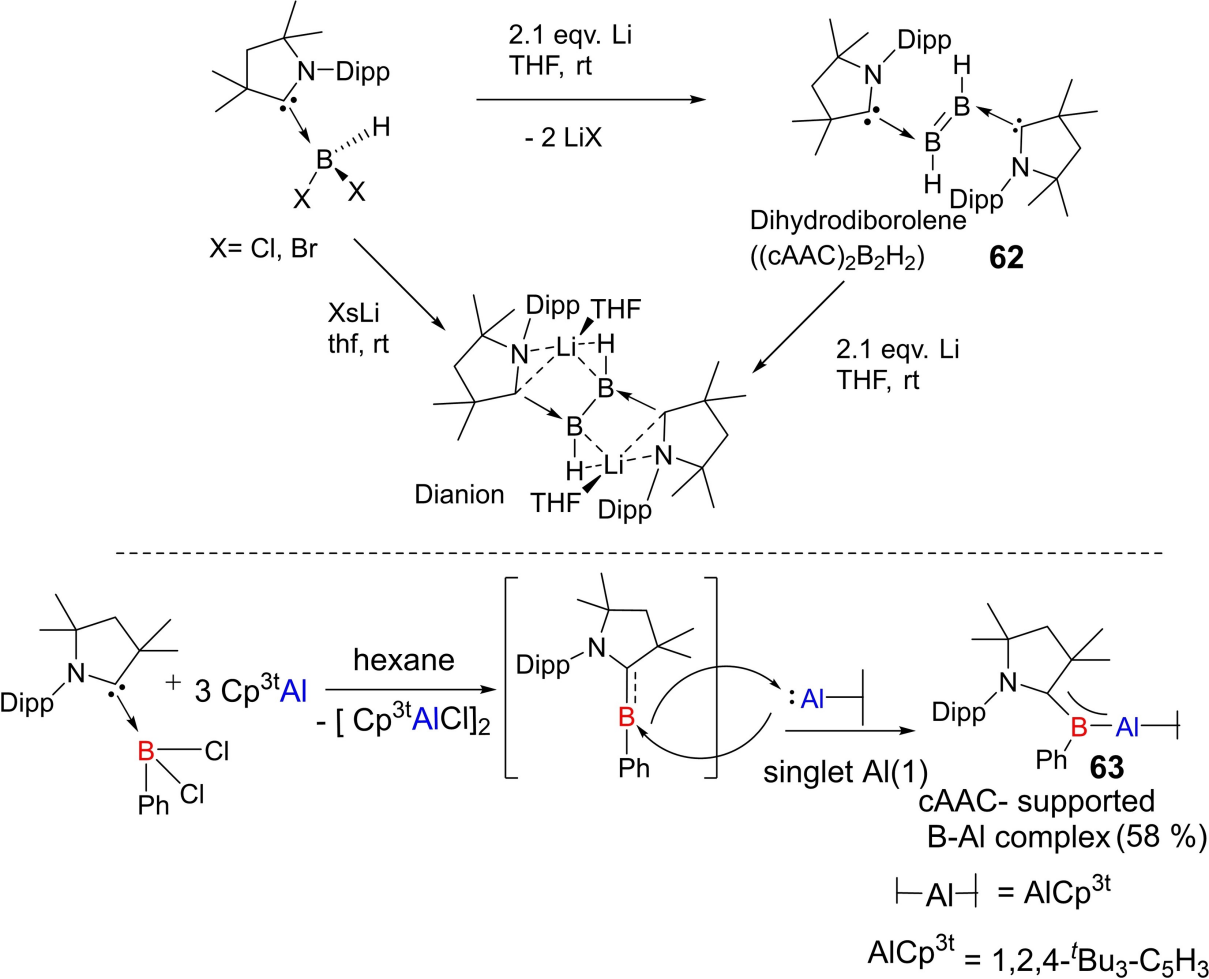
Isolation of dihydroborelene and its dianion (top) and cAAC‐supported B−Al complex.

Braunschweig et al., in an interesting result, reported that the reductive coupling of two cAAC‐supported dihaloboranes affords the formation of diborane (cAAC)_2_B_2_H_2_) (**62**). Moreover, they were also able to isolate a unique and highly sensitive (halo)hydroboryl anion intermediate, as well as the doubly reduced dianion of (cAAC)_2_B_2_H_2_, which displays formal B=C double and B−B single bonds(Scheme 29, top).^[128]^ They have also reported a facile synthesis of a stable dihydroboryl anion^[129]^ and borylene‐stabilized‐borylene.^[130]^ Al(III) compounds are commonly reported, however, Al(I)‐compounds are rarely known. Most recently, an interesting compound, Al(I)‐hydride has been stabilized by two cAAC ligands at room temperature.^[131]^ Subsequently, Braunschweig et al. in another publication reported a complex (**63**) which features multiple bonding between boron and aluminum, formed by the reversible association of two singlet fragments (Scheme 29, bottom).^[132]^ Wherein, the heteroatomic multiple bond is strongly polarized but involves B−Al p electron density. As a result, it reacts rapidly with CO_2_ to form a borylene CO complex and an aluminoxane.

**Scheme 30:**
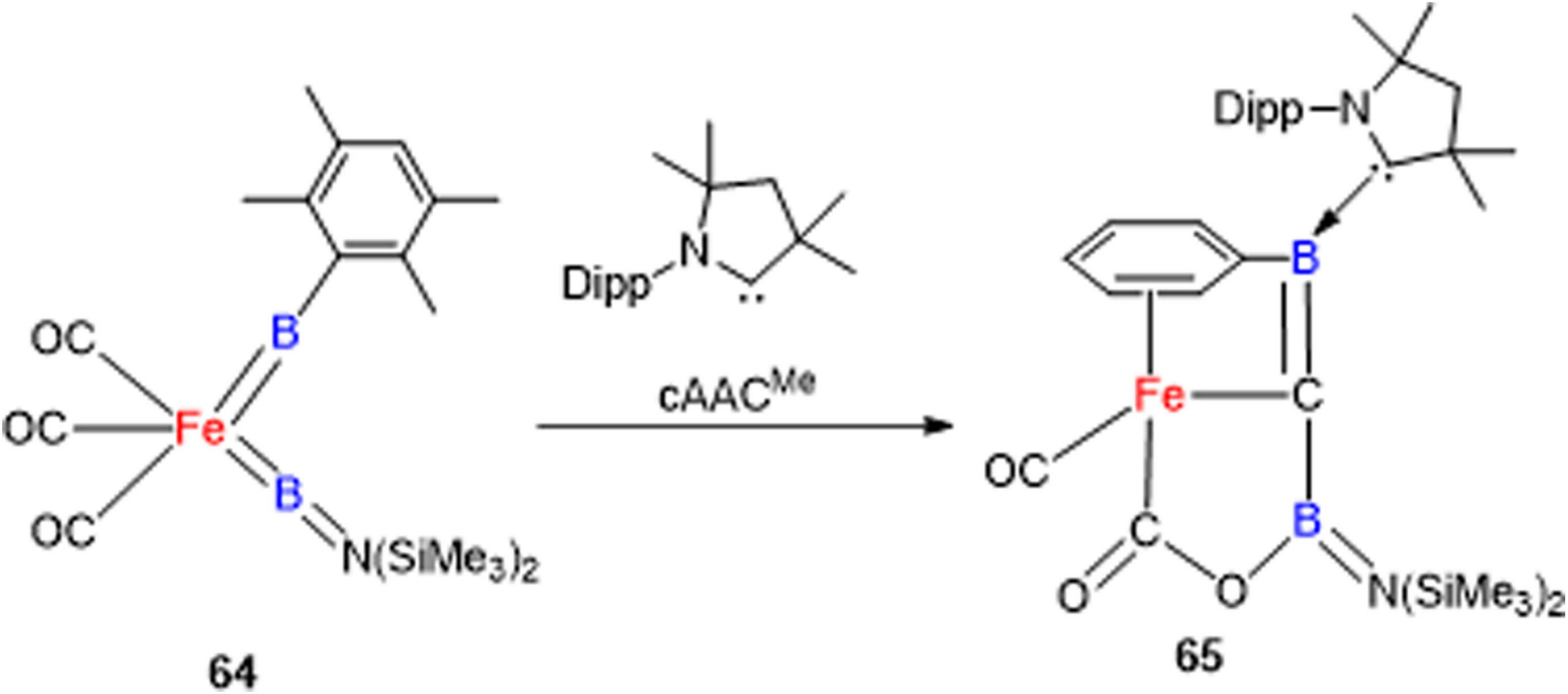
Synthesis of complex **65**.

Recently, a unique property of zero‐valent iron‐bis(borylene) complex (**64**)^[133]^ of cAAC, featuring highly selective intramolecular C−O bond scission of carbonyl ligand was reported. Unlike C−H activation and weaker single C−O bond cleavage by transition metal complexes, this was an unprecedented reaction by zero‐valent iron bis(borylene) complex featuring intramolecular cleavage of multiple C−O bond in carbonyl ligand at very mild conditions. Due to good σ ‐donor properties of cAAC, reaction of complex **64** with cAAC^Me^ in aromatic solvent led to the formation of an unusual iron complex **65** (Scheme 30).^[134]^ DFT calculations predict that two Lewis acidic borylene boron atoms help to cleave C−O multiple bond of the carbonyl ligand.

**Scheme 31:**
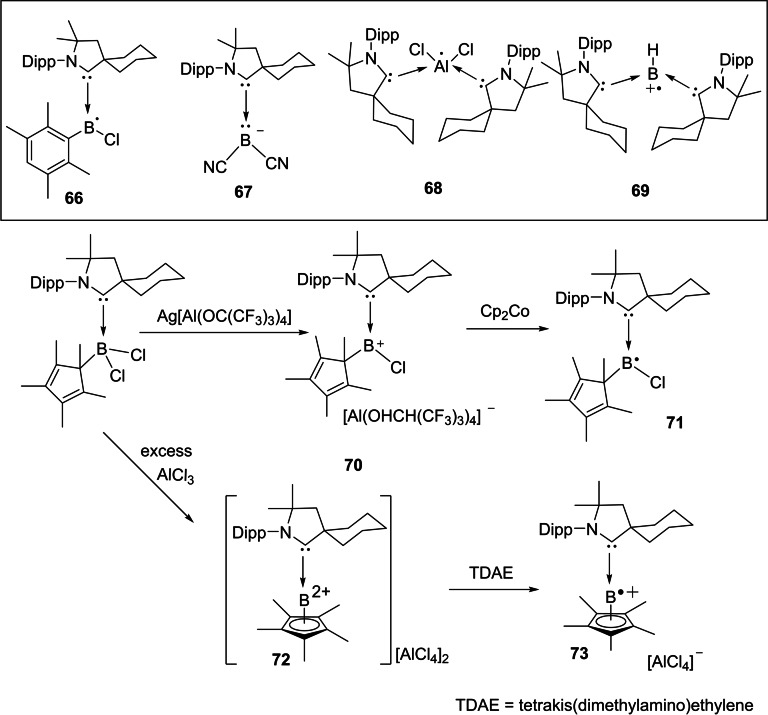
Group 13 element‐centered radicals, anions, and radical cations.

cAACs have not only been used to synthesize nucleophilic organoboron compounds but also in the stabilization of neutral boronmonochloride (**66**), boryl anion (**67**),^[135]^ aluminum radicals (**68**)^[136]^ as well as radical cations (**69**). Braunschweig and co‐workers have reported the synthesis of a neutral cAAC‐stabilized boron‐containing radical.^[136]^ Moreover, Chiu et al. have isolated cAAC‐stabilized boron cation (**70**) as well as dication (**72**). It has been shown that reduction of cAAC complexes (**70**) and (**72**) leads to the corresponding neutral radical (**71**) and radical cation (**73**) (Scheme 31).^[137]^ In a recent report, Rang et al. have shown that the cAAC stabilized borylene carbonyl complexes undergo one‐electron reduction to produce dimeric borylketyl radical anion, which in fact, is formed by the intermediacy of a radical anion.^[138]^

**Scheme 32:**
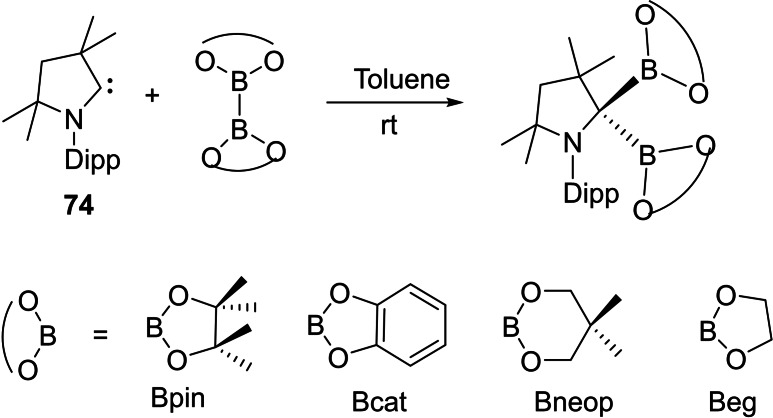
The reaction of cAAC^Me^ with diboron compounds.

Insertion of carbene carbon into B−B bonds have been reported by different research groups and a couple of reviews have also been published on this subject.[^139^, ^140^, ^141^] Most recently, Radius and co‐workers^[142]^ have reported the insertion of carbene carbon of cAAC into the B−B bond of different diboron compounds (Scheme 32). To demonstrate the insertion, cAAC^Me^ (**74)** was reacted with different diboron compounds including B_2_Pin_2_, B_2_Cat_2_, B_2_neop_2,_ and B_2_eg_2_ at room temperature. The immediate insertion of carbene carbon in B−B the bond was confirmed by in situ NMR experiment and complete reagent conversion was observed without any detectable intermediate or adduct. This irreversible insertion reaction is a convenient method for the preparation of C1‐bridged bisborates with a quaternary carbon atom.^[142]^

**Scheme 33:**
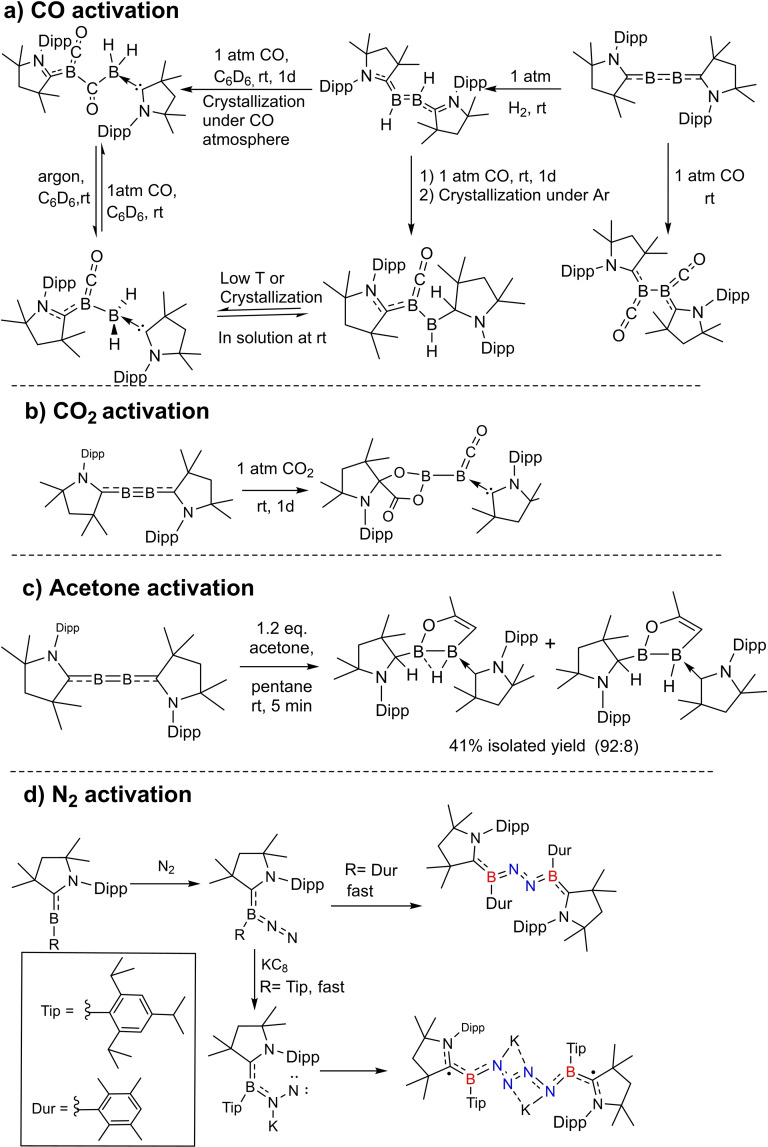
Activation of (a) CO (b) CO_2_ (c) acetone and (d) N_2_.

Braunschweig et al. have extensively explored the cAAC chemistry of boron which they have summarized in a review.^[143]^ Their recent work includes the isolation of compounds containing diboron flanked by two cAAC ligands (borylenes), which have been used in exotic chemical reactions. For instance, activation of CO,[^144^, ^145^] CO_2_,^[146]^ acetone^[147]^ and dinitrogen (Scheme 33).^[148]^ Zhang et al. have computationally studied the role of cAAC ligands in the activation of dinitrogen by borylenes.^[149]^

**Scheme 34:**
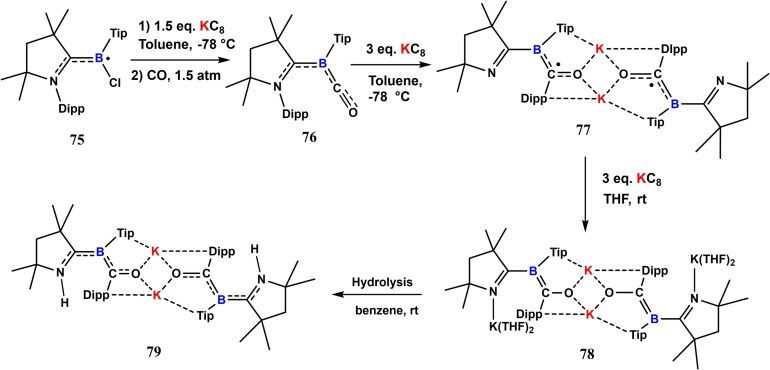
One and two electron reduction of borylene carbonyl complex.

Unlike transition metal carbonyls, Radacki et al. reported the conversion of a cAAC based boryl radical (**75**) to cAAC‐stabilized borylene carbonyl complex (**76**) in the presence of KC_8_ and CO. The further one‐electron reduction of **76** produces a novel dimeric borylketyl radical anion (**77**) by intramolecular Dipp group transfer to the carbonyl carbon.^[138]^ On further reduction with KC_8_ it gives a dimeric compound (**78**) with O−K−O linkage and on hydrolysis, it converts into a desired protonated dimer (**79**) (Scheme 34).

**Scheme 35:**
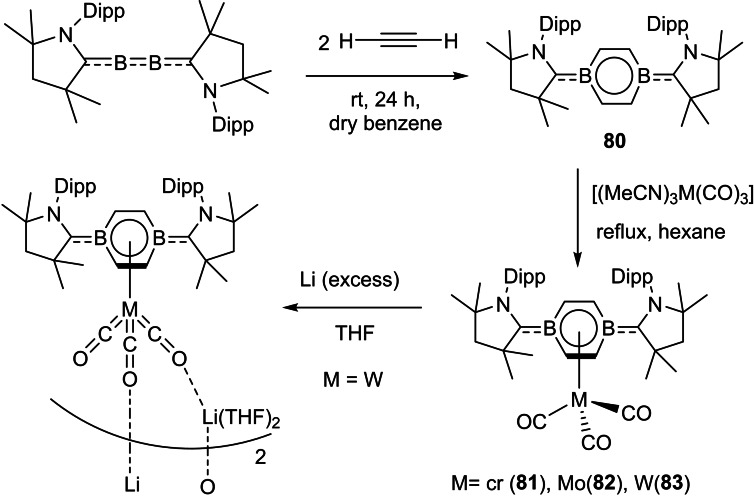
The synthesis of half‐sandwich compounds.

**Figure 6:**
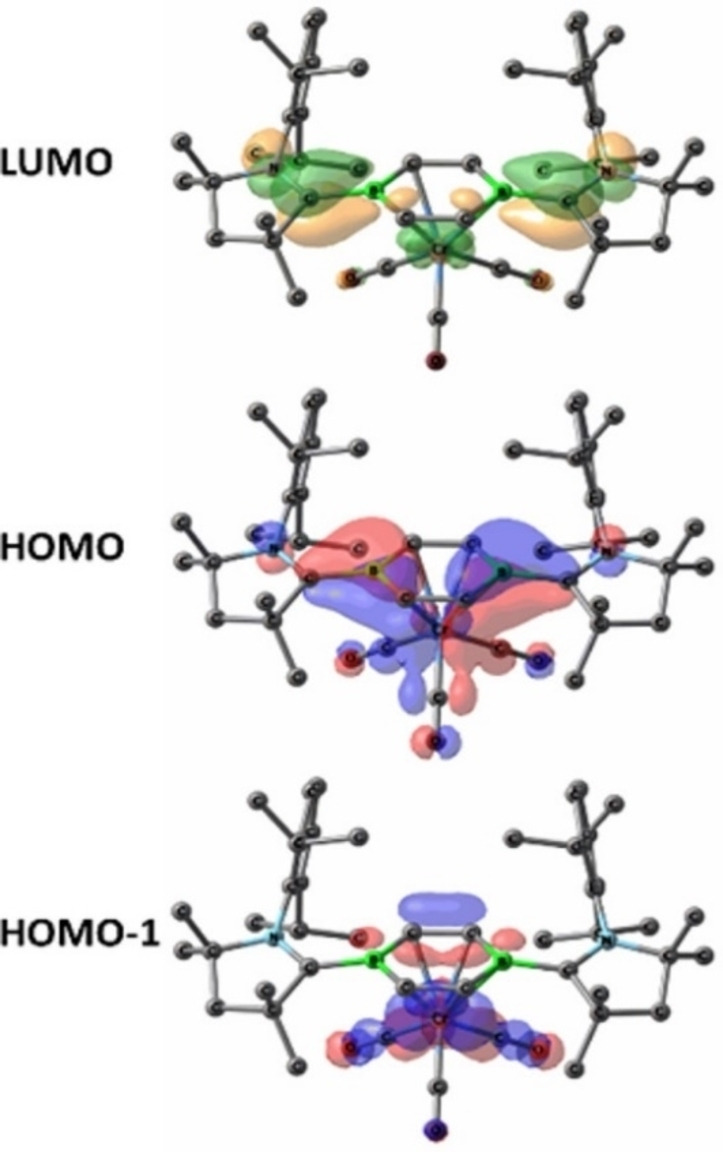
FMO plots of compound **81** calculated at the meta‐GGA M06‐L level of theory. Reproduced with permission from Ref. [150]. Copyright (2021) The Royal Society of Chemistry.

Böhnke et al. have also isolated half‐sandwich complexes of group VI with the diborabenzene ligand. The diborabenzene ligand (**80**) stabilized by cAAC is a comparatively stronger donor than its all‐carbon analogue, that is an evident from unprecedented lower carbonyl stretching frequency in neutral piano tool complexes (**81**, **82** and **83**) (Scheme 35).^[150]^ This can be further supported by the significant blue shift in the UV visible spectra of **81**–**83**. The absorption maxima appear at near 400 nm in the case of complexes that are highly blue‐shifted when they are compared to that of the diborabenzene ligand (633 nm). The ^11^B NMR signals in these complexes (6.0–7.0 ppm) are significantly upfield shifted compared to that of the free diborabenzene ligand (24.8 ppm). In order to gain insights into the bonding of complexes (**81**–**83**), DFT calculations were performed at the M06‐L/def2‐SVPD:PM_6_ level of theory to further investigate chemical bonding in these exotic complexes. The interaction energy (complexation energy) and binding energies have been calculated. For the sake of comparison, interaction and binding energies of the parent compound, [(η^6^−C_6_H_6_)Cr(CO)_3_] were also calculated at the same level. Binding energy is the negative of dissociation energy. The comparison between the parent compound, [(η^6^−C_6_H_6_)Cr(CO)_3_] and complexes reveals that the complexes have higher interaction and binding energies. For example, **81** has interaction energy −92.2 kcal mol^−1^ but [(η^6^−C_6_H_6_)Cr(CO)_3_] has −59.6 kcal mol^−1^. Similarly, binding energy for **81** is 82.9 kcal mol^−1^ but for [(η^6^−C_6_H_6_)Cr(CO)_3_] is 55.4 kcal mol^−1^. The interactions are evident from frontier molecular orbitals (FMO) plots (Figure 6). The HOMO reveals the interaction between metal center and B−C bond in the borabenzene ligand, however, HOMO‐1 describes the interaction of carbonyl groups with d‐orbitals of metal. The LUMO mainly represents the π‐system of the B−C−N moiety of the ligand. The LUMO orbitals are destabilized compared to the free borabenzene because there is no effective back donation from boron.

**Scheme 36:**
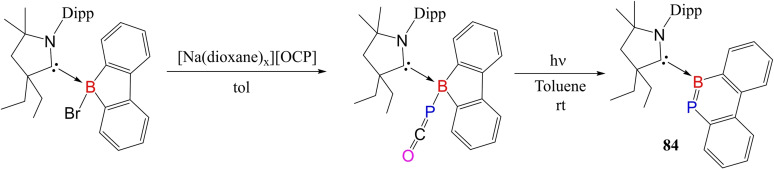
Synthesis of BP‐phenanthryne.

**Scheme 37:**
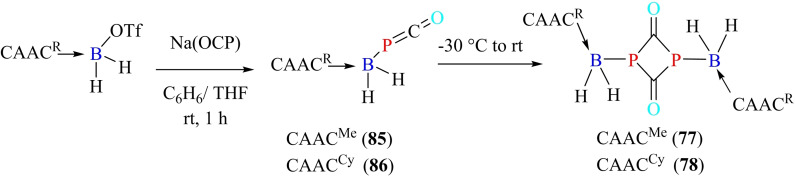
Synthesis of cAAC stabilized boraphosphaketenes and their dimers.

Yang et al. have been successful in isolating B−P doped phenanthryne (**84**) by utilizing the stabilizing effect of cAACs. They first synthesized boraphosphaketene, cAAC‐borafluorene‐P=C=O, which was finally photolyzed to BP‐phenanthryne (Scheme 36).^[151]^ Similarly, Hagspiel et al. synthesized cAAC‐stabilized boraphosphaketenes (**85** and **86**) by nucleophilic addition of cAAC‐stabilized triflatoboranes to sodium phosphaethynolate anion.^[152]^ On storing at −30 °C, **85** gets converted into dimeric form, while **86** transforms into dimeric form while drying, washing, and crystallizing. The dimeric forms of cAAC‐stabilized boraphosphaketenes (**87** and **88**) were characterized by single‐crystal XRD (Scheme 37).

Recently, an unprecedented report by Watt et al. has shown that a series of heterobimetallic lanthanum‐copper(I) and lanthanum‐gold‐(I) complexes can be isolated, by taking advantage of insertion reactivity of the La−P primary phosphide bond, that is supported by cAAC ligands.^[153]^ cAACs ligands have also been utilized to stabilized higher members of group 13 such as gallium. Recently, Radius et al. stabilized gallium hydride and gallium chlorohydride with cAAC ligand.^[154]^

**Scheme 38:**
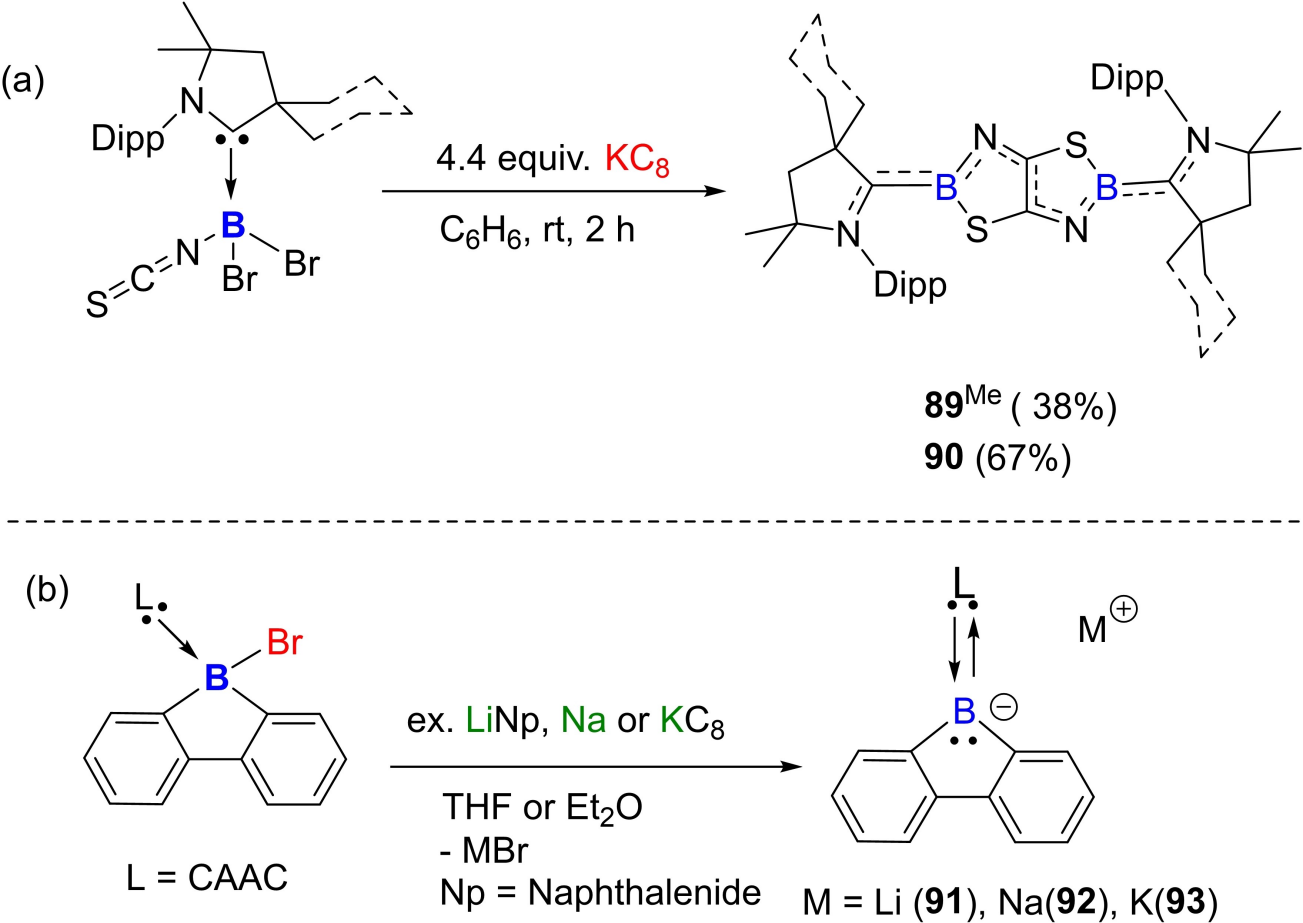
(a) Reductive dimerization of isothiocyanatoboranes in the absence of Lewis base (b) Synthesis of Lewis base‐stabilized borafluorene monoanions.

Braunschweig and coworkers have demonstrated excellent cAAC chemistry of boron as evident from their several recent reports. In one of the recent reports, they showed that the reduction of cAAC‐stabilized isothiocyanatoboranes in the presence of a Lewis base produces doubly base‐stabilized borylenes.^[155]^ However, when the reaction is carried out in the absence of base, the dimerization and C−C coupling of two (NCS) units produce [1,3,2]thiazaborolo[5,4‐d][1,3,2]thiazaboroles (Scheme 38). The bis(cAAC)‐stabilized thiazolothiazoles (**89** and **90**) that are obtained as a result of dimerization of borylene intermediate are deep blue in colour; however, their carbon analogues are colorless. In the subsequent work, they studied the reactivity of cAAC and NHC stabilized cyano‐ and isothiocyanatoborylenes for metal coordination, single‐electron oxidation, and basicity of boron center in these compounds.^[156]^ Additionally, cAAC stabilized borafluorene anions have been isolated and characterized by single‐crystal XRD. These compounds represent the first examples of elusive 9‐carbene‐9‐borafluorene monoanion (**91**, **92**, and **93**).^[157]^ These borafluorene anions react with transition metal and main group elements halides to produce tetracoordinate boron compounds. This reactivity is observed due to the nucleophilic character of borafluorene anions.

### 3.6 cAAC Chemistry of Group 14 elements

**Scheme 39:**
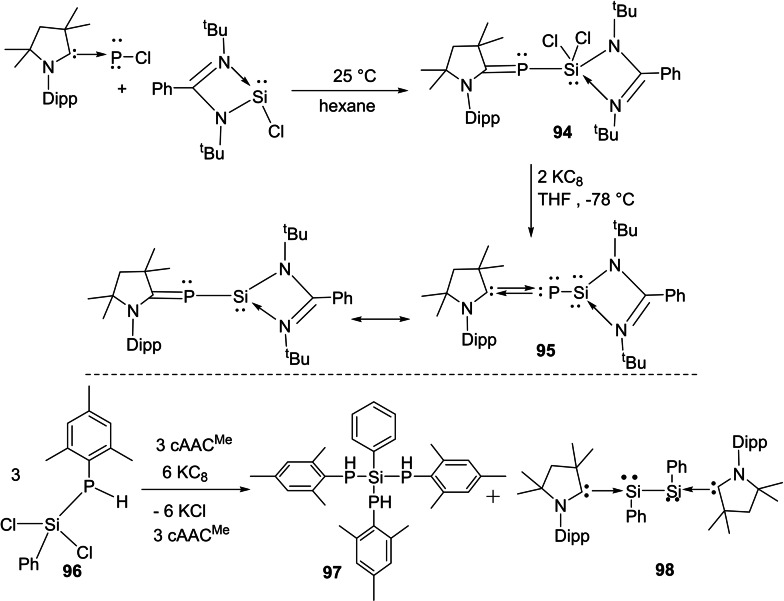
Synthesis of silylene‐phosphinidene.

The cAAC‐chemistry of carbon radicals/radicaloids and silicon has been covered in the recent reviews by Bertrand et al.^[15]^ and Roesky et al. and cAAC‐chemistry of silicon has also been the subject of several reviews.[^158^, ^159^, ^160^] Moreover, carbene stabilized exceptional silicon halides with unusual bonding have been discussed in a recent review.^[158]^ Therefore, in this section, only recent results will be discussed. Most recently, silylene‐phosphinidene (**95**), an interesting compound with a silylene‐phosphorus bond stabilized by cAACs has been reported by our group.^[161]^ For synthesizing this compound, heteroleptic chloro silylene (LSiCl) and chlorophosphinidene (Me−cAAC:→PCl) were reacted in toluene at room temperature and oxidative addition at silylene center gave pale yellow crystals of the product (**95**) in good yield. Further, compound (**94**) was reduced by two equivalents of KC_8_ in THF to produce cAAC‐anchored silylene‐phosphinidene L−Si−P(:cAAC−Me) (**95**). Yellowish orange crystals of (**95**) were isolated from hexane solution at −30 °C with 67% yield (Scheme 39, top).^[161]^ These compounds (**94** and **95**) are stable in solution and solid‐state under inert conditions. It has been calculated that cAAC‐phosphorus bond in compound (**95**) can equally well be described with two mutual dative bonds or electron sharing double bond. Additionally, another cAAC‐anchored silylene complex with two terminal phosphinidene moieties was reported by Frenking and co‐workers in the year 2018.^[162]^ Liu et al. synthesized a silicon‐phosphorus compound from the reaction of MesPH_2_ sequentially with nBuLi and PhSiCl_3_ in a 1 : 1 : 1 ratio.^[163]^ Furthermore, reduction of MesP(H)SiCl_2_Ph (**96**) with KC_8_ in the presence of cAAC gives (MesPH)_3_SiPh (**97**) and cAAC‐stabilized Si_2_Ph_2_ (**98**) (Scheme 39, bottom).

**Scheme 40:**
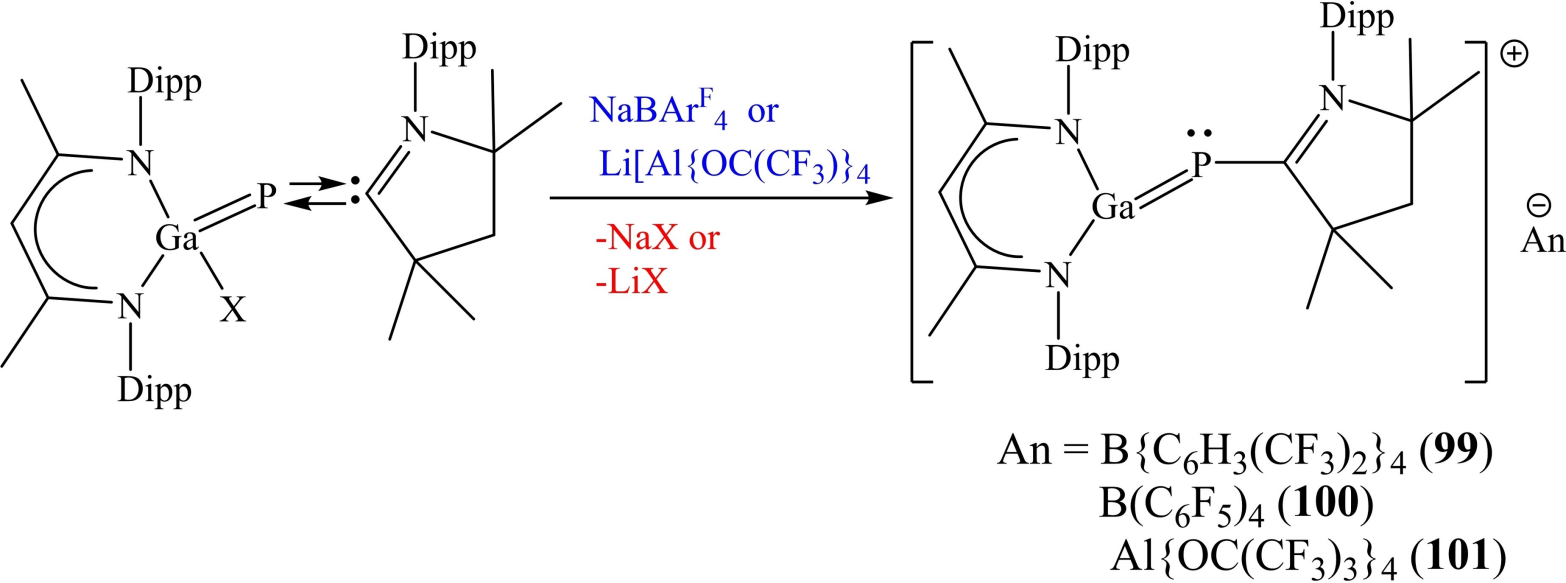
Synthesis of phosphinidenes.

It has been demonstrated in several reviews^[15]^ that cAAC chemistry of group 14 elements is dominated by silicon. Several cAAC stabilized silicon complexes in which silicon has a low or even zero oxidation state, have been reported. However, higher congeners of silicon have been rarely stabilized. Recently, Bertrand et al. have been successful in incorporating cAAC−Cu^I^ complex into the Ge_9_ Zintl clusters.^[164]^ Li et al. demonstrated that the oxidative addition of cAAC‐coordinated phosphinidenes affords gallium‐coordinated phosphinidenes LGa(X)‐P(^Me^cAAC) which further produce heteronuclear congeners, [LGaP(^Me^cAAC)][An] (An=B(C_6_H_3_(CF_3_)_2_)_4_ **99**, B(C_6_F_5_)_4_ **100**, Al(OC(CF_3_)_3_)_4_ **101**, of allyl cation on halide abstraction reaction.^[165]^ In these compounds, Ga center possesses a strong electrophilic character representing allylic nature (Scheme 40).

**Scheme 41:**
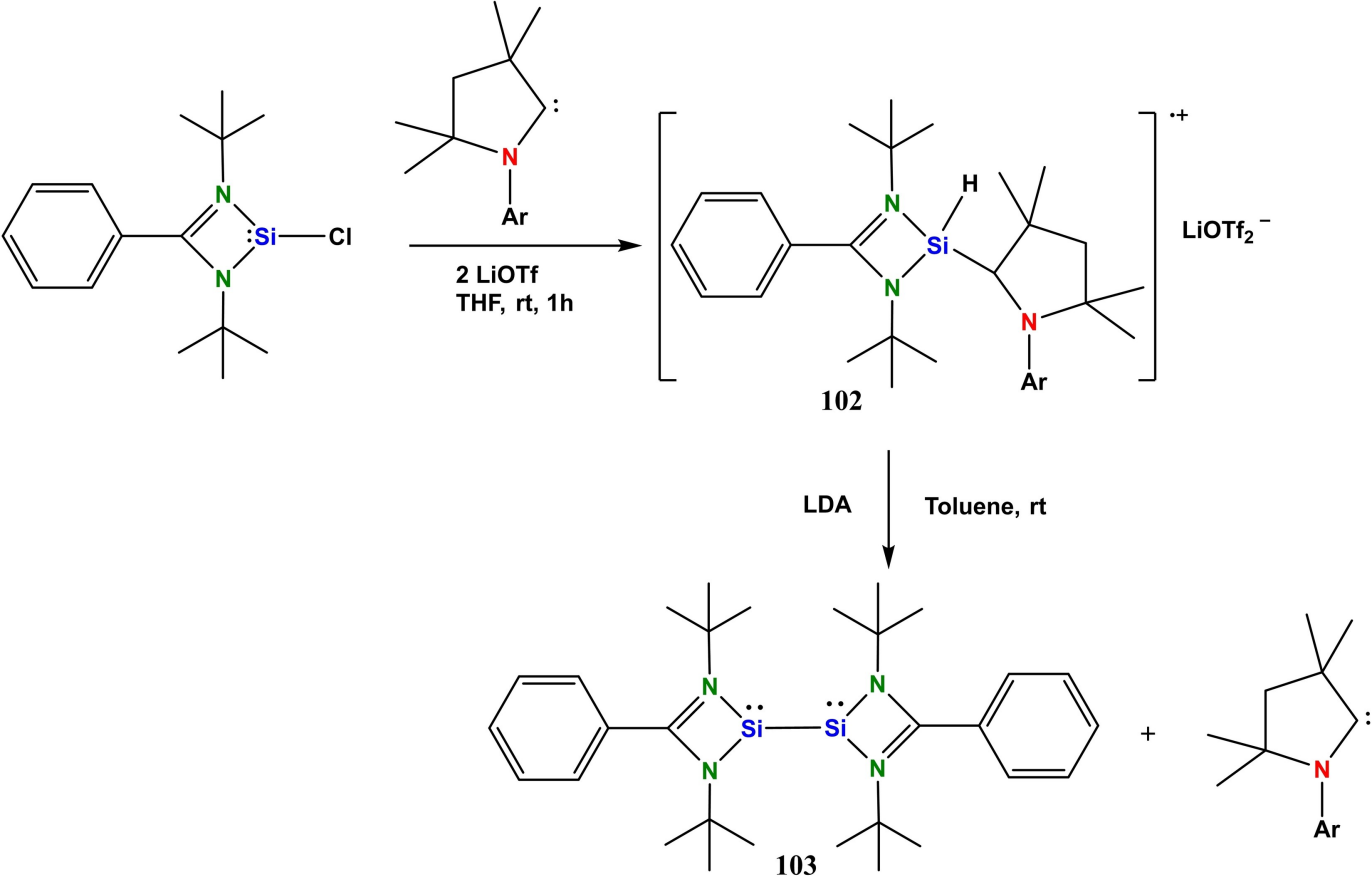
Synthesis of cAAC‐silyl radical.

The stabilization of radical species of boron has been highly explored, however, its higher congener germanium is still unexplored due to synthetic challenges, as its radical species are highly prone to undergo dimerization. Encouragingly, Li et al. reported the formation of cAAc‐silanyl radical that is stable up to 1 h at rt and decomposes at 141 °C. When amidinatosilylene LSi(:)Cl was treated with cAAC in THF in the presence of LiOTf, cAAc‐silanyl radical (**102**) was obtained, and possibly the hydrogen was abstracted from the THF (Si−H_(IR)_ 2247 cm^−1^) because the same reaction in toluene and Et_2_O does not give the product (**102**). The compound **102** on treatment with LDA in toluene produces bis‐silylene (**103**) and free cAAC ligand (Scheme 41).^[166]^

**Scheme 42:**
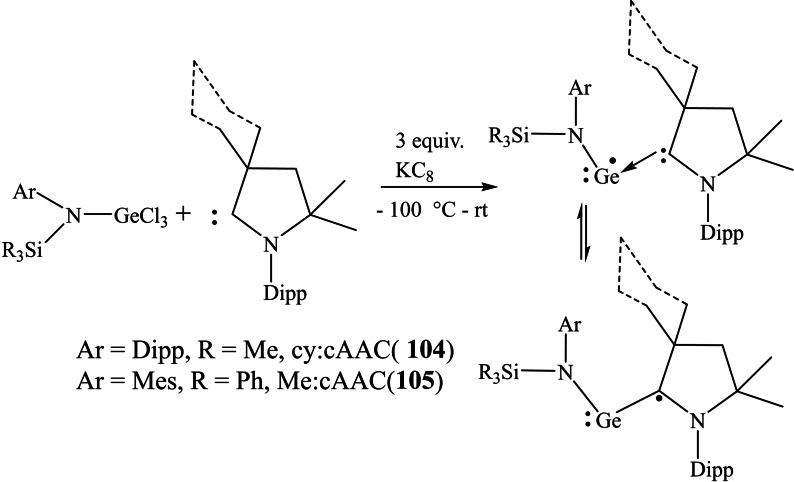
Synthesis of **104** and **105**.

We have recently isolated transient acyclic germanium(I) radicals stabilized by cAACs.^[167]^ The dark purple color crystals of Cy−cAAC : GeN(SiMe_3_)Dipp (**104**) and Me−cAAC : GeN(SiPh_3_)‐Mes (**105**) were isolated with 55 and 63% yield at −30 °C (Scheme 42). These compounds are stable at room temperature and can stay for even months in hexane at −30 °C, but lose color immediately on exposure to the air. The EPR spectra were recorded to confirm the radical nature of these species.

**Figure 7:**
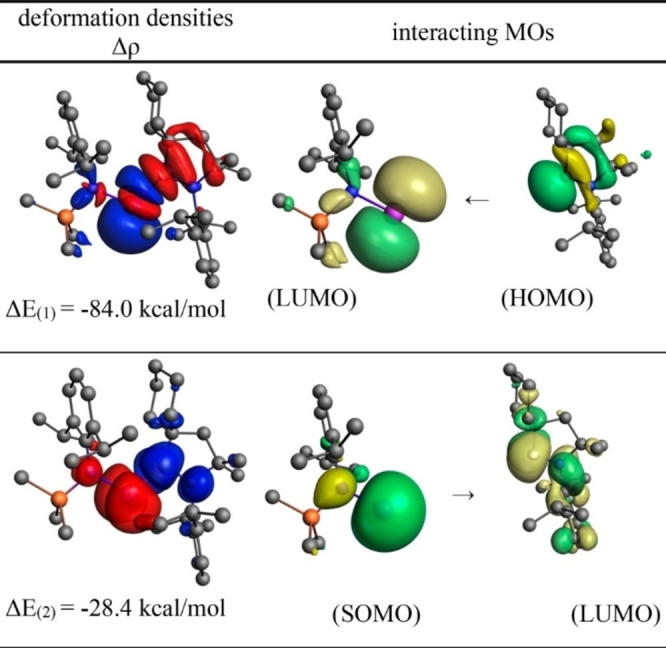
Shape of the most important interacting MOs of fragments of **104**, plot of deformation densities Δρ of the pairwise orbital interactions and the associated interaction energies (Δ*E*). The direction of the charge flow is red→blue. The figure reproduced with permission from Ref. [167]. Copyright (2018) American Chemical Society.

The quantum chemical calculation using density functional theory (DFT) was performed at the UB3LYP/6‐31G** level to understand electronic transitions in **104** and **105**. The theoretically calculated spectra essentially match with experimentally observed bands in the range of 249–555 nm. The Ge−cAAC bond was analyzed by performing energy decomposition analysis (EDA) coupled with natural orbital for chemical valence (NOCV). The EDA‐NOCV analysis calculations at the BP86‐D3(B)J/TZ2P level of theory reveal that there are two major covalent orbital interactions with more than 80% orbital interaction. The strongest interactions which come from the donation of lone pair of electrons from cAAC to vacant orbital of germanium are evident from observation of deformation densities (Δρ) and respective interacting orbitals (Figure 7).

Nowadays, EDA‐NOCV is prominently being used to study the nature of chemical bonds in molecules including bonding in cAAC compounds, therefore, here we would like to add a brief discussion about it. The fundamental principle of EDA involves the decomposition of intrinsic interaction energy (Δ E_int_) into four energy components as shown in Equation (1). The sum of these energy components is manifested in the form of experimentally observable bond dissociation energy.[^168^, ^169^]

(1) $$\Delta E{}_{\mathrm{int}=}\Delta E{}_{\mathrm{elstat}}+\Delta E{}_{\mathrm{Pauli}}+\Delta E{}_{\mathrm{orb}}+\Delta E{}_{\mathrm{disp}}\;$$

Where the term Δ *E*_elstat_ is related to the quasiclassical electrostatic interaction between the unperturbed charge distributions of the prepared fragments, Δ E_Pauli_ represents Pauli repulsions, which are the destabilizing interactions between occupied orbitals of the fragments. The term Δ *E*_orb_ represents orbital interaction, which accounts for three factors; the mixing of orbitals, charge transfer, and polarization. The term Δ *E*_disp_ accounts for the dispersion interaction energy between two interacting fragments. The combination of EDA with NOCV gives rise to the EDA‐NOCV method that involves the combination of charge and energy partition scheme, which provides a quantitative manifestation of the chemical bond in terms of physically significant contributions. Hence it helps in the understanding of the complete picture of the bonding situation in a molecule. The charge deformation Δρ_k_(r), which is obtained as a consequence of the mixing of the orbital pairs ϕ_k_(r) and ϕ_‐k_(r) of the interacting fragments gives the amount and the shape of the charge flow due to the orbital interactions [Equation (2)], and the associated energy term Δ E_orb_ provides the size of stabilizing orbital energy originating from such interactions [Equation 3].

(2) $$\Delta \rho k\left(r\right)=\sum_{k}\Delta \nu_{k}\left(r\right)=\sum_{k=1}^{N/2}{\nu_{k}[-\phi }^{2}-\psi_{-k}^{2}\left(r\right)+\psi_{k}^{2}\left(r\right)]$$

(3) $${\Delta E}_{Orb}=\sum_{k}{\Delta E}_{Orb}^{k}=\sum_{k}\nu_{k}\left[-F_{-k,-k}^{TS}+F_{k,k}^{TS}\right]$$

Recently, we have employed EDA‐NOCV to study the nature of chemical bonds in several cAAC stabilized species.[^170^, ^171^, ^172^] Carbon, being the most important element of group 14, has been stabilized by cAAC ligands in different cluster forms. For example, EDA‐NOCV reveals that the cAAC stabilized linear C_2_ ^[170]^ and C_3_ ^[171]^ possess electron sharing double bond in electronic quintet state, between cAAC and C_2_/C_3_ unit, forming a cumulene. However, C_2_ and C_3_ stabilized by NHC form electron sharing and dative bonds between two fragments.

### 3.7 cAACs Stabilized Interstellar Species

**Scheme 43:**
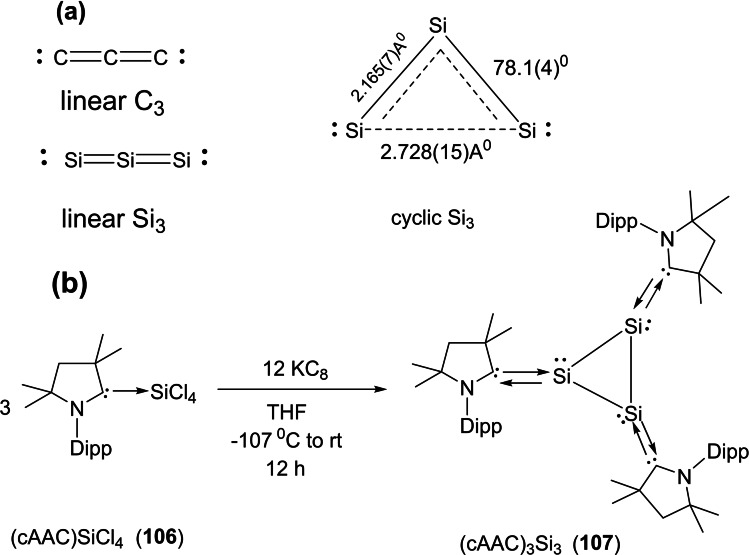
**(a)** Linear and cyclic isomers of C_3_ and Si_3_ (**b**) Synthesis of compound **107**.

Interstellar species are unusual molecules that are formed by a chemical reaction within very sparse interstellar or circumstellar clouds of dust and gas. It is important to note that dust plays an important role in protecting molecules from the ionizing effects of ultraviolet radiation emitted by stars.^[173]^ The interstellar space (filled with ultra low‐density gases) is the unique laboratory that allows the formation of unusual species which are not possible to synthesize in normal conditions on the earth. Therefore, exploration of these species has been a curious endeavor of chemists due to their unusual properties. Consequently, over 180 interstellar and circumstellar species have been detected and characterized by spectroscopic methods.^[173]^ The lighter group 14 elements play an important role in space astrochemistry. Recently, several interstellar species such as C_3_, C_5_, Si_3,_ SiC_2_, SiC_3_, SiCN, and SiNC in space[^174^, ^175^] have been reported. Cyclopropenylidene (C_3_H_2_) is a cyclic singlet carbene with reports of its radio astronomical detection in 1985.^[176]^ This species has been considered to be the most abundant cyclic hydrocarbon detected in interstellar space and it is detectable in molecular clouds, circumstellar shells, and also in one external galaxy.^[177]^ The Si_3_ cluster is suspected to be a suspected interstellar species and recently has been stabilized. Interestingly, the serendipitous discovery of Si_3_ was reported while characterizing a new silicon hydride. The structure and bonding in silicon clusters are significantly different than those of their lighter analogues.[^178^, ^179^] It is found that linear C_3_ is more stable than cyclic C_3._ In contrast, bent Si_3_ is more stable than its linear Si_3_ form by 9.5 kcal mol^−1^ (Scheme 43, a).[^180^, ^181^] The electronic and optical properties of silicon‐containing semiconductors depend upon the microscopic molecular structure as well as bulk composition; therefore it becomes important to study clusters of silicon[^182^, ^183^] and their interactions with different types of ligands. Mono‐ and diatomic silicon (0) has been stabilized by NHCs and cAACs^[184]^ due to stronger σ‐donor and better π‐acceptor properties of cAACs. Taking advantage of this property of cAACs, We synthesized triatomic silicon(0) compound, (cAAC)_3_Si_3_ (**107**) by using cAAC stabilized (cAAC)SiCl_4_ (**106**) as precursor (Scheme 43 (b)).^[185]^

Furthermore, this triatomic silicon (0) compound (**107**) is stable at room temperature; therefore, it can easily be isolated and stored. The structural properties were studied using single‐crystal X‐ray determination with synchrotron radiation^[185]^ and aspherical‐atom least‐squares refinement.^[186]^ The structural analysis reveals that triangular Si_3_ is sterically well crowded with three cAACs ligands and no cAAC ligand is in the plane of the Si_3_ unit. The Si−Si bond distance in the compound (**107**) is 2.399(8) A, 2.369(8) Å and 2.398(8) Å which are longer by about 0.04 Å than the sum of Si covalent radii (2.34 Å).^[187]^ Each Si atom has formal oxidation state zero and Si_3_ adopts a three‐coordinate trigonal pyramidal shape.^[185]^ ^29^Si NMR of **107** in C_6_D_6_ gives a singlet at 7.20 ppm that is downfield‐shifted compared to its precursor. However, this ^29^Si NMR resonance of **107** is upfield shifted when it is compared to that of the corresponding monoatomic (+66.71 ppm) and diatomic (+254.60 ppm) cAAC stabilized Si(0) compounds. The computational calculations were performed using density functional theory at BP86/def2‐SVP level to study the bonding situations in **107**. The calculated bond angles and bond distances are in excellent agreement with experimentally determined values. The natural bond orbital (NBO) analysis shows that the Si_3_ moiety has three σ ‐bonds orbitals which are slightly polar due to the asymmetry of Si(cAAC) units. The three cAAC→ Si dative bonds show three σ ‐donor bond orbitals that are polarized with 68–71% toward the carbon atom. The p‐type bond orbitals that originate from the cAAC← Si π‐back donation are almost nonpolar with large π‐back‐donation. The large back donation can be due to the strong repulsion between three lone pair orbitals which experience exchange repulsion. We^[188]^ synthesized another interesting compound of germanium supported by cAAC. In the reaction, MeGeCl_3_ was reduced by three equivalents of KC_8_ in the presence of one equivalent that gave rise to (cAAC)MeGe‐GeMe(cAAC).^[188]^ The silicon analogue of this compound was also synthesized by the same method taking MeSiCl_3_ instead of MeGeCl_3._ Readers are requested to refer to a review by Frenking et al. for the insightful account of the nature of bonding in cAAC stabilized group 14 elements.^[189]^

### 3.8 cAAC Stabilized Group 15 elements

**Scheme 44:**
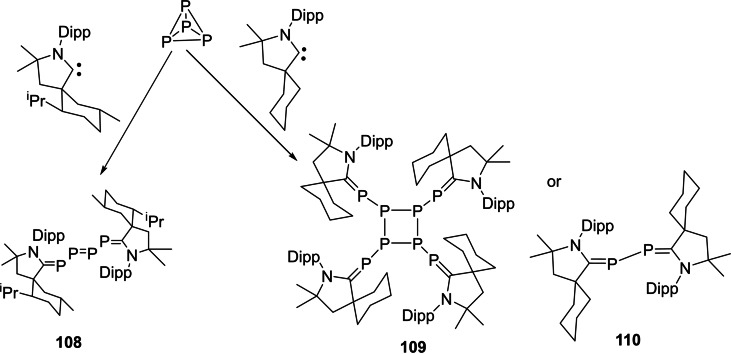
Singlet carbenes as activator for white phosphorus.

The stabilization and activation of small reactive molecules have always been fascinating to the chemists and group 15 elements have provided much impetus to this idea. For example, cAAC chemistry of P_4_ is one of many such applications. It is evident from recent publications in the area of stable carbenes that among various applications of cAACs, activation of small molecules especially P_4_ has got enough attention in carbene chemistry. It is well established that transition metals are excellent species to activate and stabilize small molecules. Moreover, reactions of P_4_ with transition metals have been widely studied. Therefore, researchers became interested in studying the reactions of P_4_ with cAACs in the same manner as those of transition metals. Surprisingly, cAACs showed that they can activate white phosphorus by opening the tetrahedron, fragment, and even aggregate formation resulting in P_4_, P_1_, P_2_, and P_12_ carbene‐stabilized species (Scheme 44).^[190]^ Depending upon experimental conditions and steric crowding of substituents on cAAC, different phosphorus derivatives are obtained. When P_4_ reacts with methyl‐substituted cAAC, P_4_ undergoes cage opening resulting into a 2,3,4,5‐tetraphosphatriene derivative (**108**).^[191]^ Similarly, cyclohexyl substituted cAAC reacts with P_4_ to give either P_8_ tetracarbene (**109**)^[190]^ or P_2_ dicarbene adduct (**110**).^[192]^ Interestingly, 2, 3, 4, 5‐tetraphosphatriene analogue of **108** undergoes dimerization to give (**109**). However, **110** is obtained on the attack of a second cAAC molecule on **108**. Notably, all these stoichiometric reactions give different phosphorus‐carbene adducts depending upon electronic and steric factors. However, except for stoichiometric reactions, activation and functionalization of P_4_ have not been possible by catalytic process, even with transition metals.^[193]^

**Scheme 45:**
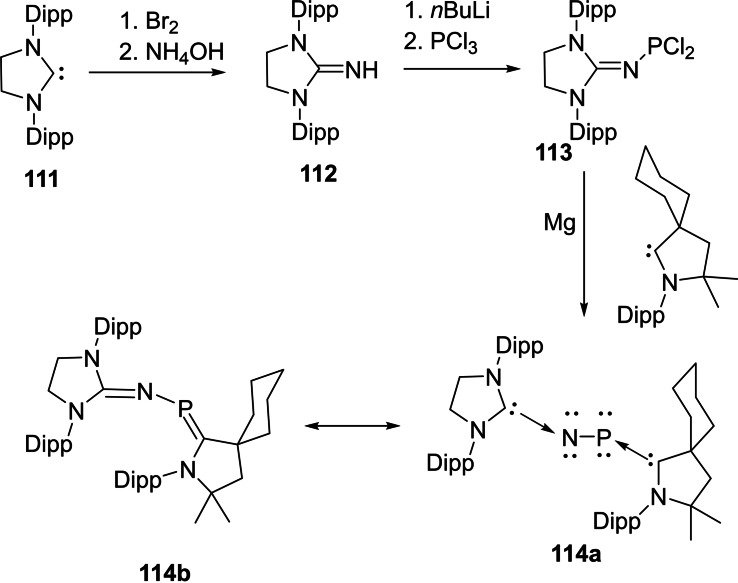
Synthesis of carbene stabilized phosphorus mononitride.

Considering the fact that cAACs act as elegant ligands for stabilizing small molecules; Bertrand's research group succeeded in synthesizing carbene ligated phosphorus mononitride.^[194]^ This carbene stabilized adduct was prepared by bromination of the NHC **111** followed by ammonolysis. The guanidine derivative **112** obtained in the reaction further underwent deprotonation by nBuLi and quenching of lithium derivative with PCl_3_ gave compound **113**. Lastly, compound **113** formed an adduct with cAAC which subsequently gives the final product **114** after getting reduced by magnesium (Scheme 45).^[195]^ This compound **114** is indefinitely stable in air and does not undergo decomposition even on heating in toluene solution under reflux for 24 h.

**Scheme 46:**
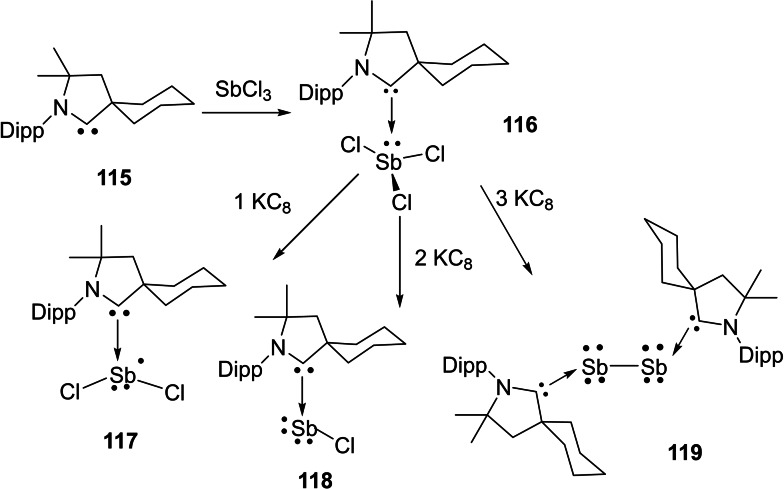
***c*** AAC stabilized antimony in different oxidation states.

Reactions with antimony exhibit that cAACs can stabilize atoms in various oxidation states in which sterically demanding ligands have played a central role.^[196]^ More recent studies have shown that a smaller HOMO‐LUMO gap in cAACs allows isolation of various species whereas NHCs cannot compete.[^22^, ^109^] Inspired by these results, Bertrand et al. synthesized cAAC−SbCl_3_ complex and studied stepwise reduction by one, two, and three electrons. The starting material **116** was freshly prepared by mixing SbCl_3_ in ether solution of cAAC **115** and a white solid adduct with 94% yield was obtained (Scheme 46).^[196]^ The reduction of **116** with one equivalent of KC_8_ in benzene produces golden color, NMR silent solution of **117**. The EPR study reveals the paramagnetic nature of compound **117**. The density functional theory (DFT) calculations of **117** reveal that the antimony center has a T‐shaped environment and spin density is almost exclusively located at antimony (90.7%). The addition of two equivalents of KC_8_ to compound **116** gives, after workup, NMR active yellow solid **118** with 26% yield. Compared to **116**, a downward shift in the carbene NMR resonance (241.3 ppm) was observed. Lastly, three electrons reduction of **116** was carried out by adding three equivalents of KC_8_, and deep purple colored product **119** was isolated with a 45% yield.^[196]^

**Scheme 47:**
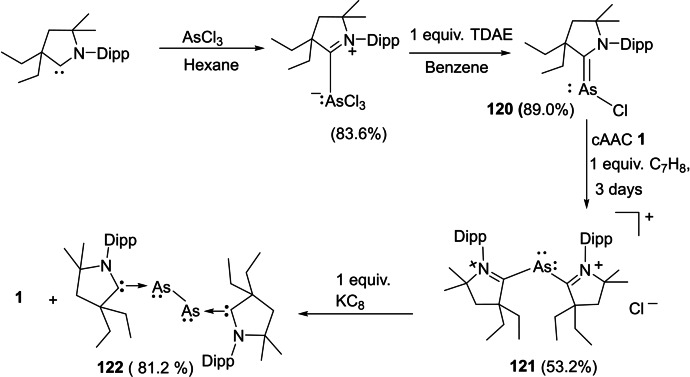
Synthesis of chloroarsenidene and diarsenic compound supported by cAACs.

Although, various group 15 elements have been stabilized by cAACs and their applications have also been explored but probably, cAAC‐supported arsenic compounds have not been synthesized. Most recently, Hudnall and coworkers have reported the first synthesis of cAACs‐supported chloroarsinidene **120** [194] featuring arsenic in a +1 formal oxidation state. They have also synthesized diarsenic allotrope **122** supported by two cAAC molecules. While endeavoring for the synthesis of cAAC supported diarsenic, they serendipitously discovered arsamethine cyanine dye **121**. The discovery of **121** was striking since analogous cyanine dyes are reported to have diverse applications^[197]^ including biological labeling, photovoltaics, electronics, textile, imaging, etc. Compound **121** was found as an intermediate that on reduction with 1 equivalent of KC_8_ in benzene gave rise to compound **122** (Scheme 47).^[198]^

**Scheme 48:**
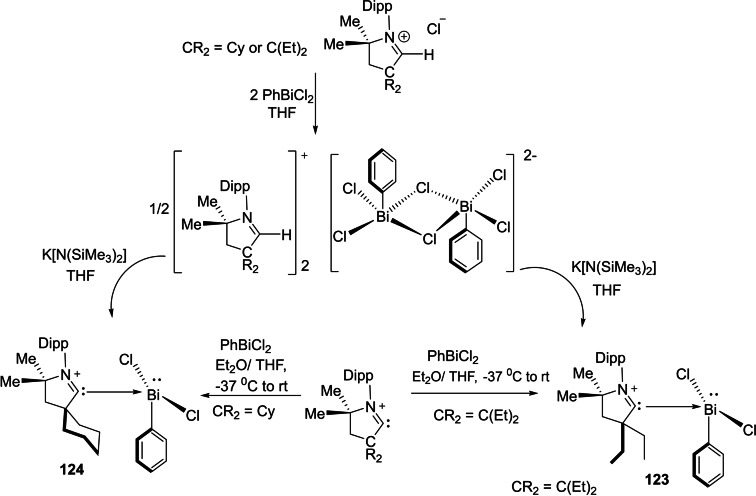
Synthesis of cAAC stabilized bismuth complexes **123** and **124**.

**Figure 8:**
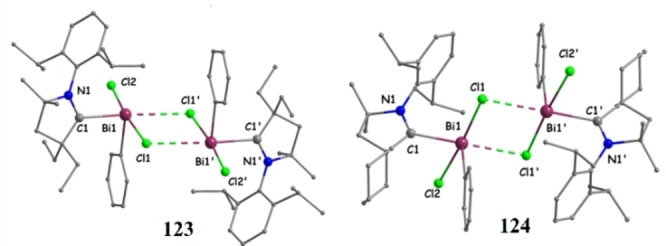
Dimers of **123** and **124** (crystal structure taken from CCDC deposition by Ref. [200]. Copyright (2018) American Chemical Society.

The cAAC chemistry of group 15 elements is dominated by phosphorus stabilized cAAC complexes which have been isolated in high yields. One such example has been reported by our group, where we have synthesized chlorophosphinidene,^[199]^ (cAAC)P−Cl. This cAAC stabilized chlorophosphinidene can be synthesized directly from reacting cAAC and phosphorus trichloride (PCl_3_).^[199]^ However, analogous cAAC complexes of heavier group 15 metals are very less reported. Recently, Gilliard et al.^[200]^ reported cAAC stabilized bismuth complexes (**123** and **124**) which were synthesized in an inert atmosphere by reacting phenylbismuth dichloride (PhBiCl_2_) and cAAC in THF/toluene mixture (Scheme 48). The bismuth stabilized cAAC complexes **123** and **124** were isolated in 45 and 50% yields, respectively. The single‐crystal X‐ray diffraction study reveals that the asymmetric unit of **123** exhibits tetracoordinate bismuth in a seesaw environment with two Bi−Cl and two Bi−C bonds. The packing diagram reveals that an additional Bi−Cl interaction stabilizes **123** as a dimer having pentacoordinate bismuth with square pyramidal geometry. Similar to the **123**, complex **124** also exists as a dimer in solid‐state (Figure 8). Noteworthy is the fact that **123** and **124** can also be obtained by deprotonation of an intermediate that is synthesized by reacting [Et_2_cAAC−H]^+^[Cl]^−^ and [cy−cAAC−H]^+^[Cl]^−^ salts with phenylbismuth dichloride. However, the direct reaction of phenylbismuth dichloride with cAAC gives easily isolable pure complexes. Braunschweig et al. in a recent investigation have shown that cAACs can undergo a Staudinger‐type reaction with trimethylsilylazide (TMSN_3_).^[201]^ The resulting compound (^Me^cAAC=NSiMe_3_) has shown to be an excellent agent for ^Me^cAAC=N^−^ transfer onto the transition metal and main group elements.

**Scheme 49:**
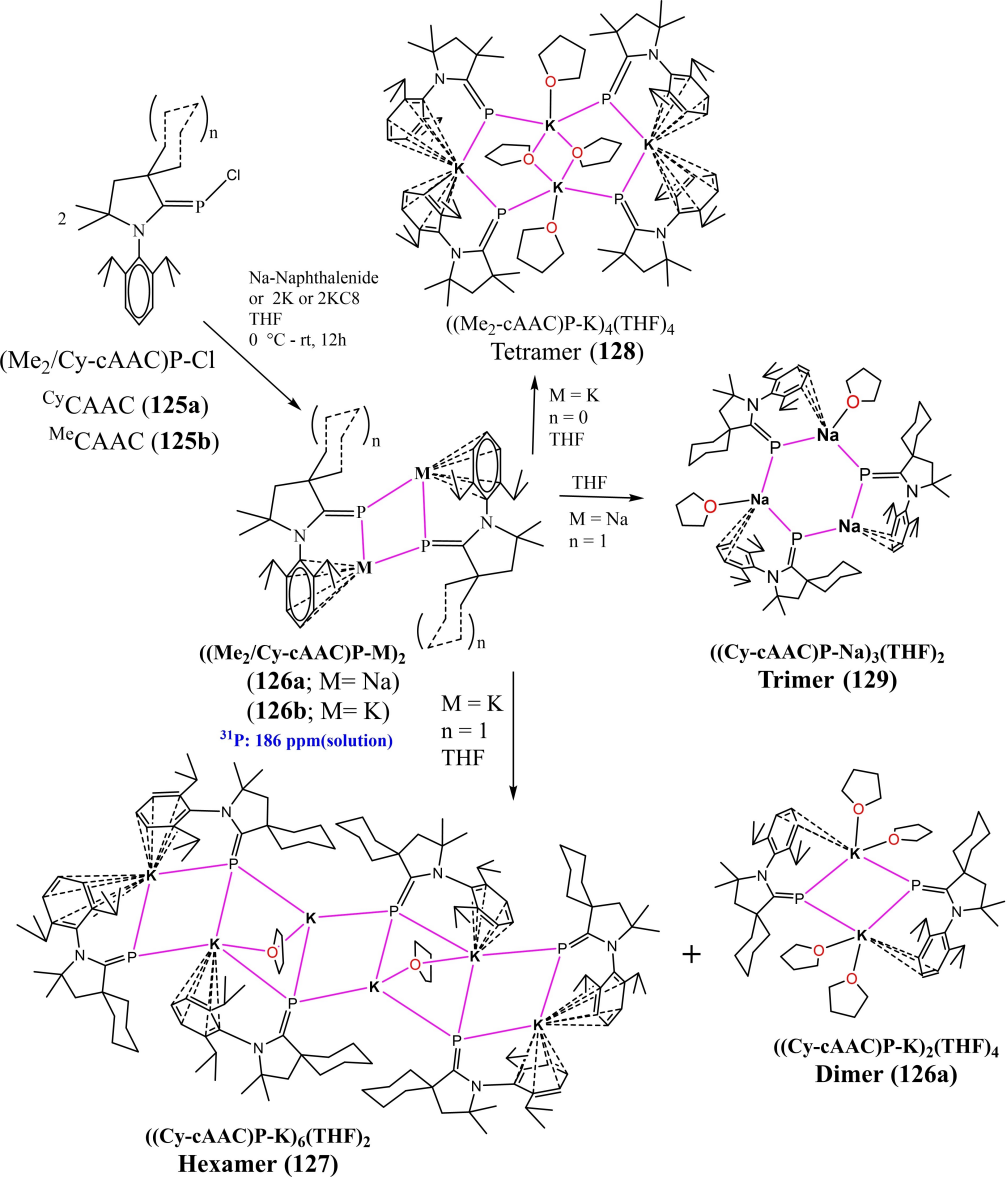
Synthesis of cAACs‐supported metal‐phosphinidenides.

Recently, Roy and Mondal et al. reported^[202]^ alkali metal‐mediated isolation of cAAC‐supported oligomeric forms of sodium/potassium‐Phosphinidenides with general formula ((cAAC)P−M)_n_(THF)_x_ where M=Na/K, n=2, 3, 4, 6 and x=1, 2, 4. The alkali metal‐Phosphinidenides were synthesized by the reduction of cAAC‐chlorophosphinidenes (**125 a**), using K/KC_8_/Na‐naphthalenide as reducing agents (Scheme 49). The cAAC‐supported chlorophosphinidenes(cy−cAAC=P−Cl or Me_2_−cAAC=P−Cl) were reacted with suitable reducing agents as shown in Scheme 49, to get different oligomeric forms. The reaction of cy−cAAC=P−Cl with 2 K or 2KC_8_ in THF solvent between 0 °C–rt gives dark red crystals of dimeric((cy−cAAC)P−K)_2_(THF)_4_) (**126 a**) and hexameric potassium‐phosphinidenides ((cy−cAAC)P−K)_3_(THF)_2_) (**127**). In similar reaction conditions, **125 b** produces tetrameric complex **128**. The reaction of Me_2_−cAAC=P−Cl with freshly prepared Na‐naphthalenide gives trimeric sodium‐Phosphinidenide ((cy−cAAC)P−Na)_3_(THF)_2_) (**129**) at room temperature. However, at 0 °C, orange‐red crystals of hexameric sodium‐phosphinidenide were isolated from concentrated THF solution. Different oligomers obtained were characterized in solution phase using ^31^P NMR and in solid‐state using single‐crystal X‐ray diffraction.

**Figure 9:**
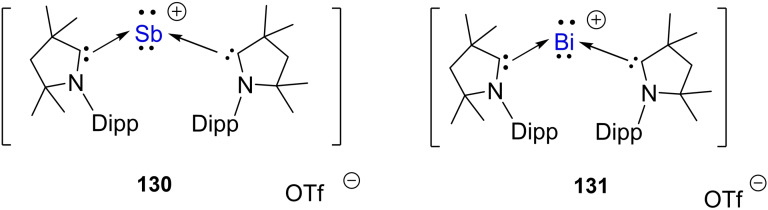
cAAC stabilized Sb(I) and Bi(I) complexes.

**Figure 10:**
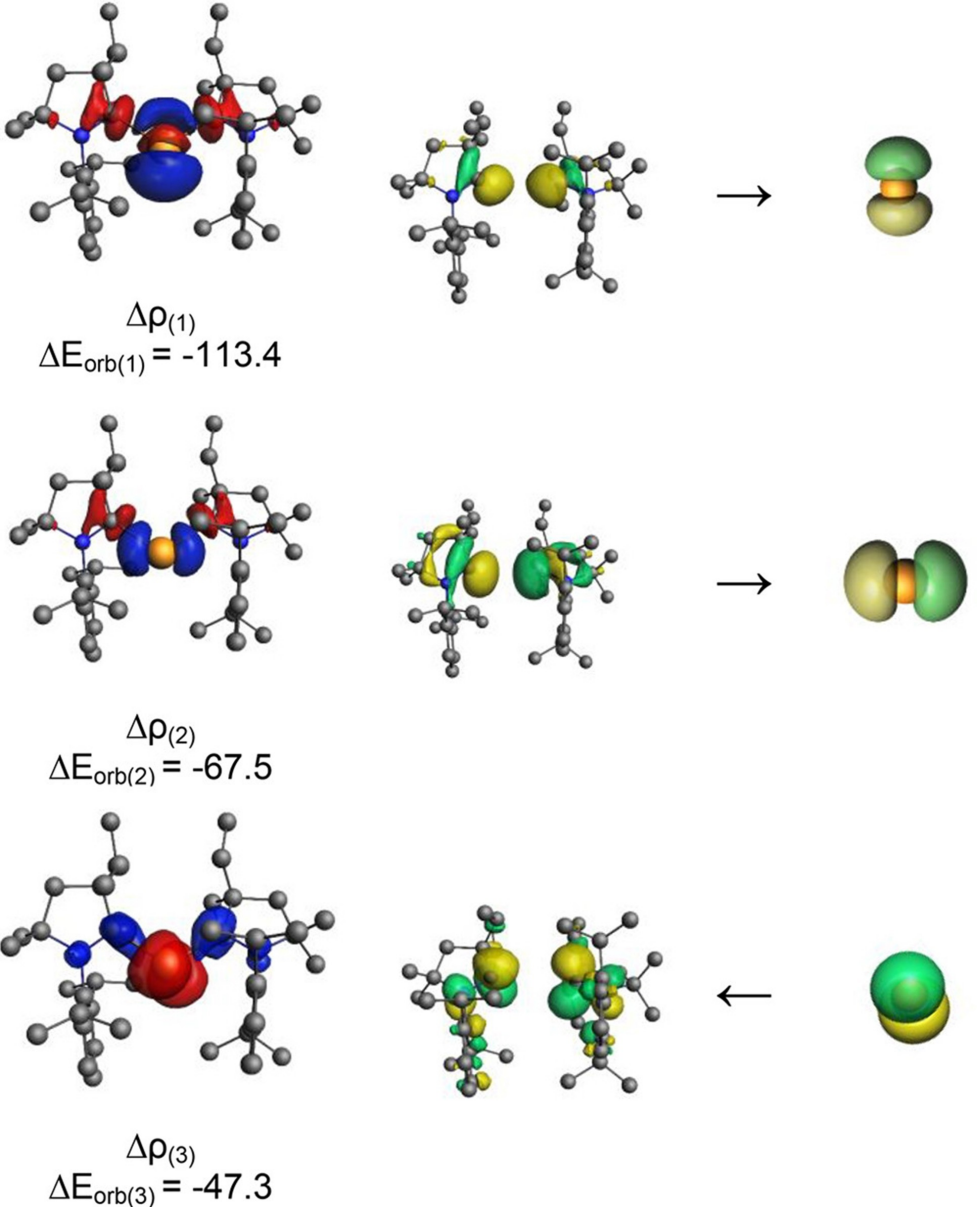
Plot of the deformation densities Δρ of the pairwise orbital interactions between the two fragments in their singlet electronic state in [Sb(CAAc)_2_]^+^. The energies are given in kcal/mol and the direction of the charge flow is red→blue. Reproduced with permission from Ref. [203]. Copyright (2021) American Chemical Society.

The isolation of compounds with two coordinate elements of group 15 is challenging due to the formation of dimers and oligomers as evident by the previous discussion. However, using a suitable cAAC ligand with carefully tuned reaction conditions, the donor stabilized two coordinate complexes of the heavier group 15 elements can be stabilized in lower oxidation states. For example, Siddiqui et al. have reported two coordinate Sb(I) and Bi(I) stabilized by cAAC.^[203]^ These complexes are synthesized by reduction of antimony and bismuth trihalides with KC_8_ in the presence of cAAC affording Sb(I) and Bi(I) cations in the form of triflate salts [(cAAC)_2_Sb][OTf] (**130**) and [(cAAC)_2_Bi][OTf] (**131**). These compounds represent a new class of acyclic cations of low valent group 15 elements which are analogs of carbones (Figure 9). The EDA‐NOCV calculations performed at BP86+(D3BJ)/TZ2P//BP86+(D3BJ)/def2‐TZVPP Level suggest that the pnictogen‐ligand bonds in the cations of **130** and **131** are best described in terms of σ donation and π back‐donation, as expected for heavier group 15 elements (Figure 10).

**Scheme 50:**
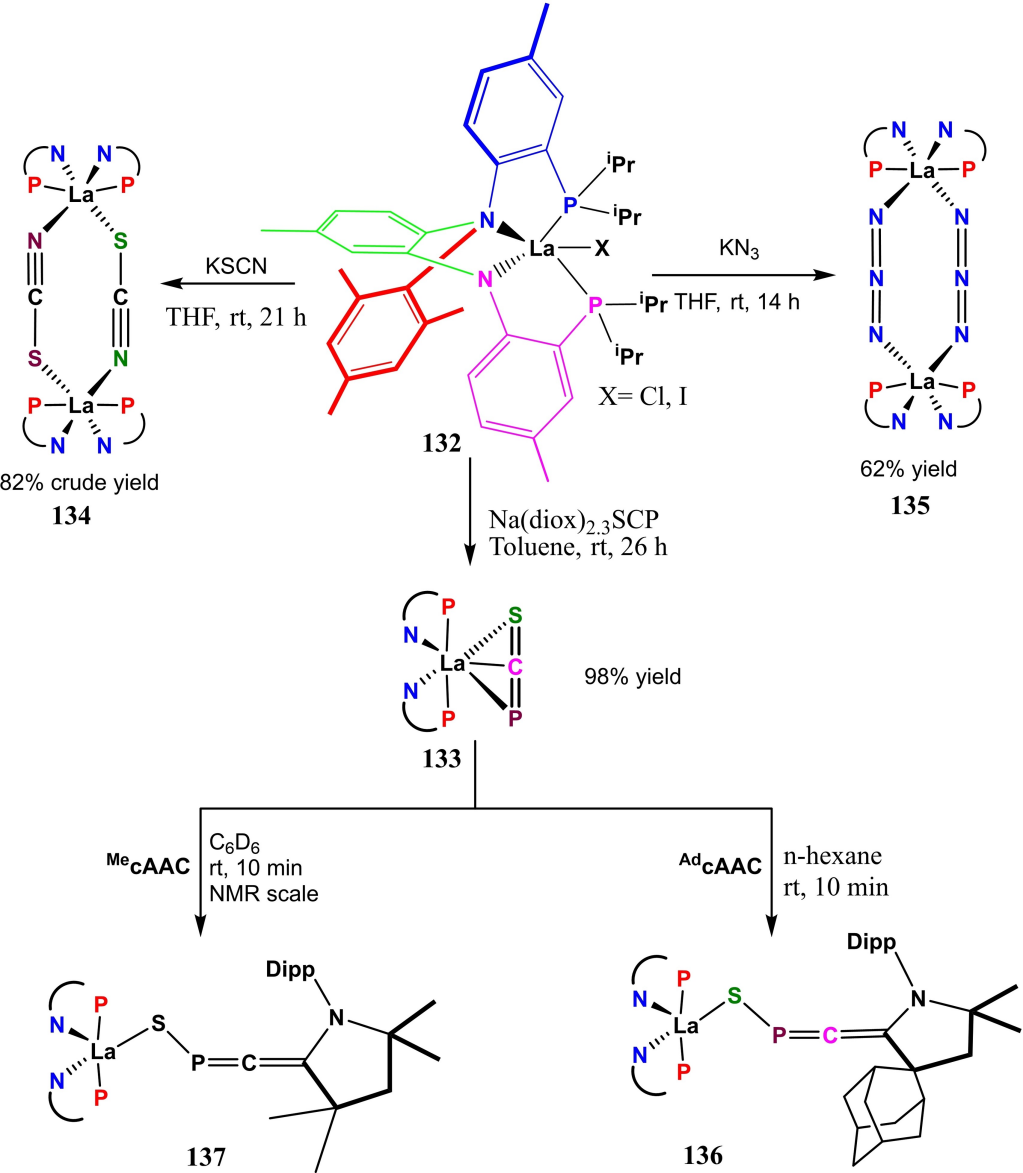
Synthesis and reactivity of lanthanum η ^3^‐coordination complex and its reactivity with cAAC ligands.

Hohloch et al. applied excellent Lewis acidic La salt (**132**) for stabilization of [SCP]^−^ ligand and surprisingly they found η ^3^‐coordination complex (**133**) in excellent yield while with [SCN]^−^ and [N_3_]^−^ salt metathesis products (**134** and **135**) were obtained (Scheme 50).^[204]^ The obtained η ^3^‐coordination product might be due to the strong Lewis acidic nature of La and electronic effect in [SCP]^−^ most of the negative charge density resides on the central carbon atom. It was also confirmed with single‐crystal XRD that the La−C bond length is shortest among all, La−S (3.036 Å), La−C (2.837 Å), La−P (3.343 Å). The product obtained completely depends on electronic factors but not on steric factors. Further, when the η ^3^‐coordination product was treated with strongly nucleophilic cAAC, a rearrangement product was obtained and [SCP]^−^ got rearranged to [SPC]^−^. Here, the authors found that the product formation is only governed by electronic effects, but not steric effects, because both ^Ad^cAAC product (**136**) and ^Me^cAAC product (**137**) were obtained in comparable yields.

### 3.9 cAAC Chemistry of Group 16 elements

**Scheme 51:**
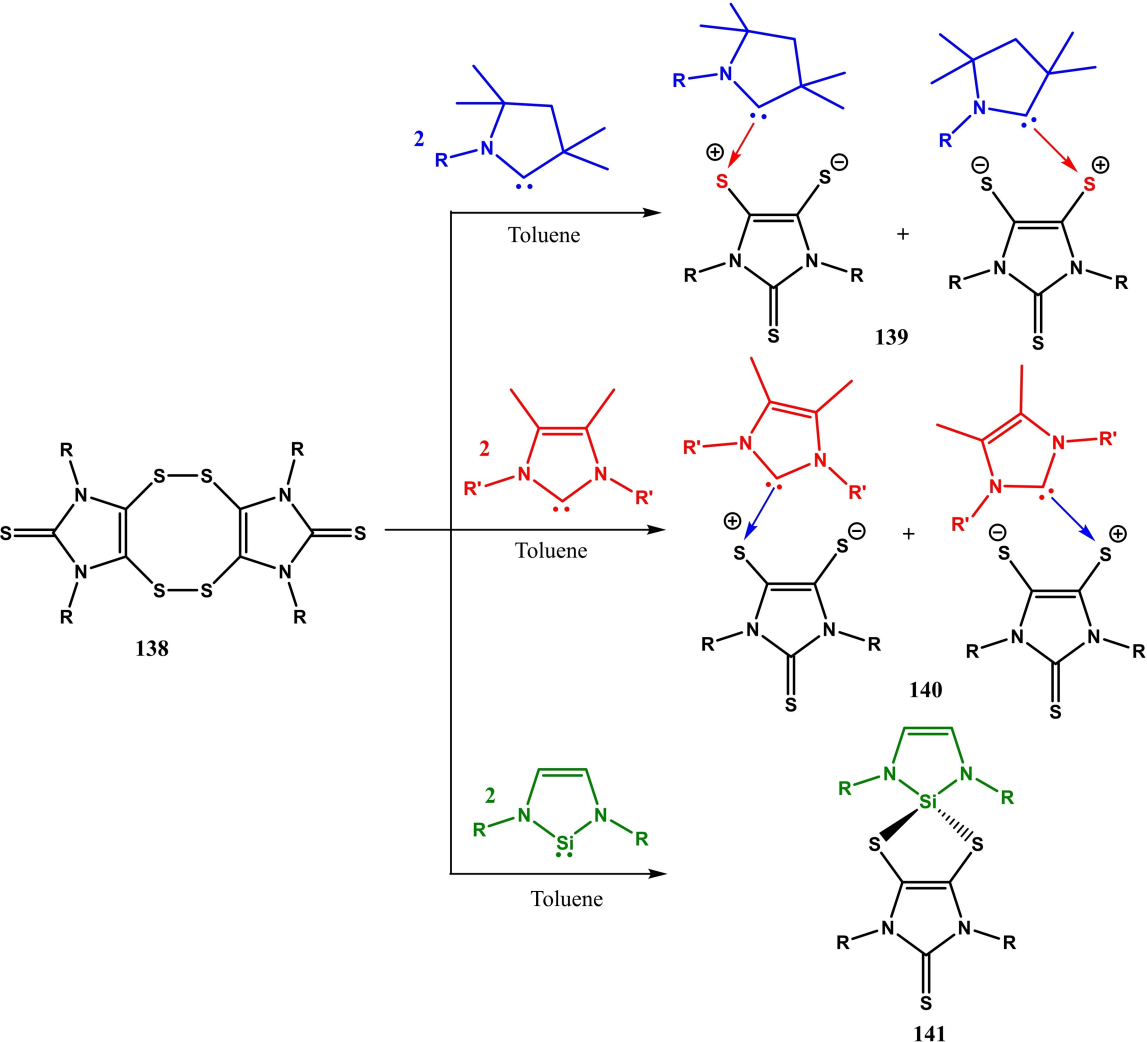
cAAC and NHC stabilized dithiolene and NHSi stabilized spiro dithiolene (R=2,6‐diisopropylphenyl).

The cAAC compounds of group 16 elements are limited. In a few cases, cAAC ligand have been employed to stabilize sulfur and selenium containing species. For example, cAACs have been utilized as ligands to stabilize neutral dithiolenes. Dithiolenes are unstable due to their interesting redox behavior originating from variable oxidation states. Due to variable oxidation states dithiolenes ligands exist in different forms such as dithiolate dianion (L^2−^), radical monoanion (L^.−^), and neutral dithione (or dithiete) (L^0^). In this regard, Wang et al. synthesized a imidazole‐based dithione dimer (**138**) and investigated its reaction with Lewis bases such as cAAC, NHC and NHSi.^[205]^ The reaction of neutral dithiolenes (L^0^) with cAAC and NHC ligands produces **139** and **140** as a result of S−S bond cleavage. However, the reaction of neutral dithiolene with NHSi produces a spirocyclic silicon‐dithiolene compound (**141**) (Scheme 51). These reactions represent a very important aspect of cAAC chemistry; since sulfur‐sulfur bond cleavage is vital to the biological processes and organic synthesis.

**Scheme 52:**
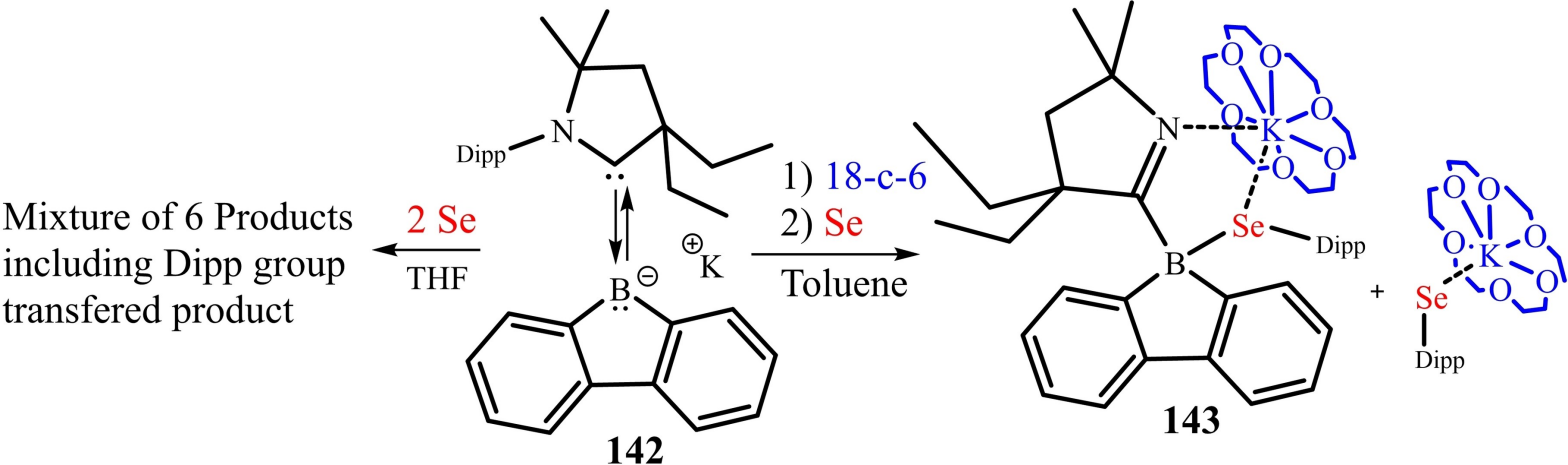
Reaction of 9‐cAAC‐9‐borafluorene monoanion with silicon grey powder.

The first boryl‐substituted selenides and diselenides were synthesized by Wilson et al. in 2021. When 9‐carbene‐9‐borafluorene monoanion (**142**) was treated with selenium grey powder, a mixture of boryl‐substituted selenides and diselenides including Dipp group transferred products were obtained.^[206]^ However, when the same reaction was performed in toluene with 18‐crown‐6, boron substituted monoselenide product (**143**) was obtained only (Scheme 52).

### 3.10 cAACs Stabilized Complexes in Medicine

**Figure 11:**
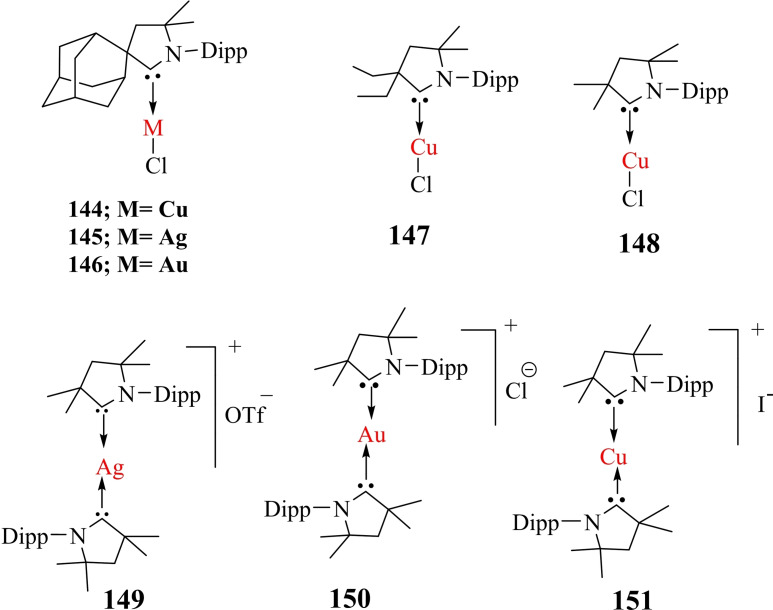
Selected cAAC‐complexes investigated for antiproliferative properties.

The use of platinum(II)‐complex, *cis*‐platin in the chemotherapy of various types of cancers has been a great success. However, application of cisplatin in chemotherapy has been associated with important drawbacks including severe normal tissue toxicity and resistance to treatment.^[207]^ Therefore, several other metal complexes have been tested for their cytotoxic properties. In this regard, gold(I/III) complexes have received considerable attention in recent years as potential anticancer agents.[^208^, ^209^] Recent studies reveal that gold(I/III) complexes with NHC and cAAC ligands have shown cytotoxic properties. Taking advantage of better electronic and steric properties, cAACs form, in comparison to NHCs, stronger bonds with the coinage metals, therefore they become ligands of a better choice. Bochmann and co‐workers studied antiproliferative properties of cAAC complexes of copper, silver, and gold. They considered cAAC complexes **144**–**151** (Figure 11)^[210]^ for this investigation and performed determination of the half‐maximal inhibitory concentration (IC50) value on a panel of human cancer cell lines such as Leukemia (HL 60), human lung adenocarcinoma epithelial cells (A549) and breast edenocarcinoma cells (MCF‐7). These complexes, in particular, were found to be active on HL 60 and MCF‐7 cell lines with the IC_50_ value in the range of micromolar to 100 nanomolar. Surprisingly, these complexes were found to be more active than cisplatin.^[210]^ Therefore, it is reasonable to be optimistic and consider that cAAC supported metal complexes could be potential applicants in cancer chemotherapy.

### 3.11 Luminescent cAAC Complexes

**Figure 12:**
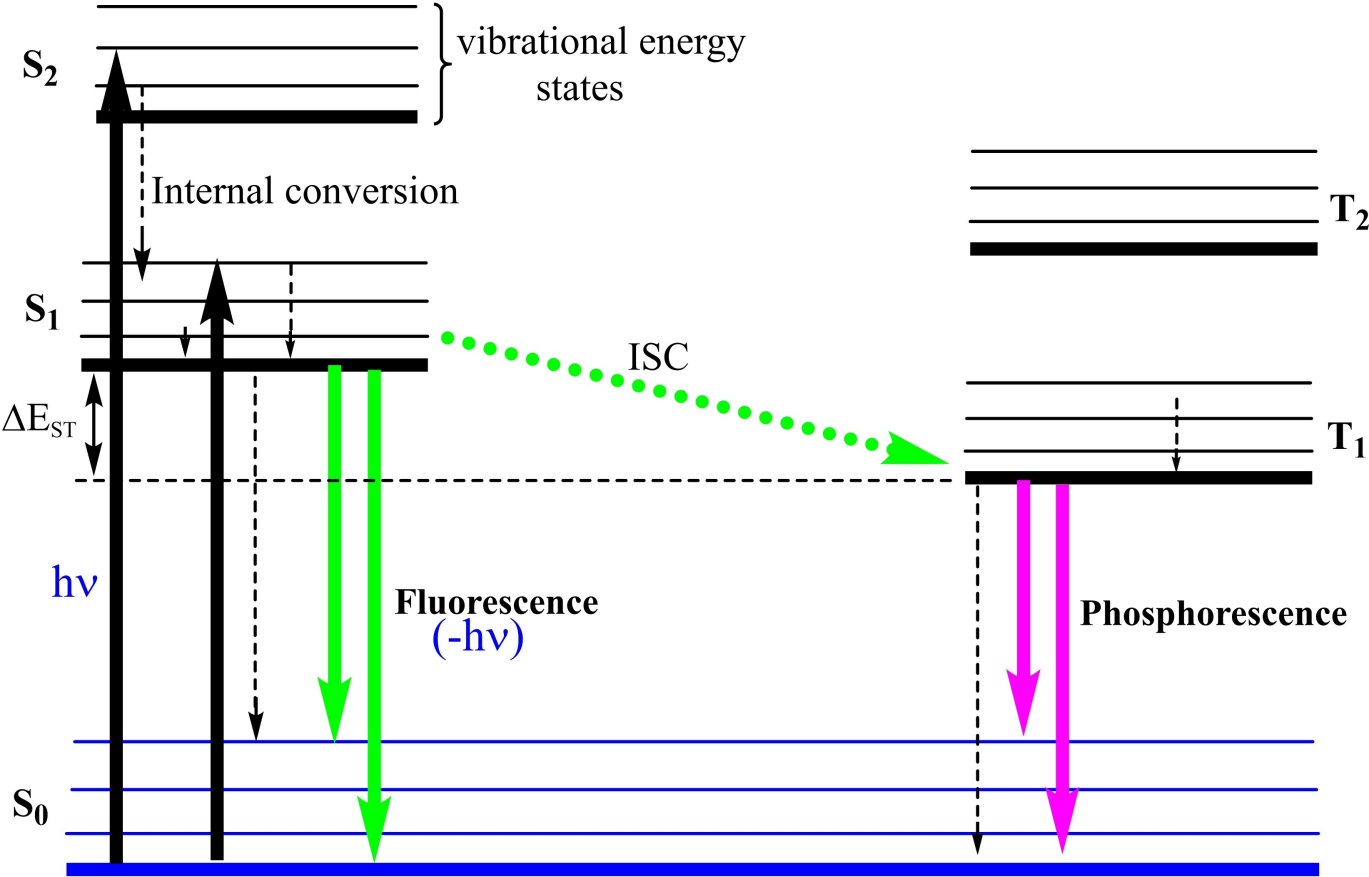
Jablonski diagram showing the phenomena of fluorescence, phosphorescence, internal conversion, and intersystem‐crossing.

Highly luminescent cAAC complexes have been reported in past few years, which have great potential to be used in light‐ emitting devices. Therefore, we will discuss the luminescence properties of cAAC complexes in detail; however, before discussing the luminescence properties of cAAC compounds, let us first discuss the fundamentals of luminescence. The absorption of electromagnetic radiation by a material is governed by Beer‐Lambert law, which can be represented by the equation, $A=log10\left(I0/I\right)=\in Cl$ , where A is the absorbance of light through the material of path length l and concentration C, and ∈ is the molar absorptivity or extinction coefficient of absorbing material. On the absorption of electromagnetic radiation, the electrons undergo transitions to the higher electronic states and return back to the original electronic states by emission of energy in the form of light and heat. The relaxation of electrons results in the observance of luminescence. Luminescence is the spontaneous emission of light from a substance caused by irradiation, chemical reactions, electrical energy, subatomic motions or stress on a crystal. Luminescent materials are commonly called phosphors, which show two radiative processes called fluorescence and phosphorescence (Figure 12). Fluorescence is the type of luminescence observed in molecules, when an electron undergoes transition from an excited singlet state to the singlet ground state. During this transition, the spin state of the molecule does not change, hence it is a spin‐allowed transition, and occurs very rapidly with the lifetime in the range of 10^−9^ to10^−7^ s. Since there is no time lag during the transition, so fluorescence fades away as soon as the illumination is turned off. Phosphorescence, on the other hand, is observed when an electron undergoes a transition from triplet excited state to the singlet ground state. During this transition, the spin state of the molecule changes twice; once during singlet excited state to triplet excited state transition and the other during triplet excited state to singlet ground state transition, and both of these transitions are spin‐forbidden. However, due to spin‐orbit coupling and vibronic coupling among the energy states, these transitions become allowed. Due to the change in spin orientation during transition, the electrons take a much longer time to come back to the singlet ground state, giving rise to a time gap between absorption and emission. Hence, phosphorescence fades after a long time, once the illumination is stopped. Therefore, the lifetime of phosphorescence is much longer than that of fluorescence, and is generally in the range of 10^−3^–10^0^ s. These radiative processes are governed by Kasha's rule, which states that electrons can get excited to any of the excited electronic states (like S_1_, S_2_, S_3_, etc.) depending on the wavelengths of absorbed radiation; however, most of the photons will emit from the lowest excited electronic state (i. e. S_1_ or T_1_). This happens due to rapid internal conversions from higher excited states to lower excited vibrational states. In other words, the emission wavelength is independent of absorption wavelength. The fluorescence intensity originating from a particular electronic level depends on the population of that level. According to the Boltzmann distribution law$\;{(\rho }_{1}/\rho_{2}=e^{-\Delta E/kT})$ when the temperature is increased, the thermal population of higher energy levels increases. The degree of the population of the higher levels also depends on the energy difference between two involving energy levels. The lower energy gap facilitates a higher population in exciting level. Hence, fluorescence intensity originating from those levels is higher compared to lower levels.^[211]^ The efficient phosphors are the ones that have a high fluorescence lifetime and high fluorescence quantum yields. The fluorescence lifetime (τ ) of such materials can be calculated by the equation, $\tau =\left(kr+knr\right)$ ^−1^, where k_r_ and k_nr_ are decay constants for radiative (fluorescence) and non‐radiative emissions. Fluorescence quantum yield is one of the parameters that describe the quality of fluorophores and its possible application in different ways. The superior fluorophores have a higher quantum yield. The fluorescence quantum yield is defined as the ratio of the number of photons emitted to the photons absorbed. The fluorescence lifetime (τ) and quantum yield (φ ) are related to each other that can be expressed by the equation (4). Apart from these two radiative processes, two non‐radiative processes also occur during these transitions. They are internal conversion (IC) and inter‐system crossing (ISC). Internal conversion arises when the electrons descend from the higher energy levels to the lowest energy level within the same excited state. Inter‐system crossing, on the other hand, occurs when the electron undergoes a transition from one state to another, wherein both the states are of different spin states.

(4) $$\varphi =\frac{Kr}{Kr+Knr}=\frac{\tau }{\tau 0}$$

The quantum yield of an excellent fluorophore is very high, but it cannot be ≥1. The quantum yield equal to unity means 100% quantum yield which implies that all the photons absorbed are emitted back. In other words, the total number of molecules excited is equal to the number of photons absorbed. However, this is practically impossible since some of the photons are used up in thermal motions. This is why excellent fluorophores have near 100% quantum yield but not exactly 100%. Frank‐Condon Principle governs the electronic and vibrational transitions which produce absorption and emission spectra. According to the Frank‐Condon principle “the absorption of light is an instantaneous process that allows only rearrangement of electrons but not heavy nuclei, therefore, internuclear distance does not change. Thus, electronic transitions between two levels have more probability to occur when vibrational wave functions of both electronic levels significantly overlap with each other.” For example, if υ =0 vibrational level of one electronic level and υ =2 vibrational level of another electronic level fall in a same vertical line, then their vibrational wave functions will have maximum overlap, hence υ =0 to υ =2 will be the most favored electronic transition. Such transitions are called vertical transitions. It is also important to note that the transitions which are most likely during absorbance are also most likely transitions during fluorescence. For instance, if υ =0 (S_0_) to υ =2 (S_1_) transition is the most likely during absorbance, then υ =2 (S_1_) to υ =0 (S_0_) will be most likely transition during fluorescence. Therefore, fluorescence and absorbance spectra are usually mirror images of each other. This condition is known as the mirror image rule.

**Figure 13:**
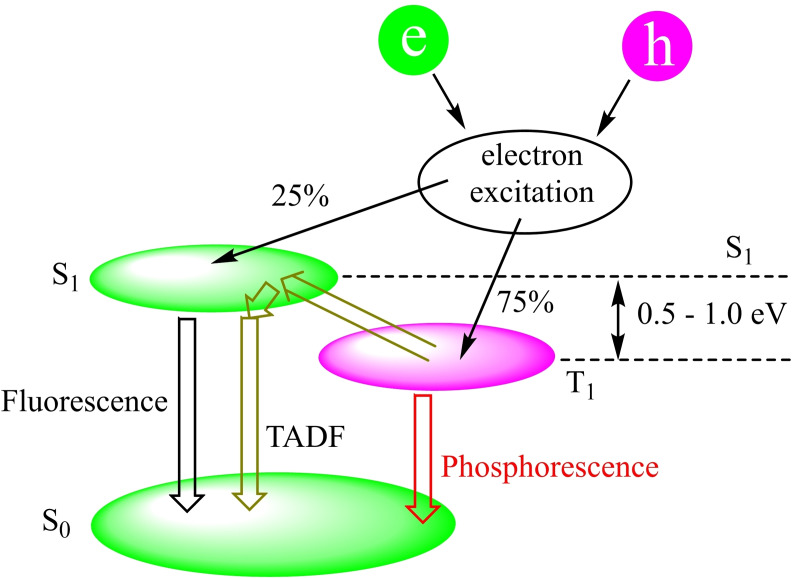
Thermally activated delayed fluorescence. Figure adopted with permission from Ref. [212]. Copyright (2012) Nature Publishing group.

Coinage metal‐based complexes of NHC and cAACs show emission via an alternate pathway called thermally activated delayed fluorescence (TADF); therefore, it is imperative first to discuss this phenomenon. Basically, TADF is the phenomenon of triplet excitons harvesting facilitated by reverse inter‐system crossing (RISC). As we know that the triplet excitons constitute 75% of total excitons (25% being singlet excitons). So, harvesting triplet excitons can dramatically increase the quantum yield. Therefore, chemists try to introduce such a ligand system in the complexes that can show TADF (Figure 13). In the TADF, thermal activation prompts the triplet excitons to convert into singlet excitons via RISC and then decay to the ground singlet state exhibiting phenomena of fluorescence. Since the process of RISC is a slow process, therefore, conversion of once excited triplet (T1) to excited singlet (S1) then decay to the singlet ground state takes time. This is why this type of emission is called delayed fluorescence. This pathway enables all the excitons (singlet and triplet) to undergo radiative decay, giving almost 100% of quantum yield.

Phosphorescent materials, in particular, luminescent copper(I) complexes have found amazing applications in the design of various devices such as sensors,^[213]^ organic light‐emitting diodes (OLEDs),^[214]^ and down converters. In the recent past, coinage metal complexes have been regarded as highly interesting complexes owing to their excellent emission properties due to the absence of metal‐based d‐d* transitions in d^10^ systems, which can lead to premature non‐radiative relaxation, a major problem for d^6^‐Ir^III^ or d^8^‐Pt^II−^ based emitters.^[215]^ Copper(I) luminophores have got increased importance due to a paradigm shift in the interest of chemists in tuning emission properties for the fabrication of optical devices. However, major work has been done on copper(I) complexes of traditional ligands by incorporating different electron‐donating or electron‐withdrawing groups. More recently, NHCs have emerged as powerful emitting ligands for Cu(I) complexes^[216]^ with desirable color‐tuning properties through chemical modification of the NHCs. In this regard, ligand modification such as benzannulation and aza‐substitution of imidazole‐based NHCs has been used to induce bathochromic shift.^[217]^ These modifications stabilize the chromophoric, NHC based LUMO that eventually leads to higher contribution from vacant 2p_z_ orbital of carbene carbon. However, there is an important difference between NHCs and cAACs complexes, NHCs as poor acceptors, give MLCT, which changes metal from d^10^ to d^9^, opening the way to non‐radiative decay, whereas cAAC analogs favor LMCT which causes no change in d‐electron configuration, hence radiative decay. The studies of photophysical properties of NHC‐based Cu(I) complexes by Krylova et al.^[217]^ and Leitl et al.,^[218]^ subsequently, initiated several studies of photophysical properties of NHCs‐ and cAACs‐based Cu(I) complexes.

**Scheme 53:**
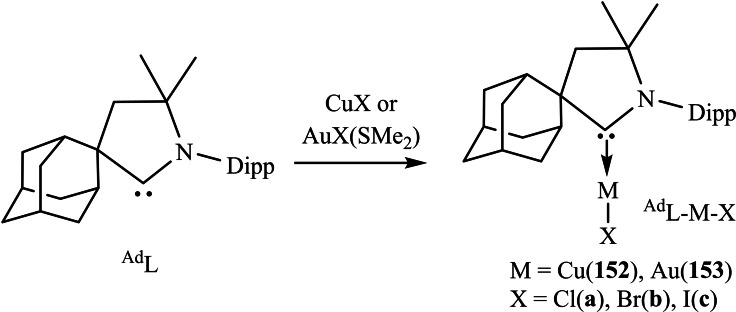
Synthesis of cAAC−Cu−X and cAAC−Au−X complexes.

Recently, a series of two coordinate monomeric copper and gold halide complexes ligated with substituted cAAC have been synthesized, which exhibit impressive phosphorescence properties (Scheme 53).^[219]^ The complexes **152 a**–**c** and **153 a**–**c** were synthesis as shown in Scheme 53. These compounds (^Ad^L−M−X) are isolated in good yields and have good solubility in non‐protic polar solvents (such as DCM, THF), however, moderately soluble in toluene and insoluble in hexane.

The study of photophysical properties of ^Ad^L−M−X reveals that these crystalline compounds show strong photoluminescence on excitation with UV radiation (λ_max_=365 nm). It is interesting to note that complex **152 a** gives a quantum yield of 96%; this is why this compound displays surprisingly small geometric distortion in the excited state which is responsible for the high emission efficiency. The complexes ^Ad^L−Cu−X (**152 a**–**c**) are apparently the first reported examples of cAAC based linear copper(I) halogen complexes which are highly luminescent and give a bluish‐white emission which is independent of the type of halogen atom (Cl, Br or I). On the other hand, phosphorescent gold(I) halide complexes (**153 a**–**c**) display ligand‐dependent red‐shift, in the order X=Cl (blue), Br (yellow‐white), I (yellow).

**Figure 14:**
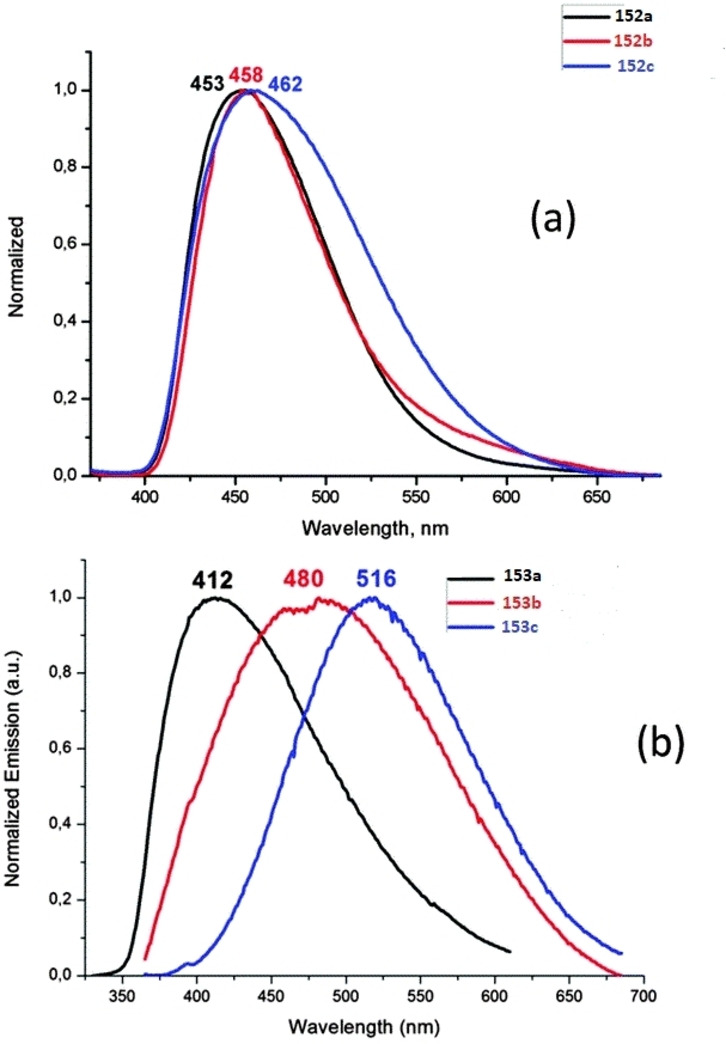
Emission spectra of (a)(^Ad^L)CuX (X=Cl(**152 a**), Br(**152 b**), I(**152 c**), and (b)(^Ad^L)AuX (X=Cl(**153 a**), Br(**153 b**), I(**153 c**) in the solid state (excited at λ_ex_=365 nm. Reproduced with permission from Ref. [219]. Copyright (2016) The Royal Society of Chemistry.

Temperature‐dependent blue shift is a characteristic feature of photoemission by the TADF mechanism due to triplet‐to‐singlet up‐conversion, Δ E (S1→T1)>0. For this phenomenon to occur, the energy difference between S1 and T1 excited state should be sufficiently small, Δ E=K_B_T, so that the effective thermal population of both the states is allowed. Copper(I) based photo emitters have become synonymous with the TADF mechanism. The TADF mechanism provides an effective light harvesting pathway from emitters in which triplet state decay is forbidden. This mechanism can be achieved by reducing the overlap between electron and hole orbitals that will lead to low oscillator strengths and low radiative decay.^[219]^ As we discussed above, copper complexes are characterized by the TADF decay process but photoluminescence of complexes **152 a**–**152 c** was found to be independent of temperature over the range of 4–300 K. The normalized photoluminescence curves for **152 a** at varying temperatures show that emission is because of prompt fluorescence over three orders of magnitude. The luminescence studies of gold complexes **152 a**–**c** reveal that emission from gold complexes, similar to copper complexes, is large via temperature‐independent prompt emission. However, these complexes exhibit very weak photoluminescence with a lifetime of 0.5 ns. The fluorescence rate of gold complexes is found to be much less compared to their copper counterparts. It is evident from the above discussion that cAACs complexes of copper having linear monomeric geometry display surprisingly good phosphorescence properties with a photoluminescence quantum yield up to 96% (Figure 14).

**Scheme 54:**
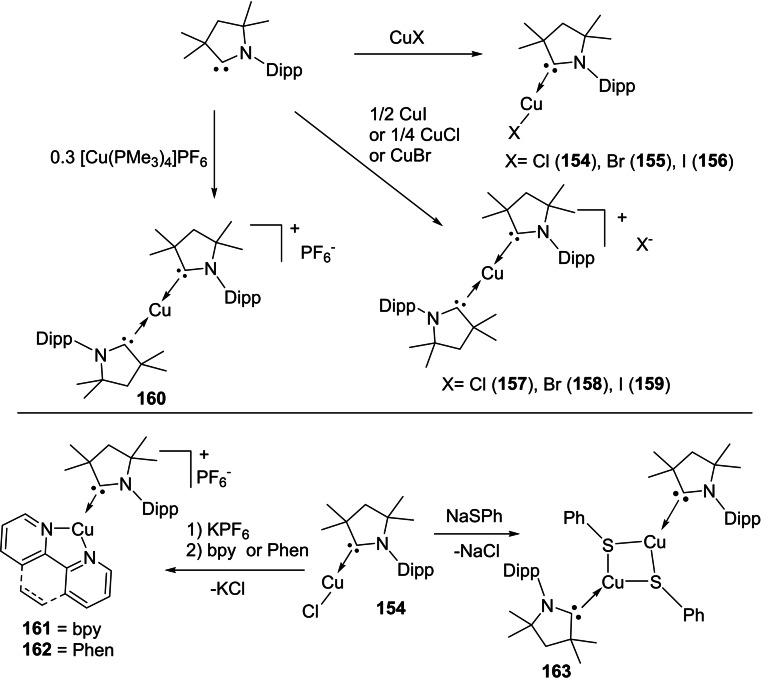
Synthesis of linear **154**–**160**, trigonal **161** and **162**, and dimeric **163**.

Steffen et al.^[220]^ have isolated and investigated a series of copper (I)‐carbene complexes (**154**–**163**) for their photoluminescence properties (Scheme 54). Compounds **154**–**163** were prepared as summarized in Scheme 54. Their investigations have shown that linear and trigonal Cu(I) cAAC^Me^ (cAAC^Me^=1‐(2,6‐diisopropylphenyl)‐3,3,5,5‐tetramethyl‐2‐pyrrolidine‐ylidene) complexes (**154**–**156** and **160**) are vastly different than their NHC congeners in terms of photophysical properties. This is due to the exceptional properties of cAAC^Me^ ligands including highly potent π‐chromophoric properties.^[220]^ Therefore, several Cu(I) cAAC complexes have been tested for optoelectronic applications, and results show that such complexes can be a reliable blue‐emitter in solution‐processed OLEDs. These results indicate that Cu(I) cAAC complexes can be potential precursors for making several optoelectronic components.

**Figure 15:**
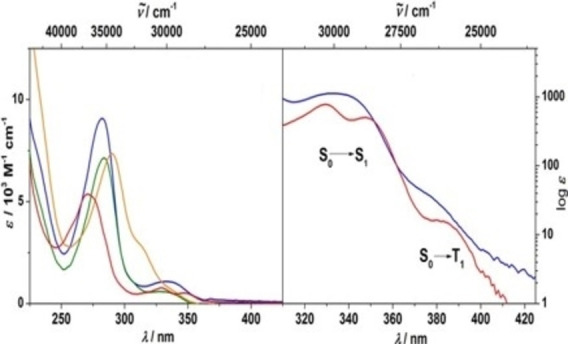
left: Absorption spectra of **154** (blue), **155** (green), **156** (orange) and **160** (red) in THF. Right: Absorption spectra of **154** (blue) and **160** (red) in THF showing the weak S_0_→T_1_ transition. Reproduced with permission from Ref. [220]. Copyright (2017) Viley‐VCH Verlag GmbH & Co. KGaA Weinheim.

**Figure 16:**
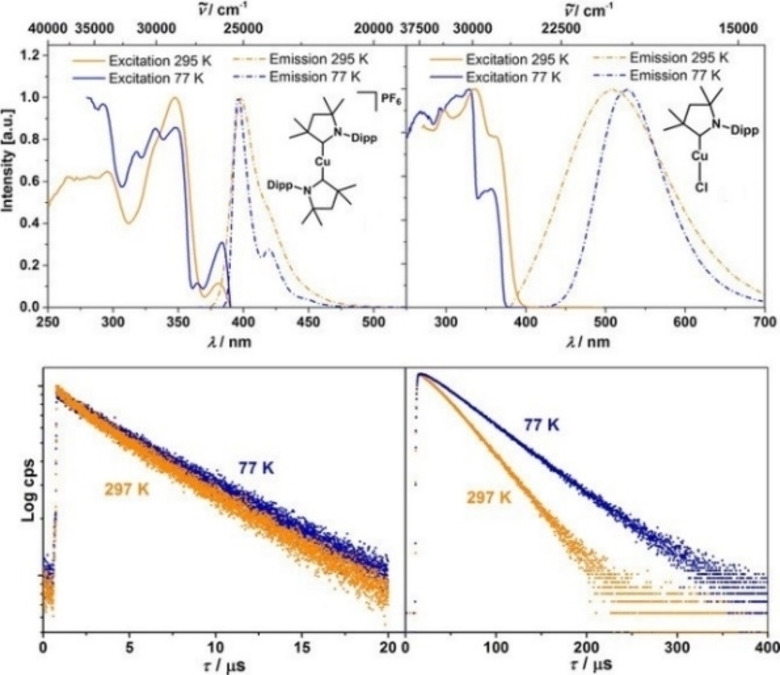
Top: emission (dashed) and excitation (solid) spectra of **160** (left) and **154** (right) in the solid‐state at room temperature (orange) and at 77 K (blue) under argon. Bottom: emission decays at 297 K (orange) and at 77 K (blue) of **160** (left) and **154** (right). Reproduced with permission from Ref. [220]. Copyright (2017) Viley‐VCH Verlag GmbH & Co. KGaA Weinheim.

The linear halide complexes [CuCl(cAAC^Me^)] (**154)**, [CuBr(cAAC^Me^)] (**155**), [CuI(cAAC^Me^)] (**156**) and [Cu(cAAC^Me^)_2_]PF_6_ (**160**) exhibit absorption bands in THF solution at λ_max_=271–290 nm, which are red shifted (lower energy) compared to the free cAAC^Me^ (Figure 15). The absorption bands in the range of 300–350 nm are probably due to forbidden S_0_→S_1_ transitions of metal to ligand transition (MLCT) type (e=0.8–1.4×10^3^ M^−1^ cm^−1^). It is observed that S_0_→T_1_ absorption bands appear in the range of 360–410 nm (e=20–80 M^−1^ cm^−1^) due to strong spin‐orbit coupling which is quite unusual for copper(I) complexes (Figure 16, right).

It is observed that luminescence of complexes **154**–**156** and **160** is quite weak in THF solution compared to other linear copper(I) complexes. However, these complexes exhibit unprecedented phosphorescence with the quantum yield of ϕ_p_=0.33–0.65 at 512 nm (**154‐**‐**156**) and 398 nm (**160**) in solid‐state. The emission lifetime of **154**–**156** is in the range of 6.9–26 ms (Figure 16). Broad emission spectra and S_0_ →T_1_ absorption bands in excitation spectra indicate that emission bands originate from MLCT. Additionally, thermally activated delayed fluorescence (TADF) was not observed by temperature‐dependent lifetime measurements between 297 and 77 K. The TADF phenomena in Cu(I) complexes usually shortens emission lifetime at room temperature due to spin transition from the S_1_ state. This is why lowering of temperature increases emission lifetime by one to three orders of magnitude due to inhibition of reverse intersystem crossing (RISC) T_1_→S_1_. Therefore, the red‐shifted emission spectra at 77 K may be due to vibrational narrowing in solid‐state, and not due to inhibition of TADF at low temperatures. [Cu(cAAC^Me^)_2_]PF_6_ (**160**) is the fastest copper(I)‐based T1‐emitter isolated to date, with an impressive radiative rate constant of K_f_=9.4×10^4^ S^−1^, which is competitive with several Pt^II^‐ and Ir^III^ ‐based emitter. Moreover, the emission of **160** is extraordinary in terms of a sharp rise and surprisingly small half‐width of only 1552 cm^−1^, which has probably not been reported in the literature for copper(I) based emitters. The narrow emission line shapes observed in [Cu(cAAC^Me^)_2_]PF_6_ suggest intra‐ligand states and weak vibrational progression. Lowering in temperature witnesses increased absolute emission intensity, which is due to the temperature dependence of decay rate and changes in non‐radiative decay rate.

**Figure 17:**
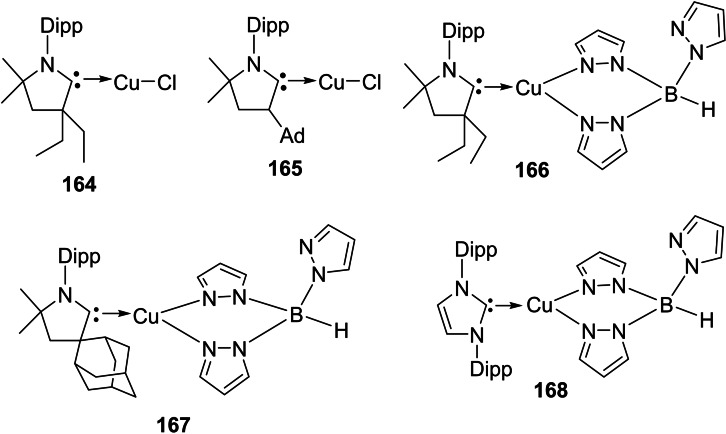
Chlorides and trispyrazolylborates complexes of copper cAACs used in the phosphorescence studies.

Thompson et al. explored cAACs as an alternate class of highly electrophilic carbene ligands for Cu(I) luminophores. Firstly, they investigated previously reported bicoordinate geometry Cu(I) cAAC complexes, **164** and **165** for their photophysical properties in different media and found no evidence of prompt emission. Moreover, they utilized an anionic chelating ligand trispyrazolylborate (Tp) instead of chloride anion to produce higher hapticity complexes **166** and **167** which possess four‐ and three‐ coordinate geometry around the metal center (Figure 17).^[221]^ For the purpose of reference, an analogous NHC‐complex, **168** was also synthesized. Studies revealed that the radiative rate constants for all these complexes are similar (K_r_=2.6–3.9×10^4^ S^−1^) that indicates emission from triplet state. Complexes **166** and **167** show yellow to orange phosphorescence in which **166** is more efficient than **167**. Therefore, cAACs can be efficient promising ligands for attaining yellow and orange phosphorescence.

As we have discussed previously, the photoemissive properties of ^Ad^cAAC ligated copper/gold complexes differ largely from that of NHC analog.^[222]^ The improved emission properties of cAAC complexes are due to better π‐acceptor properties of cAAC ligands. In order to understand the structural relationship of ligand on emission properties, Bochmann et al. have studied copper/gold complexes of cAAC ligand (^Me2^L, ^Et2^L and ^Ad^L) with varying degrees of substitution on cAAC.[^223^, ^224^] Bochmann et al. have isolated and studied properties of a range of halide, pseudo‐halide, aryloxide, and amide complexes for the suitability in fabricating light‐emitting devices.

**Scheme 55:**
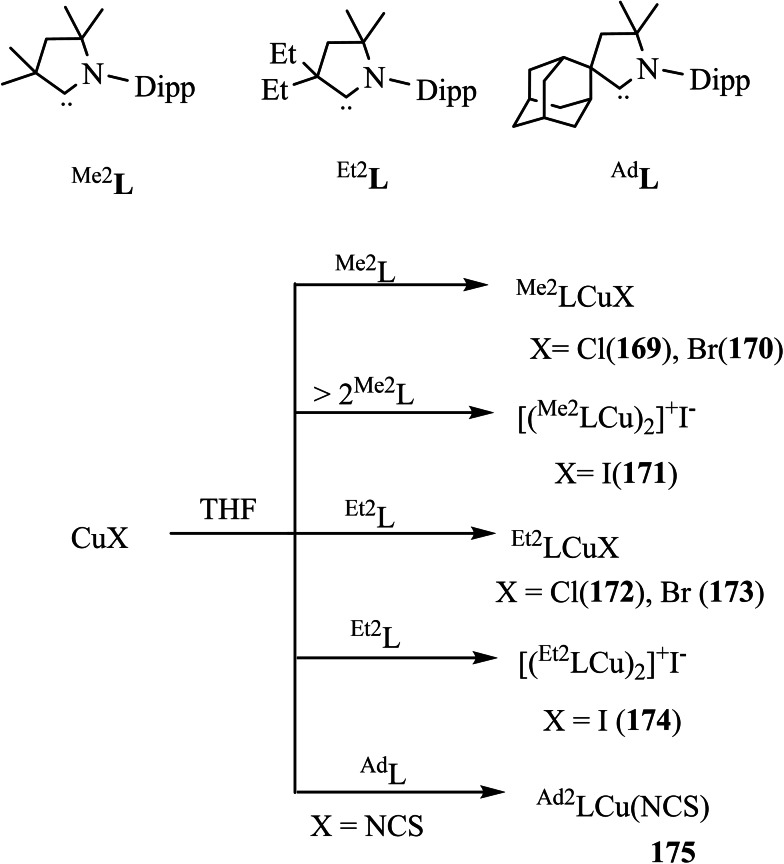
Synthesis of copper halide and pseudo‐halide complexes with substituted cAAC ligands.

Copper(I) halide and pseudo‐halide complexes (**169**–**175**) were synthesized by mixing a solution of respective cAAC ligands with Cu(I) salts in THF, following the procedure previously used for the synthesis of gold halide complexes (Scheme 55).[^225^, ^226^] The halide complexes synthesized using substituted cAAC ligands exhibit good solubility in polar non‐protic solvents such as DCM, MeCN, DMF, THF, or acetone and are moderately soluble in toluene or ethanol but insoluble in hexane.

Here, the luminescence properties of linear monomeric copper complexes have been studied which contain strongly σ‐donating cAAC ligands, designed to raise the d‐orbital energy levels. Since copper halide complexes of ^Me2^L and ^Et2^L are highly sensitive and prone to degradation, therefore photoemission properties of these complexes must be studied with the highly pure sample. While taking pure crystalline samples, the quantum yield increases with the increasing bulkiness of the carbene ligand in order of ^Me2^L<^Et2^L<^Ad^L which can be attributed to the increased rigidity of the molecule. The observations reveal that the gold halide complex, (^Et2^L)AuCl is non‐emissive, in contrast to its bulkier analog (^Ad^L)AuCl and (^Et2^L)CuCl, which are emissive in nature. Moreover, the weakly emissive copper halide complexes (LCuCl) exhibit red‐shifted luminescence. Moreover, as demonstrated by Braker et al. in the recent report, the magnitude of the circularly polarized luminescence in cAAC−Cu(I)−X complexes can be changed by varying the type of X ligand.^[227]^ This suggests that the luminescence properties can be tuned without modification of the main molecular skeleton.

**Scheme 56:**
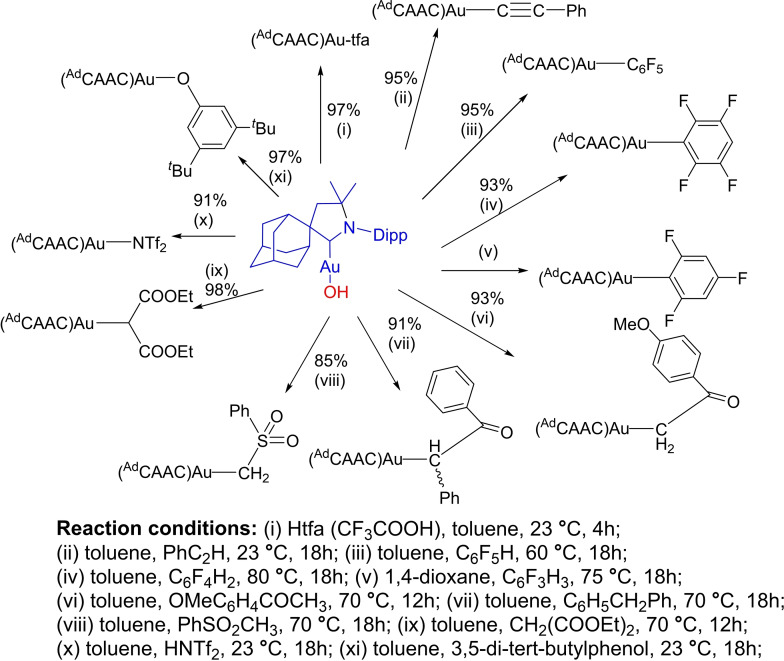
Syntheses of gold(I) A^d^cAAC complexes.

Bochmann et al. have isolated a large number of cAAC stabilized Au(I)^[228]^ and Ag(I)^[229]^ complexes.^[230]^ Au(I) complexes have been synthesized with two less sterically hindered cAAC ligands namely dimethyl (^Me2^cAAC) and the 2‐adamantyl derivative (^Ad^cAAC) (Scheme 56).^[228]^ Bochmann et al. have employed (cAAC)Au(I)Cl complexes as entries into ligand exchange reactions to prepare the corresponding cAAC gold(I) alkoxides, hydroxides, and carboxylates. The substitution of chloride for a more labile oxygen‐containing ligand is synthetically easy and the subsequent reactions benefit from the relative weakness of the Au−O bond.^[231]^

**Scheme 57:**
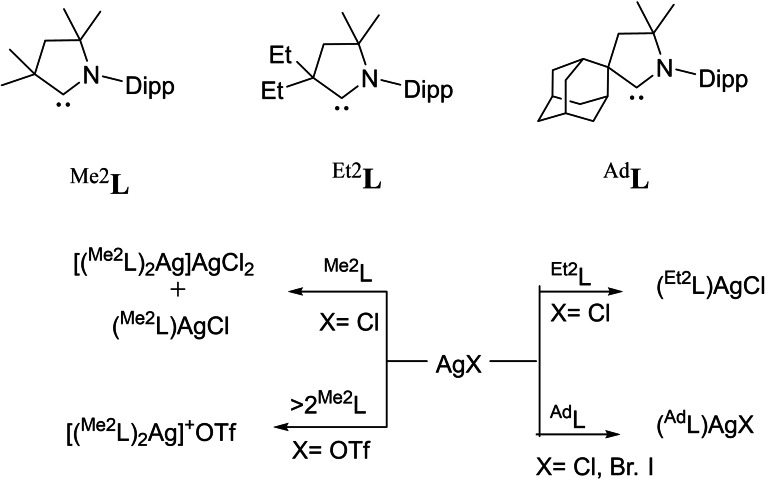
Syntheses of cAAC silver complexes.

Apart from strong photo‐luminescent cAAC‐gold complexes, Bochmann et al. have also synthesized luminescent complexes of silver with cAAC ligands with different degrees of steric hindrance, ^Me2^cAAC, ^Et2^cAAC and ^Ad^cAAC (Scheme 57).^[229]^ The comparative luminescence properties of these complexes have been studied. While ^Me2^cAAC‐silver complexes proved to be non‐emissive, ^Et2^cAAC and ^Ad^cAAC complexes exhibit photoluminescence, which is blue‐shifted by, about 20 nm compared to analogous copper complexes. The strong emissive properties of cAAC stabilized coinage metal complexes motivated chemists to study the organic light‐emitting diodes (OLEDs) performance of such complexes.^[232]^ Consequently, efficient solutions and vacuum‐processed OLEDs have been fabricated using carbene metal‐amide material (CMA).^[233]^

#### 3.11.1 Carbene Metal‐Amides

**Figure 18:**
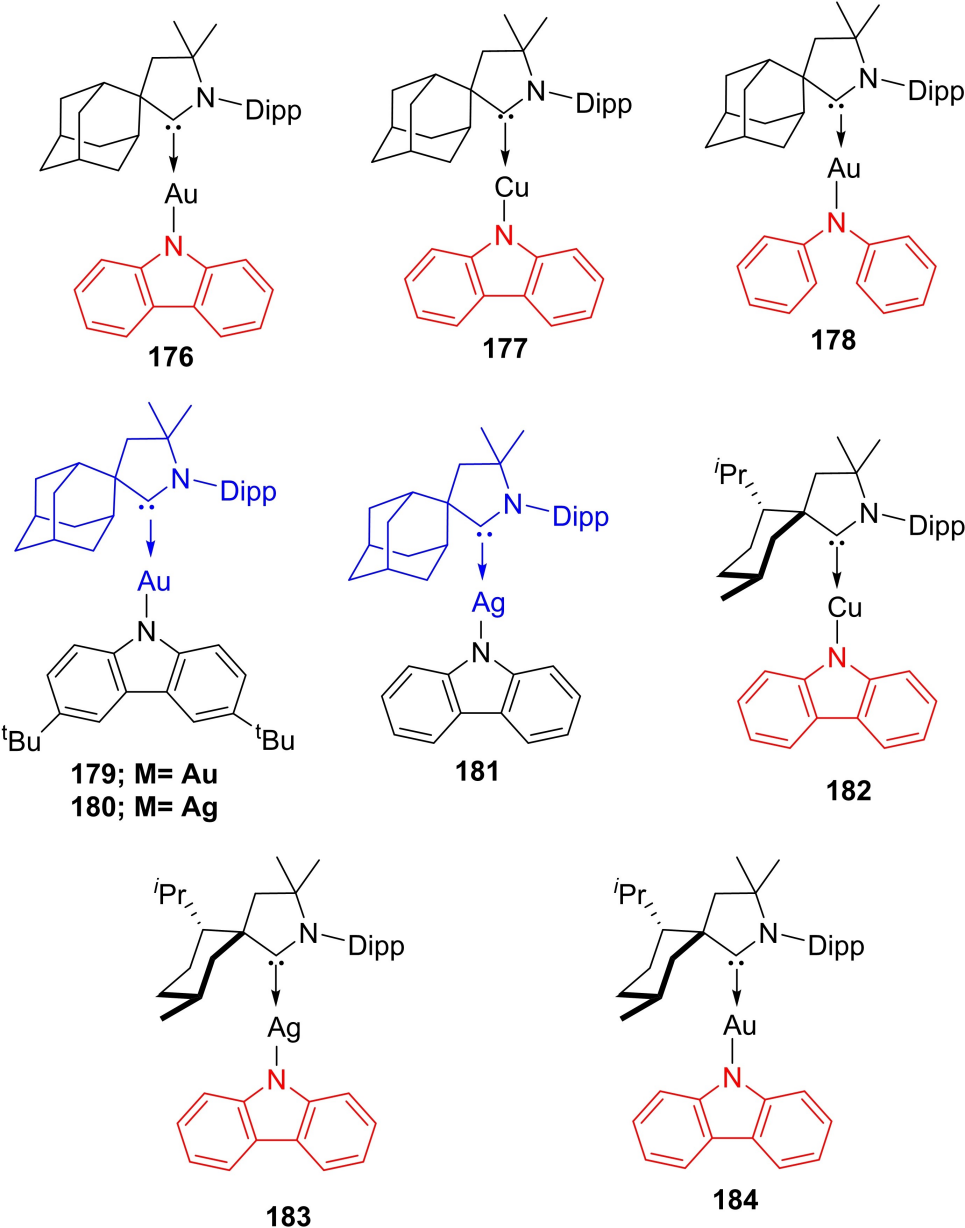
Chemical structures of CMA synthesized by Di et al. (**176**–**179**) and others (**180**–**184**).

Linear coinage metal complexes of the type (cAAC)MX (where X=amide ligand) have recently been recognized as a novel class of emitters that have found utility as highly efficient photon emitters in organic light‐emitting diodes (OLEDs). These linear coinage metal complexes are called carbene metal amide (CMA) and their ability to show delayed emission makes them suitable for OLEDS applications. The process of delayed emission occurs due to charge (electron) transfer from an electron‐rich amide ligand to a LUMO based mainly on the carbene p‐orbital. Numerous kinds of photoluminescent coinage metal complexes are known which can show phosphorescence as well as efficient emission by TADF. OLEDs have been regarded as highly efficient lighting and display technologies. Consequently, efficient vacuum and solution‐processed organic light‐emitting diodes have been fabricated using CMA materials.[^232^, ^233^, ^234^, ^235^, ^236^, ^237^, ^238^] Mainly, cAAC complexes of coinage metals namely copper,[^236^, ^239^, ^240^] gold[^232^, ^239^, ^241^, ^242^, ^243^, ^244^] and silver^[235]^ have been used to fabricate OLEDs. Moreover, dendritic carbene metal carbazole complexes have also been used as photo emitters in the fabrication of fully solution‐processed OLEDs.^[239]^ For example, to explore the efficiency of copper and gold‐based CMA in OLEDs, Di et al. synthesized some CMA compounds such as (cAAC)AuCz (**176**), (cAAC)CuCz (**177**), (cAAC)AuNPh_2_ (**178**), and (cAAC)AuDTBCz (**179**) (Figure 18) (Cz, carbazole anion; Ph, phenyl; DTBCz, 3,6‐di‐tert‐butylcarbazole anion). These compounds are suitable for OLEDs application because they are soluble in a range of organic solvents and do not undergo ligand rearrangement (Table 1). Their thermal stability up to >270 °C and solubility makes them suitable for solution processing. These compounds can undergo vacuum sublimation due to their high sublimation temperature. The applications of CMAs as emitters in OLEDs have been demonstrated by Di et al.^[232]^ by developing a multilayer device structure with all organic layers solution‐processed. The observations show that sub‐micro‐second emission is particularly beneficial for efficient high brightness OLED operation and for avoiding degradation pathways operating due to bimolecular annihilation processes. Some of the important CMA compounds of copper, silver, and gold, which have been studied for their efficiency in OLEDs, are listed in Figure 18.

**Table 1:** Performance summary of OLED devices fabricated by utilizing different CMAs. Reproduced with permission from Ref. [232]. Copyright (2017) American Association for the Advancement of Science.

Emitter	Turn‐on Voltage [V]	EQE [%] (max./100 cd m^−2^)	Current efficiency [Cd/A] (max./100 cd m^−2^/1000 cd m^−2^)	Power efficiency [lumens/watt] (max./100cd m^−2/^1000 cd m^−2^)	Max. luminance [cd m^−2^]
**176**	2.6	26.3/26.1/25.2	76.3/75.8/73.0	62.7/50.0/37.0	44,700
**177**	3.4	9.7/8.9/9.2	30.4/28.0/29.0	11.8/11.7/9.3	7790
**178**	3.0	17.9/17.3/15.5	45.2/43.7/39.1	36.6/25.0/17.0	39,540
**179**	2.6	27.5/26.6/24.5	87.1/84.5/77.9	75.1/50.2/35.5	73,100

**Figure 19:**
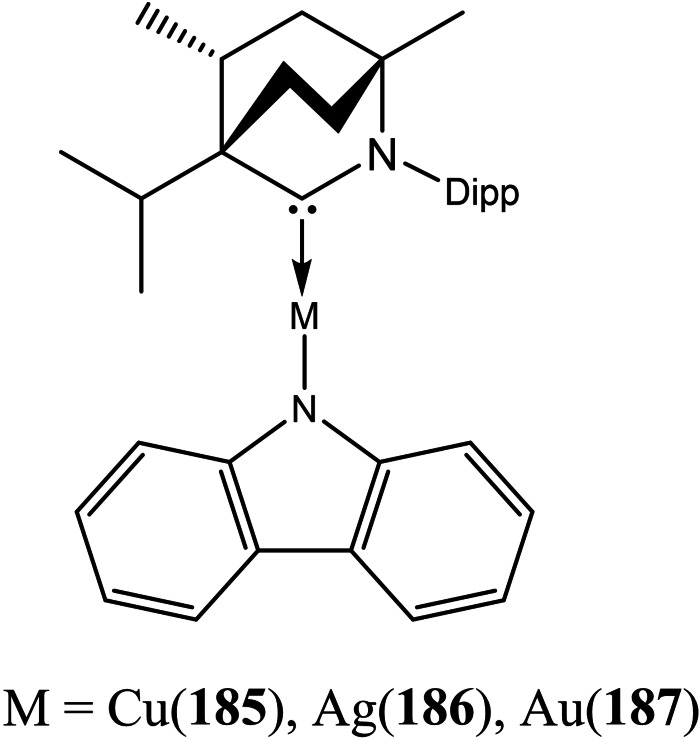
BicAAC stabilized coinage metal‐based CMAs with nearly 100% photoluminescence quantum yield.

The CMAs are emerging as robust emitters with the reports of a new class of cAAC ligands. Recently, comparative studies of metal complexes of coinage metals with cAAC and BicAAC have shown that metal complexes stabilized by BicAACs exhibit better emission properties. Chotard et al. achieved 100% photoluminescence quantum yields in the BicAAC based CMAs (Figure 19).^[245]^ These BicAAC based CMAs (**185**–**187**) are better emitters due to the stabilization of LUMO and the conformational stability of the ligand. These CMAs exhibit a short‐life time in order of Cu>Au>Ag. The selection of metal with high spin ‐orbit coupling and ligand with higher rigidity, is very crucial to achieve higher photoluminescence quantum yields, since, it reduces non‐radiative emission. The chemistry of cAAC based coinage metal complexes has been described by Bertrand and co‐workers^[246]^ and Hossain et al. in the recent reviews.^[247]^ Interested readers may refer to the same.

### 3.12 Radical Chemistry of cAACs

**Figure 20:**
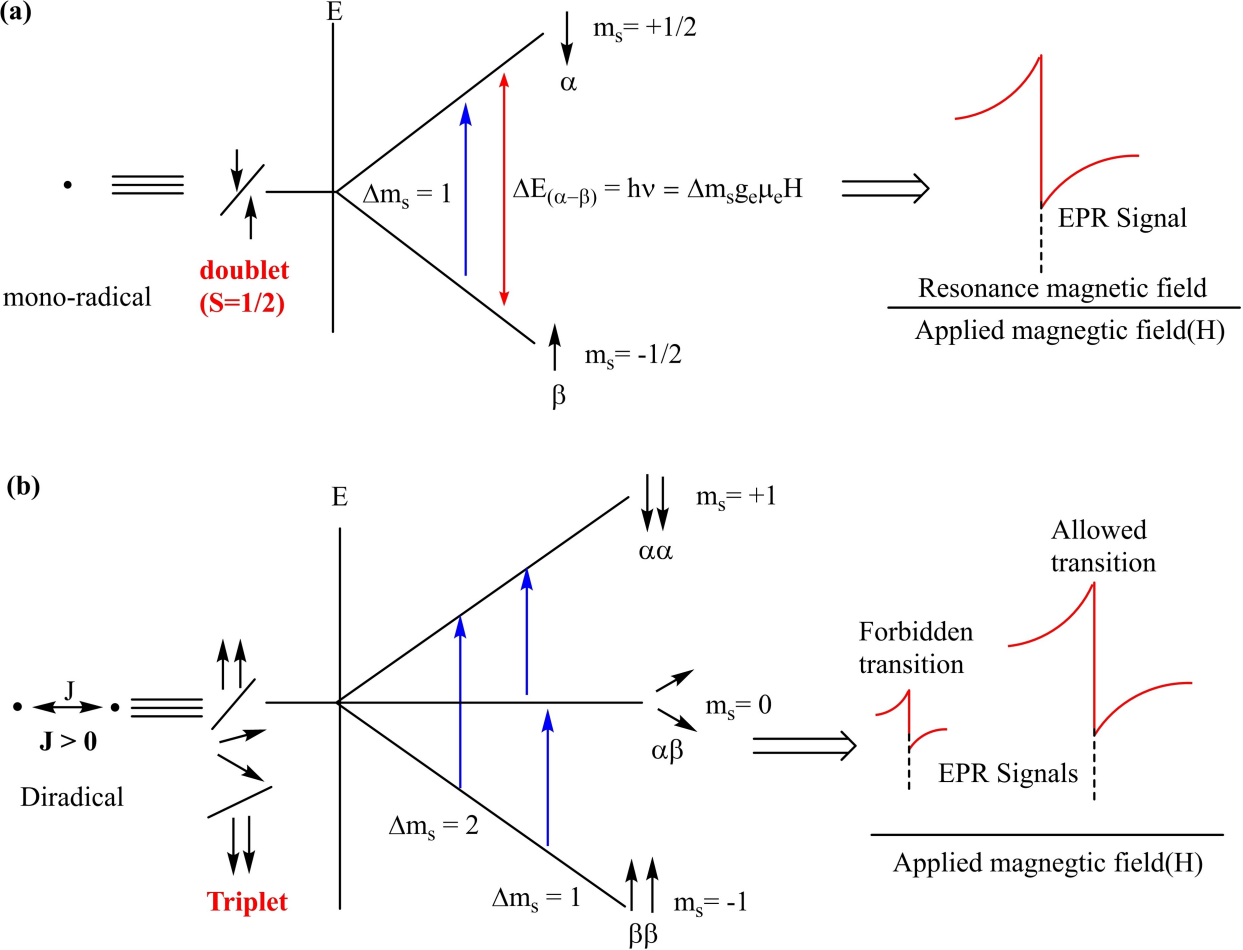
(a) Zeeman effect on single electrons/mono‐radicals and resultant EPR spectrum (b) Inter‐electron coupling in a diradical and resultant EPR spectrum.

Radical species are characterized by the presence of at least one unpaired electron in the molecule. These are the high energy open‐shell species that in most of the cases cannot be isolated, but understanding the nature of these reactive species has tremendous scope in exploring important properties like energy changes, magnetic susceptibility, and reaction mechanisms/pathways. Since radicals are reactive species having lifetimes of around 10^−6^ to 10^−9^ s, studying them poses a major challenge to the researchers. A great deal of knowledge in isolation and analysis techniques had been developed in the past century which makes this part of chemistry continuously emerging and exciting. The first organic radical, trityl radical (Ph_3_P.) was reported by Moses Gomberg in 1900.^[248]^ This radical was discovered serendipitously while synthesizing sterically crowded Ph_3_C−CPh_3_ by reduction of Ph_3_C−Cl with Zn or Ag metal. Electron paramagnetic resonance (EPR) spectroscopy has been instrumental in the characterization of radical species at low temperatures. Unlike nuclear magnetic resonance (NMR), EPR involves the splitting of electronic energy states. The most important advantage of EPR spectroscopy is that it deals with unpaired electrons and is blind to the other paired electrons present in the molecule hence simpler spectra are obtained. The EPR spectra of single‐electron open‐shell species such as mono‐radicals is easy to interpret. However, EPR spectrum of species with multiple unpaired electrons is very much difficult to interpret. Here, EPR spectra of free‐electron/monoradical and diradical will be discussed. A free‐electron is characterized by spin quantum number S=1/2 and possession of electric charge. The free‐electron has magnetic momentum with degenerate magnetic components m_s_=+1/2 and m_s_=−1/2 . In the presence of a magnetic field, the degenerate levels (±1/2) get split up to give well‐resolved energy levels (Figure 20). The splitting of m_s_ levels into well‐resolved energy levels (E_α_ and E_β_) in the presence of an applied magnetic field is known as the Zeeman effect. The energy separation between these levels is directly proportional to the strength of the applied magnetic field(H).^[249]^ The energy difference between E_α_ and E_β_ energy levels is calculated by using equation ΔE=E_α_‐E_β_=Δm_s_g_e_μ_e_H, where g_e_ value is 2.0023 for a free electron. In principle, free‐electron shows one resonance in EPR spectrum. However, in the case of mono‐radical species where an atom, group, or molecule possesses an unpaired electron; the unpaired electron may interact with the magnetic moment of spin active species to give additional hyperfine lines. The hyperfine lines in such cases can be calculated by (2nI+1) [where n=number of spin active nuclear and I=spin of that nucleus]. Generally, radical electron couples with the nucleus on which it is situated, however, it may also couple with neighboring nuclei through bond or space to give further hyper‐fine lines. The number of these hyperfine‐lines can be calculated by (2n_1_I_1_+1)(2n_2_I_2_+1)…. and so on. The hyperfine coupling constant(J) determines the strength of coupling between unpaired electrons and nuclei. The hyperfine coupling between unpaired electrons and nuclei on which it resides is stronger than the coupling with neighboring nuclei. Mono‐radicals are called doublet species due to multiplicity (2S+1) equal to 2.

In the case of biradicals/diradicals, two unpaired electrons reside on a molecule and interact with each other. The independent existence of two unpaired electrons seems like two doublets present within the same molecule. In such cases, the strength of interaction depends on the distance between radical centers. A shorter distance between two radical centers in a molecule enables stronger coupling. However, for biradicals, electron exchange interaction(J) is nearly negligible due to the long distance between radical centers. In the case of higher electron exchange coupling (J>0), two classical situations arise: in one situation both the spins align in the same direction (S=1, 2S+1=3) making it a triplet diradical and in another situation both the spins align opposite to each other (S=0, 2S+1=1) making it a singlet diradical (Figure 20b). This electron spin behavior of radical centers can be seen in the well‐resolved EPR spectrum.

#### 3.12.1 Monoradicals, Diradicals, and Diradicaloids

Mono‐radical is an atom or a functional group or a molecule with an unpaired electron. Radicals are an important class of chemical species which act as intermediates in several chemical and biochemical conversions. Most of the radicals have extremely short lifetimes and are unstable. Therefore, most of the radicals cannot even be characterized by electron paramagnetic resonance (EPR) spectroscopy. The radical species which have a relatively long lifetime can be characterized by EPR spectroscopy at low temperatures. However, such radicals can be stabilized and isolated in the pure form with the help of suitable stabilizing ligands as summarized in a recent review.^[159]^ The most common strategy to stabilize transient species is substitution on radical by bulky groups so that dimerization or hydrogen abstraction is prevented. Most of the radical species undergo dimerization to stabilize themselves. The stabilized radicals of such kind have a longer lifetime.

When two radical centers within a molecule interact strongly then they are called diradicals. In such species, two electrons occupy the two non‐bonding degenerate orbitals at the radical centers. This degeneracy gives rise to interesting phenomena in the molecule. The two electrons have an S value of 1 and hence can give rise to 3 states in the presence of the external magnetic field. The spin selection rule allows only transitions which have the value Δm_s_=1 and −1, however, the forbidden transitions are also visible from the EPR spectra which have lower intensity since they come from Δm_s_=2. The type of species i. e., whether singlet or triplet is determined by the energy difference between the two states. Since the distance between the two radical centers is less, the electron exchange interaction would be dominant and there will be considerable orbital overlap. This will result in the preference for the triplet state and this triplet state is more stable than the singlet state and the energy difference exists between the two states. The knowledge of the singlet ‐ triplet gap in the diradical molecules is essential since it gives the magnitude of overlap of the orbitals and the electron exchange interactions. The analysis of the energy gap of singlet‐triplet in open‐shell species can be achieved by using various techniques like matrix isolated IR spectroscopy at low temperatures, time‐resolved UV spectroscopy, EPR spectroscopy, and Negative ion photoelectron spectroscopy (NIPES). However, the most suitable techniques are NIPES and EPR spectroscopy. Singlet diradicals have a relatively small energy gap between singlet and triplet states. The diradical species with a higher HOMO‐LUMO gap tend to be more stable due to larger singlet‐triplet separation. When LUMO occupancy of such chemical species becomes zero, they are termed as closed‐shell molecules. Such molecules are not considered as diradicals or diradicaloids.

Diradicaloids, on the other hand, have two radical centers in the same molecule but the orbitals are slightly non‐degenerate. Therefore, in diradicaloid the two electrons occupy preferably in the singlet state in contrast to diradicals. Electronic stabilization has a dominant role over the electron‐electron repulsion and this favors the singlet in these species. The energy difference between the two orbitals is determined by special and through‐bond interactions. The diradical nature of these species can be estimated by the natural orbital's occupancy of the LUMO. The magnitude of the singlet‐triplet gap also provides information on the extent of orbital interaction. Diradicaloids are characterized by a small HOMO‐LUMO gap and are more reactive than closed‐shell molecules.[^250^, ^251^] Diradicaloids are organic molecules with two unpaired electrons interacting with each other within a single molecule which are fundamentally important in the understanding of the nature of chemical bonding.[^252^, ^253^]

**Figure 21:**
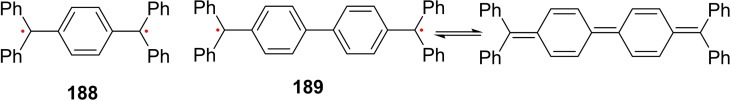
Thiele's and Tschitschibabin diradical.

The discovery of the first radical by Gomberg in 1900, prompted a great interest of chemists in this exotic field. Thiele et al. reported diradical molecule **188** in which two radical centers were separated by a p‐phenylene bridge.^[254]^ Later on, Tschitschibabin reported an organic diradical **189** in which two radical centers were separated by a biphenylene bridge (Figure 21).^[255]^ Since then organic diradicaloids/diradicals have triggered many controversies and the nature of the diradical paradox has been a matter of great discussion.^[256]^ In fact, **189** can be considered either as a diradical or cumulene structure in which loss of aromaticity can be negated by spin‐pairing.^[257]^ However, Tschitschibabin hydrocarbon **189** is nowadays considered as a diradicaloid.^[258]^ Diradicals have been divided into two categories; localized and delocalized diradicals. Delocalized diradicals have been further divided into Kekule' and non‐Kekule' type species. Antiaromatic molecules are considered delocalized diradicals. Recently, Jana et al. reported cAAC‐based Thiele and Schlenk diradicals^[259]^ in which radical centers are present on carbene carbon. These radical centers are separated by a phenylene ring.

**Scheme 58:**
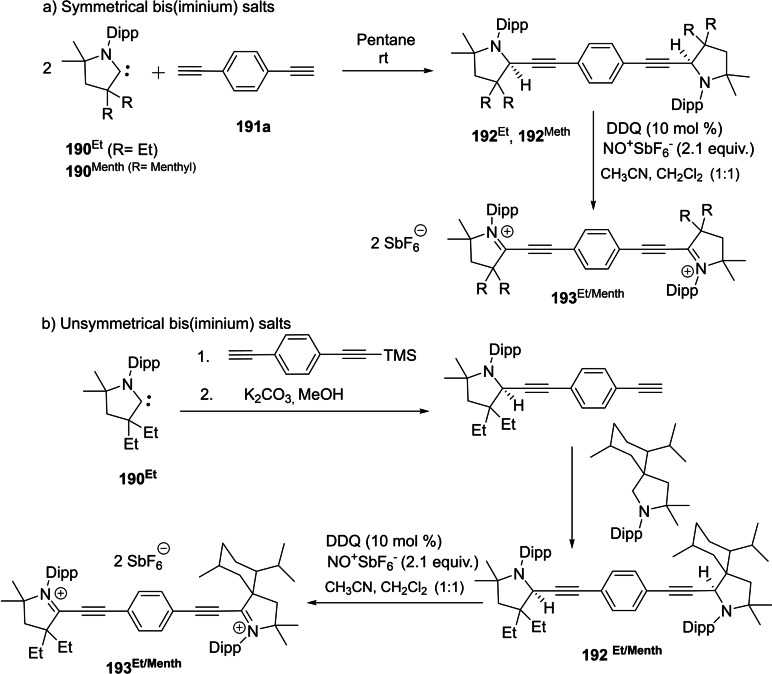
Diradicaloids synthesis.

The strong σ‐donor nature of singlet carbenes becomes helpful in stabilizing highly reactive radical species. cAACs are regarded as a stronger σ‐donor and a better π‐acceptor in comparison to the N‐heterocyclic carbenes (NHCs). Therefore, cAACs successfully stabilize transient species. The cAAC derived diradicaloids are very less reported. Most recently, Bertrand et al. reported Kekule diradicaloids/diradicals derived from cAACs using a modular approach. The key step in this synthesis is the insertion of cAAC into the C−H bond of two‐terminal alkynes separated by a spacer. In a further step, hydride abstraction is involved followed by two‐electron reduction of the corresponding bis(iminium salt) that gives rise to the desired diradical.^[260]^ Indeed, this route allows synthesizing of unsymmetrical compounds featuring two different cAACs linked by a spacer. Interestingly, the properties of diradicaloids approach towards those of mono‐radicals on increasing spacer length.^[260]^ They are stable at room temperature, however, readily react with oxygen. To access a broad range of such diradicaloids, easy tuning of carbene and spacer is really important and it can be achieved by a modular approach. Bertrand et al. have shown that C−H bond activation of alkynes by carbenes especially cAAC **190** is highly selective. Therefore, it was considered that double C−H bond activation of a diyne spacer **191** resulting in **192**, will give **193** after undergoing two hydride abstraction. The final obtained bisiminium salt **193** gives the desired diradicaloids after two‐electron reduction. It is noteworthy that in the synthesis of such diradicaloids, the main step is the activation of terminal alkyne with the cAACs that allow installation of the spacer with different degrees of aromaticity and length in between two similar or dissimilar carbenes. Hence, this synthetic route gives the opportunity of generating unsymmetrical compounds with two different cAACs (Scheme 58).^[260]^

**Figure 22:**
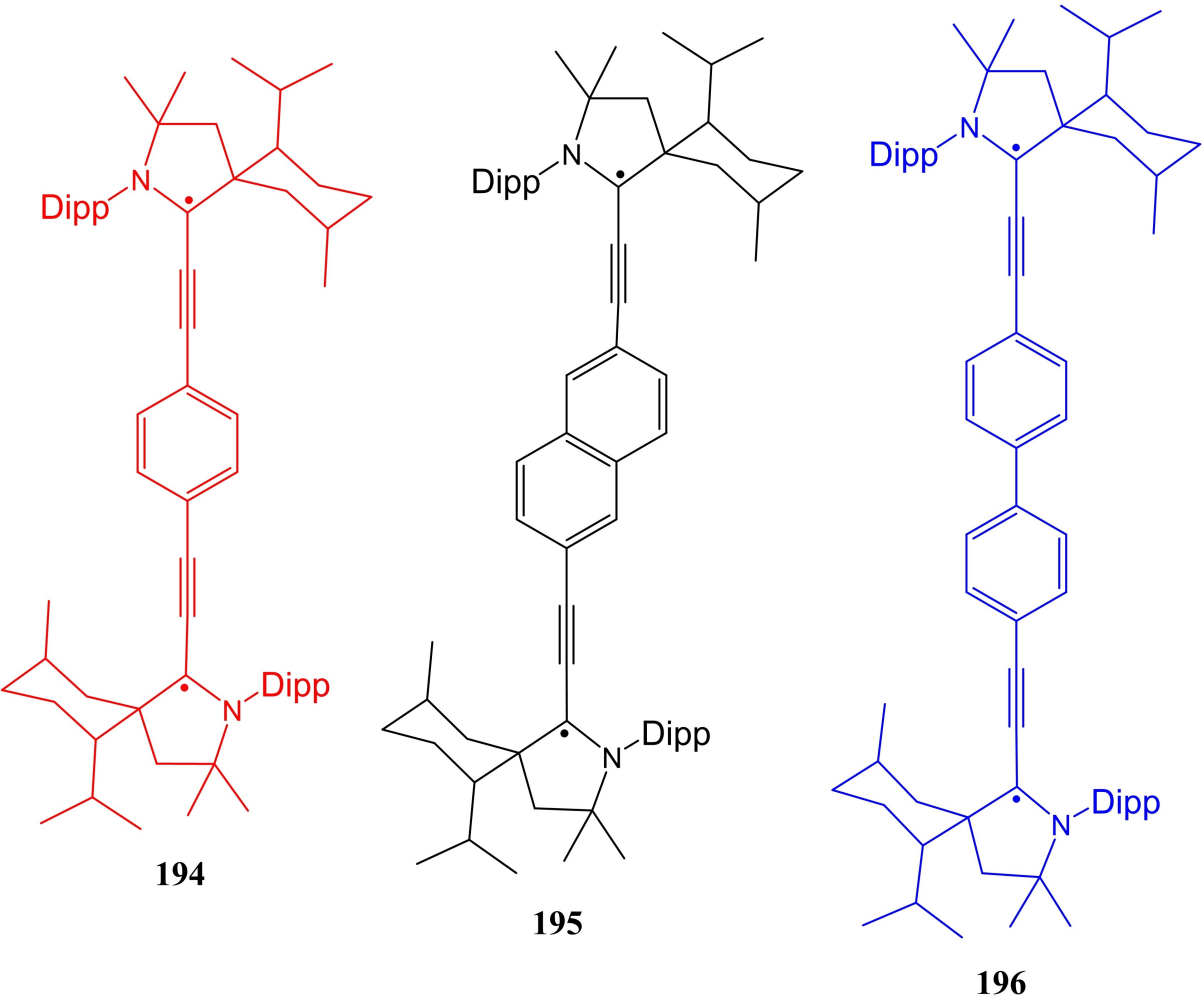
cAACs based diradicaloids synthesized by using different linkers.

Hansmann et al., in a recent work, have reported the synthesis of cAACs based diradicaloids using different spacers which separate radical centers and they have studied the singlet‐fission property of these systems.^[261]^ The three diradicaloids (**194**–**196**) (Figure 22) were synthesized using different spacers namely phenylene, naphthalene, and biphenylene. The photoluminescence spectra of these diradicaloids show significant red‐shift on increasing linker/spacer length. This red‐shift can be attributed to the decrease in the energy gap between

HOMO and LUMO, which leads to a significant increase in diradical character. The linker between radical centers allows through‐bond coupling between two radical centers. However, in the case of the singlet‐fission, the singlet excited state changes to two separate excited triplet states with correlated triplet states ^1^(T_1_T_1_), consequently, radical centers couple through space.

**Scheme 59:**
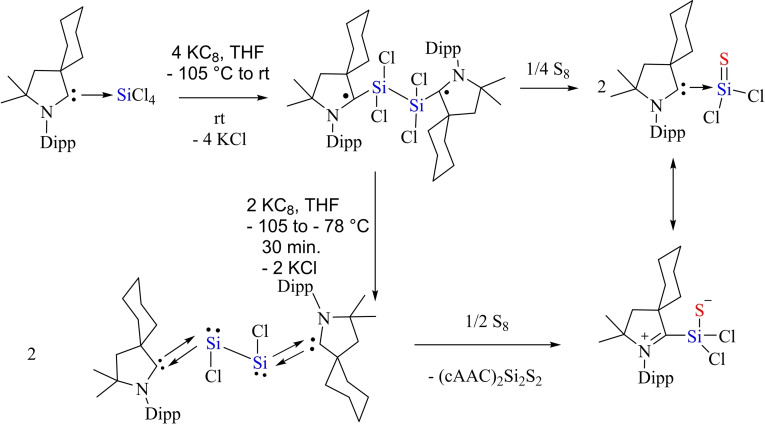
Synthesis of cAAC disilicon radical and its reaction with sulphur.

Similarly, disilicontetrachloride has been used as a spacer to stabilize biradicals; wherein, the cAAC stabilized biradical of disilicontetrachloride possesses the radical centers on the carbene carbon.^[262]^ This biradical converted in (cAAC)SiSCl_2_ in the presence of sulfur (S_8_) at low temperature (Scheme 59).^[263]^ Bertrand's group has also achieved the coupling of NHC and cAAC resulting in the formation of heterodimers which have been used to isolate radical cations.^[264]^ Moreover, phosphorus^[265]^ and boron‐centered radical cations have also been isolated in which the radical center is flanked by two cAAC ligands. For instance, Bertrand et al. isolated a cAAC based crystalline phosphinyl radical cation.^[266]^ In 2013, we reported^[267]^ a singlet biradicaloid siladicarbene, (L:)_2_Si which is stabilized by two carbene molecules, and silicon is in zero formal oxidation state. The crystals of this biradicaloid are stable in the air for about a day. This unique species gives the roadmap for the carbene‐stabilized biradicaloids that exhibit unexpected stability. Additionally, we have also reported stable and isolable trichlorosilylcarbene radicals, (cAAC⋅)−SiCl_3_ and cumulene‐based radical cations and dications that contain cAACs.[^268^, ^269^] Bertrand et al. have successfully utilized cAACs to generate (amino)(carboxy)radicals,^[270]^ which are air‐persistent and do not dimerize and could be characterized for the first time by X‐ray diffractometry as monomeric species.^[271]^ These (amino)(carboxy)radicals can be stored for weeks at room temperature, demonstrating that the (amino)(carboxy)radicals can be considered as stable monomeric paramagnetic building blocks, similar to verdazyl and nitroxyl radicals.

**Scheme 60:**
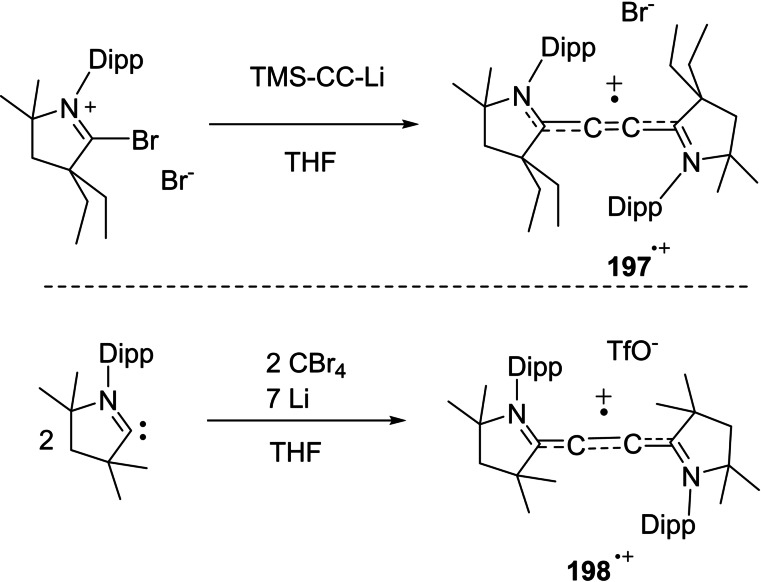
cAAC radicals isolated by Bertrand et al. (**197**) and Roesky et al. (**198**).

Creutz and Taube[^272^, ^273^] have done pioneering studies in the field of bimetallic mixed‐valence systems that has got much attention from chemists. Although, organic compounds with mixed‐valence nature have been known for decades, however, their applications have been really difficult due to their sensitivity to oxygen and lack of thermal stability.^[274]^ To demonstrate the metal‐like property of cAAC_S_, Bertrand et al.^[275]^ (**197**) and Roesky et al.^[269]^ (**198**) have prepared organic mixed‐valence systems. Bertrand et al. reacted cyclic C‐bromo‐iminium bromide with a freshly prepared THF solution of lithium trimethylsilyl acetylide (TMS−CC−Li) at −78 °C in THF (Scheme 60).^[275]^ The reaction shows color change from pale yellow to deep red within a few minutes at room temperature. The volatile components were removed and the mixture was extracted with DCM to get a red solid compound (**197**) with 40% yield (Scheme 60).

**Scheme 61:**
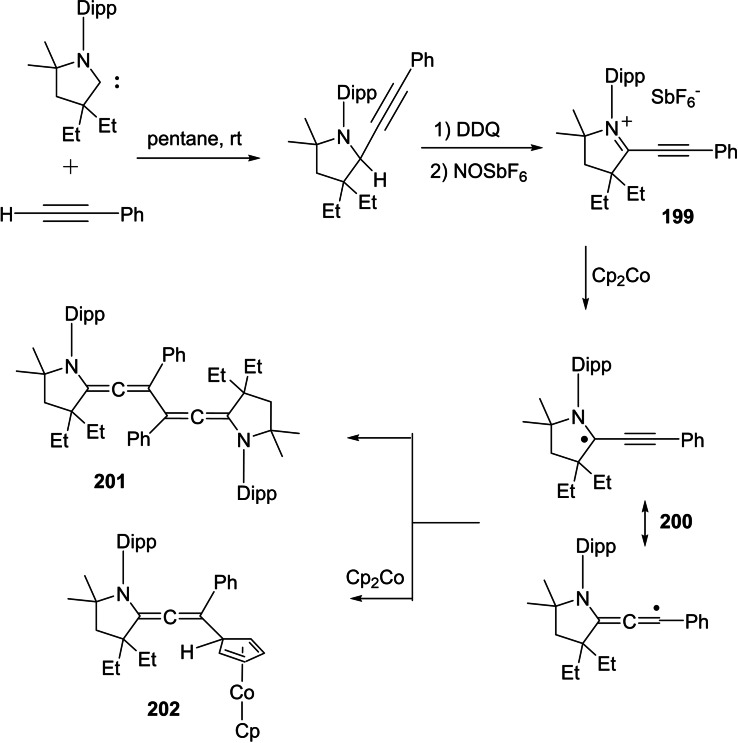
Synthesis of alkynyl‐iminium salt (**151**) and subsequent synthesis of short‐lived amino‐allenyl radical (**152**), its dimer (**153**), and trapping product (**154**).

Recently, crystalline monomeric allenyl radicals have been synthesized. Bertrand et al.^[18]^ showed that using the right set of substituents, the pyrrolidine scaffold gives allenyl radicals that are stable at room temperature and can be isolated even in the solid‐state. In order to synthesize the desired allenyl radicals, they chose the reduction method which has been already used for carboxy radicals. The target alkynyl‐iminium precursor **199** was prepared in two steps as shown in Scheme 61. The air‐sensitive, red color solution of allenyl radical was obtained on chemical reduction of **199** with one equivalent of cobaltocene, or half an equivalent of tetrakis(dimethylamino)ethylene. The EPR spectrum measurement was used to confirm the formation of a paramagnetic species (allenyl radical) (**200**). It was observed that this radical species is short‐lived at room temperature (Scheme 61).^[18]^ This species dimerizes in a few hours via the allenyl carbon giving **201**. Interestingly, radical **200** can be trapped by another equivalent of cobaltocene to give **202**.

#### 3.12.2 Persistent Silylene Radical Anions

**Scheme 62:**
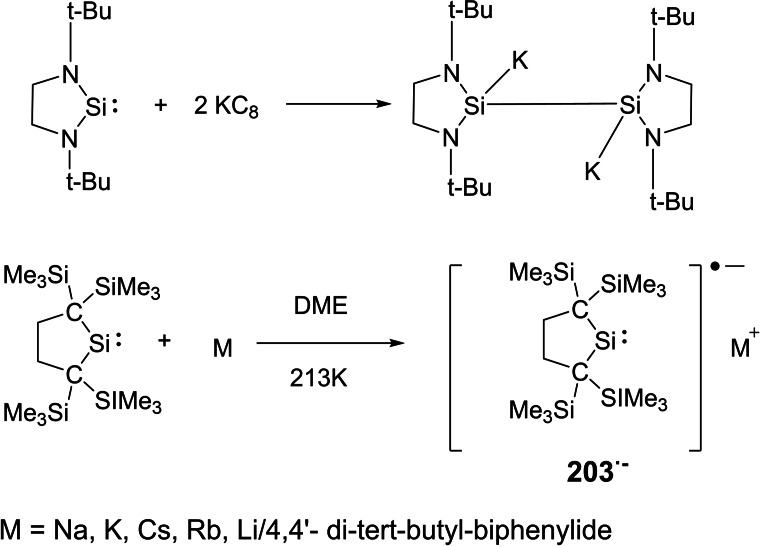
Synthesis of persistent radical anion of dialkylsilylene.

The existence of radical anions and analogs as reactive intermediates in redox reactions has been speculated since early 1970. The carbon‐based radical anions have been reported for several decades. However, recent past has witnessed a huge interest of chemists in heavier group 14 analogs of carbenes and radical anions. The synthesis of such radical ions is highly challenging due to their high reactivity and air sensitivity. The silylene and analogous singlet group 14 divalent compounds contain low‐lying vacant p_π_ orbitals. These compounds are expected to form single‐electron reduction radicals. Kira et al. synthesized a radical anion of isolable cyclic dialkylsilylene **203** ^.−^ generated by the one‐electron reduction of the corresponding silylene (Scheme 62).^[276]^ Alkali metals were used as electron sources in the reaction. This anion radical is relatively persistent at lower temperatures. The unique structural properties of in situ generated radical anion **203** ^.−^ have been studied by EPR spectroscopy at 213 K with an intense EPR spectrum corresponding to g=2.0077. Radical anion **203** ^.−^ is stable at 70 °C dimethoxyethane; however, it readily decomposes at room temperature with a 20 min half lifetime.^[276]^

**Scheme 63:**
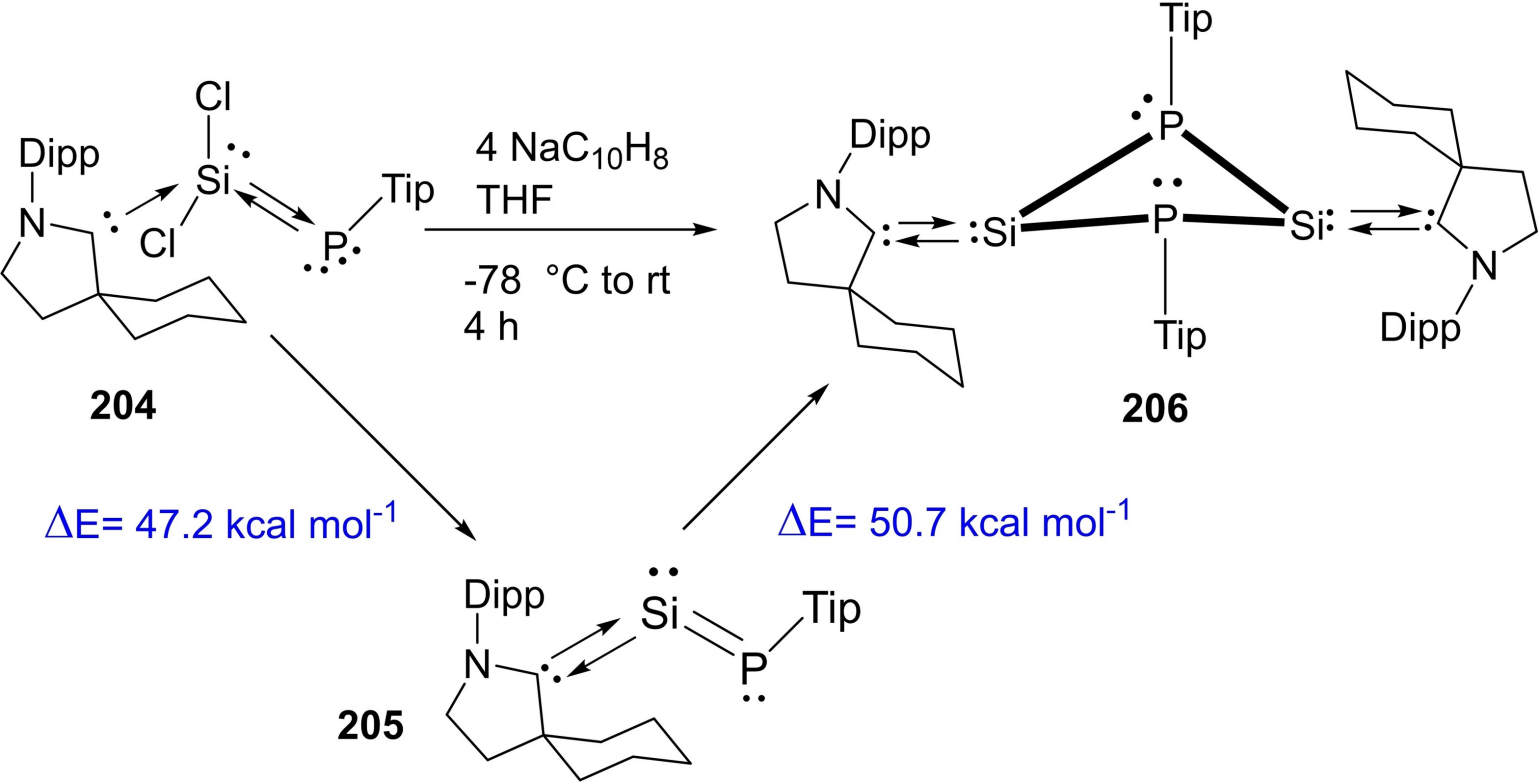
Synthesis of dimer [(cAAC)_2_Si_2_(P‐Tip)]_2_ (**206**).

Until recently, carbene stabilized phosphorus‐silicon species were rarely studied. However, in the last decade, chemist synthesized another stable carbene by replacing σ‐withdrawing and π‐donating nitrogen atom of the NHC. This gave rise to stable cAAC with highly desirable electronic properties. The NHCs and cAACs are inherently different; the HOMO‐LUMO gap in the latter is comparatively small that gives them an edge in terms of donation ability. Keeping in mind these fundamental differences, chemists have usually used cAACs to stabilize silicon and phosphorus species. Recently, we have synthesized a cAAC stabilized phosphorus‐silicon radical anion (**206** ^.−^).^[277]^ In order to synthesize this radical anion, cAAC‐dichlorosilylene stabilized phosphinidene (cAAC)SiCl_2_→P‐Tip) [2,4,6‐triisopropylphenyl] (**204**) was subjected to the two‐electron reduction to form (cAAC)Si=P‐Tip (**205**). This monomeric species **205** can be regarded as a heavier ketenimine (R_2_C=C=N−R) analog with Si and P atoms in place of C and N atoms, respectively. It is surprising to note that dark red color crystals of a dimer [(cAAC)Si(P‐Tip)]_2_ (**206**) were isolated instead of monomer **205** (Scheme 63).

**Figure 23:**
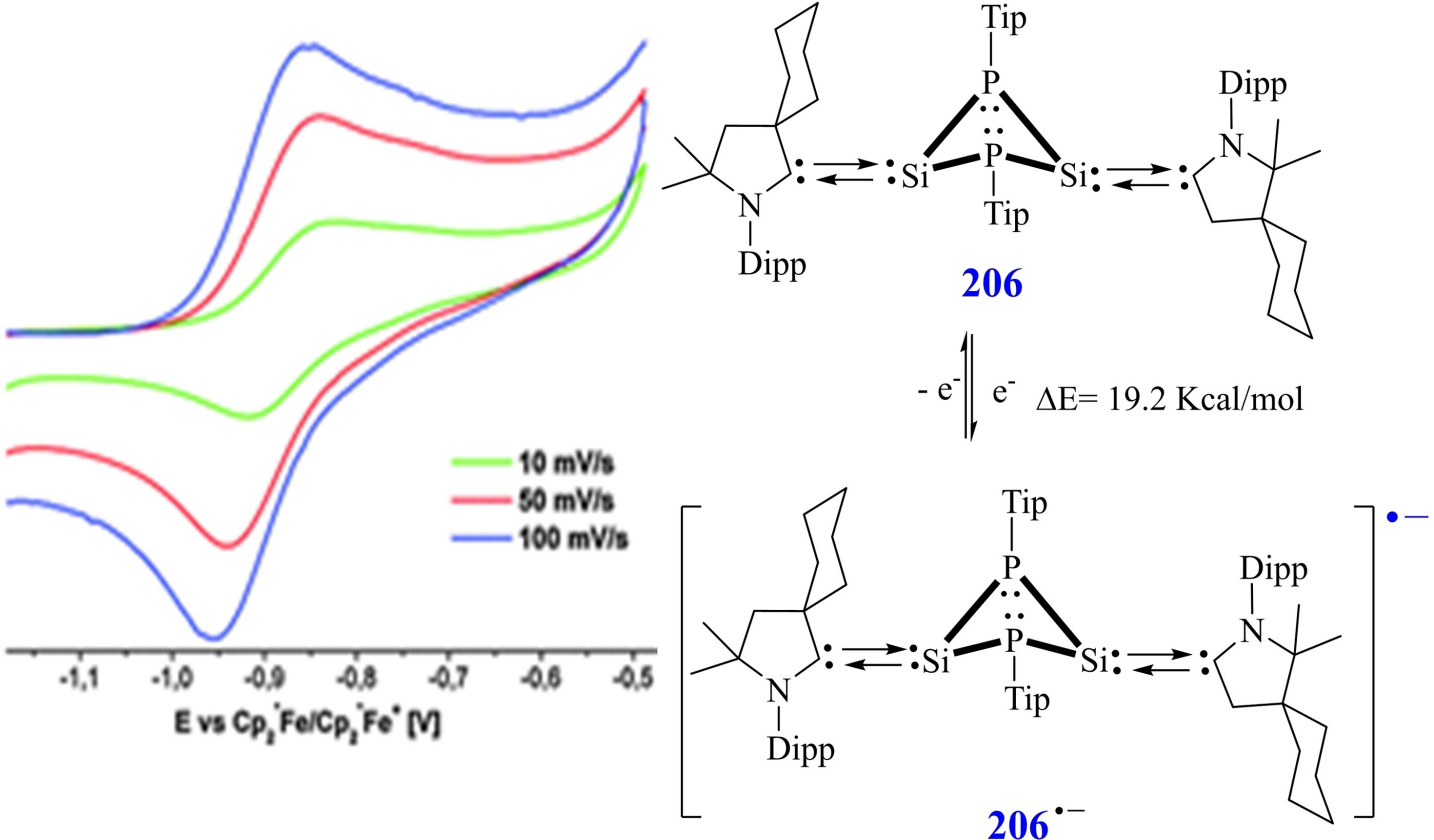
Section of cyclic voltammogram of THF solution of **206** at indicated scan rates, containing 0.1 M [n‐Bu_4_N]ClO_4_ as electrolyte. Reproduced with permission from the Ref. [23]. Copyright (2015) American Chemical Society.

The dark red colored crystals of **206** are air‐stable for about 30 min and afterward turn into a colorless solid. However, it is stable for months in n‐hexane solution at −32 to 0 °C and under inert atmosphere, crystals can survive for 2 months at room temperature. The temperature‐dependent magnetic susceptibility measurement of **206** reveals its spin ground state S=0. The **206** was also studied by cyclic voltammetry (CV) in THF solution containing [n‐Bu_4_N]ClO_4_ electrolyte in 0.1 M concentration. The cyclic voltammogram of **206** ^.−^ exhibits one electron quasi‐reversible process at E_1/2_=−0.87 V against Cp*_2_Fe/Cp*_2_Fe^+^, indicating formation of radical anion **206** ^.−^ (Figure 23).^[277]^

**Figure 24:**
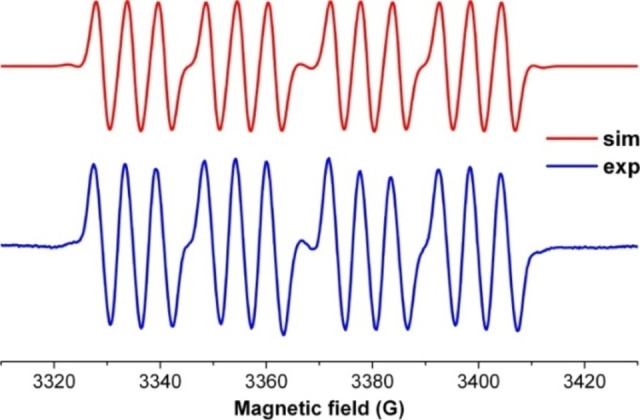
EPR spectra of compound **206** ^.−^ (simulated; red) and (experimental; blue) at 285 K. Reproduced with permission from Ref. [277]. Copyright (2015) American Chemical Society.

The radical anion **206** ^.−^ was also studied by EPR spectroscopy. The diradical was prepared in‐situ and EPR spectrum was recorded in toluene at 285 K. X‐band of its EPR spectrum exhibited 12 highly resolved and equally intense lines (Figure 24). The computationally simulated splitting pattern exhibits doublet of doublets, in which each component further split up in to three equally spaced lines, giving total 12 hyperfine lines in the spectrum. This splitting is due to the coupling of electronic spin with one ^14^N nucleus (*I*=1) at 5.9 G that falls in the typical range of cAAC radicals.^[268]^ The two larger doublet hyperfine splitting at 44.1 and 20.6 G can be assigned to two inequivalent ^31^P nuclei (*I*=1/2 ). ^31^P exhibits stronger coupling with electron spin than the ^14^N nucleus due to its larger nuclear magnetic moment.

#### 3.12.3 Carbene Centered Radicals and Radical Anions

**Scheme 64:**
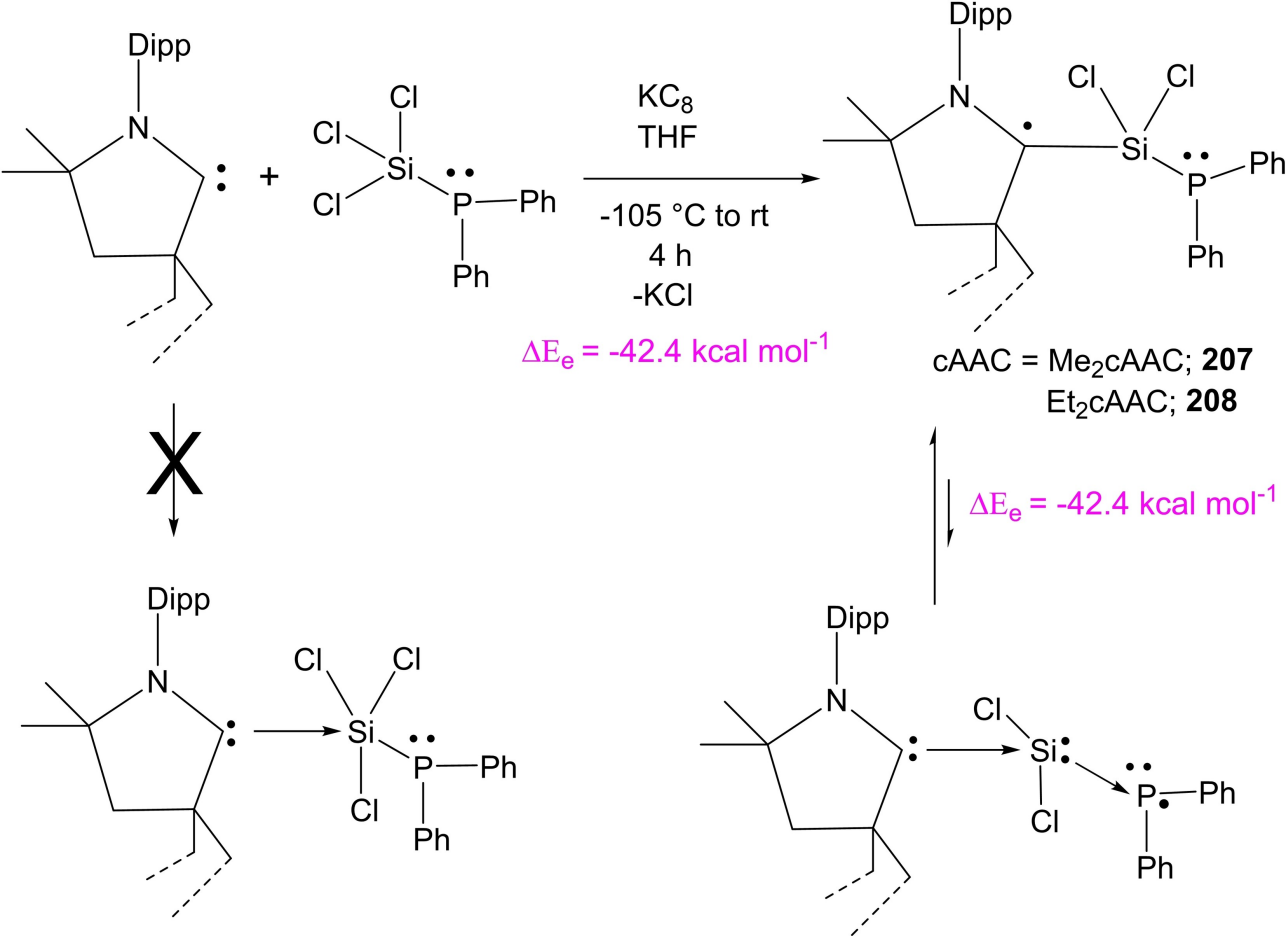
Synthesis route of Ph_2_P−Si(cAAC⋅)Cl_2_ **(207** and **208)**.

NHCs and cAACs largely differ in reactivity due to their inherent electronic properties. NHCs, unlike cAACs react with PCl_3_ to form NHC‐PCl_3_ adduct through a dative bond.^[278]^ Moreover, NHC‐PCl_3_ undergoes reduction by six equivalents of KC_8_ to form bisphosphinidene (NHC−P)_2_. Indeed, the study of the cooperative carbene affinity of silicon and phosphorus present in the same compound has not been well explored. The first synthetic route of phosphine substituted chlorosilanes was reported by du Mont et al. Recently, our group realized the importance of carbene/phosphino‐chlorosilane chemistry since it follows a significantly diverse path in comparison to carbene/SiCl_4_ and carbene/PCl_3_.^[279]^ Eventually, we reported the isolation of carbene‐stabilized diphenylphosphino‐dichlorosilane radicals with the general formula Ph_2_P−Si(cAAC ⋅ )Cl_2_ having methyl (**207**) and ethyl (**208**) substitution on cAAC component (Scheme 64). Precursor Ph_2_P−SiCl_3_ that is used in the synthesis of **207** and **208** was prepared by insertion reaction of SiCl_2_ into P−Cl bond of Ph_2_P−Cl and intermediate SiCl_2_ was generated in situ.^[280]^ Radicals **207** and **208** were formed when Ph_2_P−SiCl_3_ was reacted with cAAC in the presence of KC_8_ in a 1 : 1 : 1 molar ratio in THF at −105 °C to room temperature. The needles of **207** and **208** were isolated in a 22–25% yield. **207** and **208** were synthesized by controlling reaction conditions such as temperature and molar ratios. In order to compare the outcome, a similar reaction was carried out with the same equivalents of reactants except for NHC in place of cAAC; NHC→SiCl_2_ was isolated instead of (NHC)Si(Cl_2_)(PPh_2_).^[281]^

**Figure 25:**
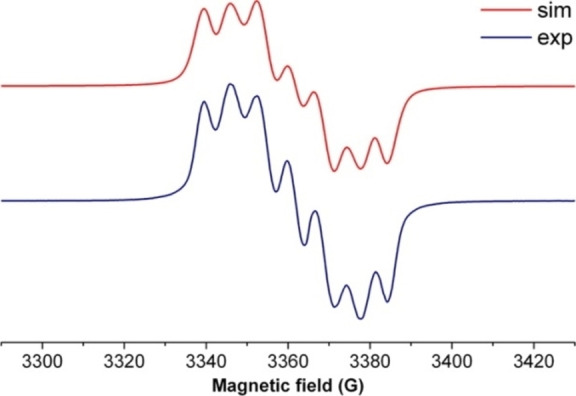
Experimental and simulated EPR spectra of **207** at 298 K. Reproduced with permission from Ref. [279]. Copyright (2015) American Chemical Society.

Radical properties of **207** and **208** were studied with EPR spectroscopy by recording X‐band EPR resonance spectra in toluene (Figure 25). **207** shows a partially resolved EPR spectrum. The experimental EPR spectrum matches with theoretically calculated data having hyperfine coupling: a (^31^P) 15.6 G (calcd 20.5 G), a (^14^N) 6.5 G (calcd 4.2 G), a (^35^Cl) 4.1 G (calcd 3.1 G). The spin density is mostly located at trivalent carbon of cAAC (ca.75%); the adjacent nitrogen atom accommodates approximately 19% spin density. Accordingly, the g factors for both **207** and **208** are 2.0027 and 2.0024, respectively that are very close to g factor of free electron (2.0023).

Although, the heteroatom ^31^P and one of the Cl atoms in β‐position account for small spin densities (<5%) but they show detectable hyperfine coupling. A ^29^Si satellite coupling (4.7% nat. abundance, *I*=1/2) was observed for **208** at about 10 G (calcd 13.4 G; 3.6% spin density). The EPR spectra of **208** at temperatures between 183–340 K give variable line widths, indicating that an apparently mobile ethyl substituent of low symmetry is present.

**Figure 26:**
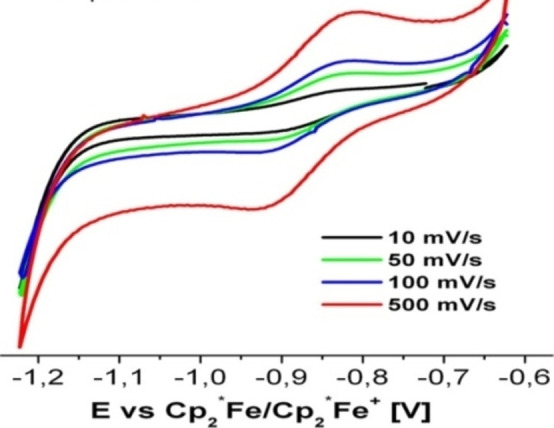
Cyclic voltammogram of **207** recorded in THF solvent at various scan rates, containing 0.1 M [n‐Bu_4_N]ClO_4_ as an electrolyte. Reproduced with permission from Ref. [279]. Copyright (2017) American Chemical Society.

Cyclic voltammetry (CV) is the most frequently used method for the study of redox properties. Redox properties of **207** were investigated by cyclic voltammetry that shows a one‐electron quasi‐reversible process at E_1/2_=−0.96 V against Cp*_2_Fe/Cp*_2_Fe^+^, indicating the formation of the anion (**207^−^**) (Figure 26). The calculated one‐electron ionization energy and electron affinity of **207** are 5.3 eV (122.4 kcal/mol; **207** ^+^) and 1.1 eV (26.3 kcal/mol; **207^−^**), respectively.

#### 3.12.4 cAAC Stabilized Boryl Radicals

**Scheme 65:**
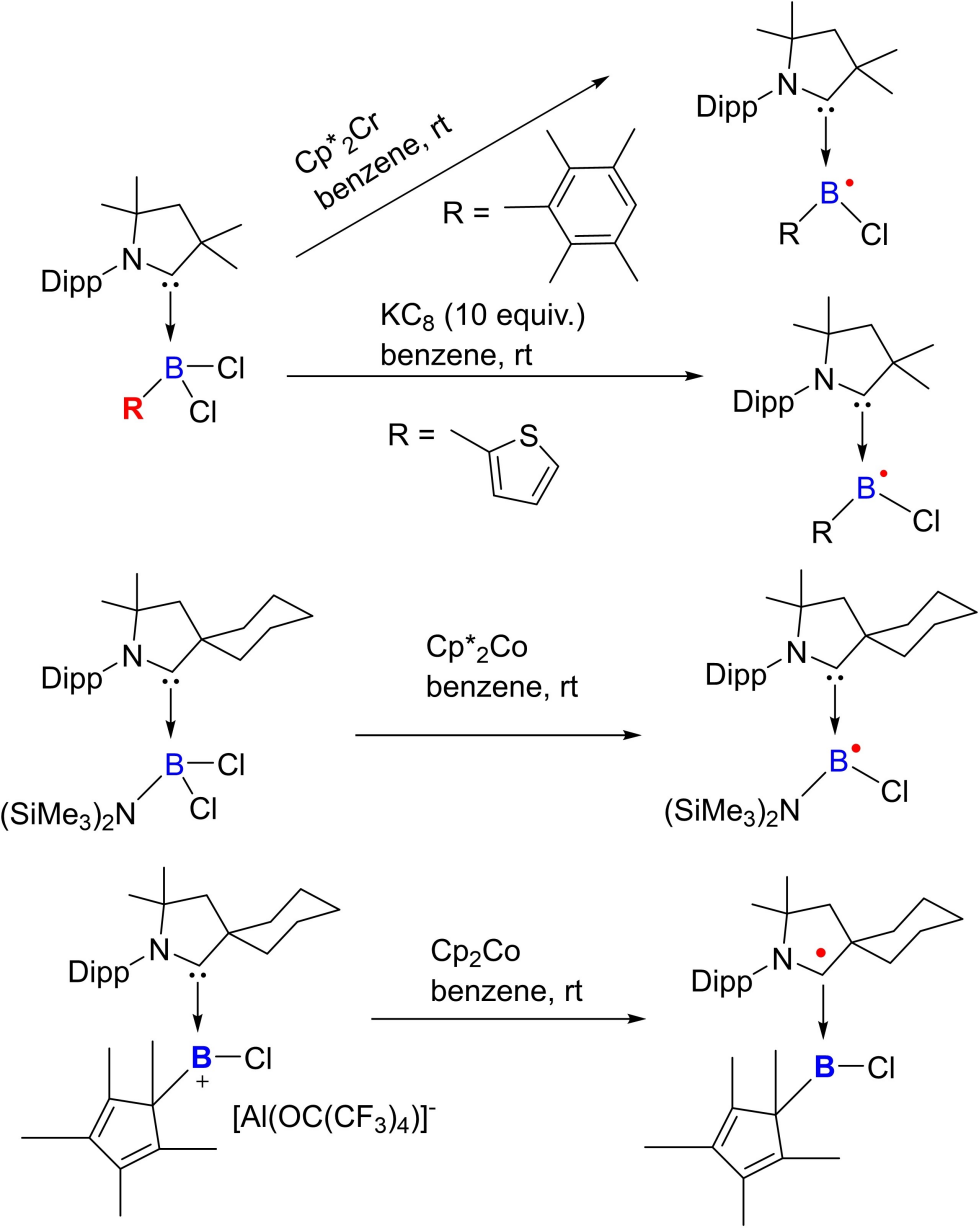
Preparation of cAAC ligated boryl radicals.

**Scheme 66:**
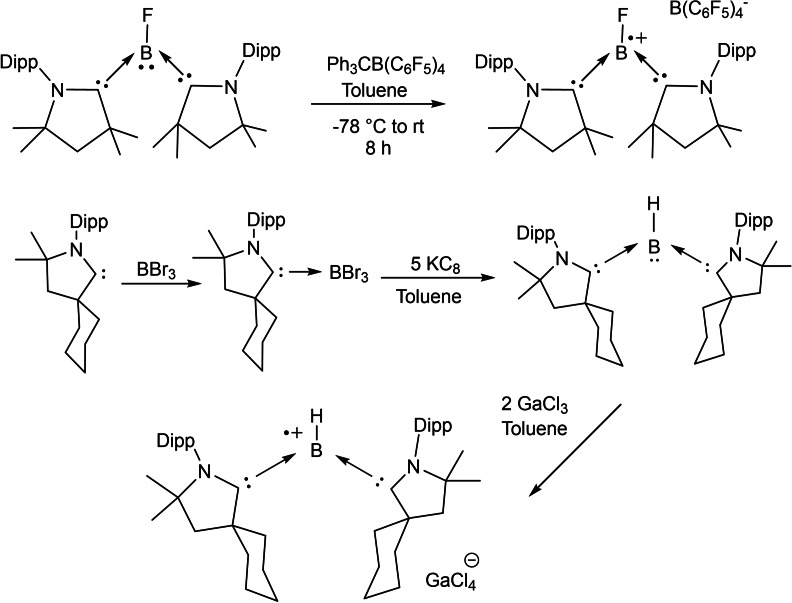
Preparation of boron‐based radical cations.

Theubiquitous involvement of radicals in several chemical and biological processes has made them unique species in terms of reactivity. Boron atom which bears an empty p‐orbital is inherently known to form electron‐deficient compounds. The empty p‐orbital can be populated by electron donations of an electron pair from a Lewis base or by a chemical reaction. However, the addition of a single electron to the empty p‐orbital is very challenging and results in the generation of highly reactive radical species. Thankfully, recently radical chemistry of boron has seen tremendous developments and several stable boron‐based cAAC radicals and radical cations have been stabilized (Scheme 65 and 66).[^15^, ^17^, ^118^, ^121^, ^136^, ^282^, ^283^, ^284^]

**Scheme 67:**
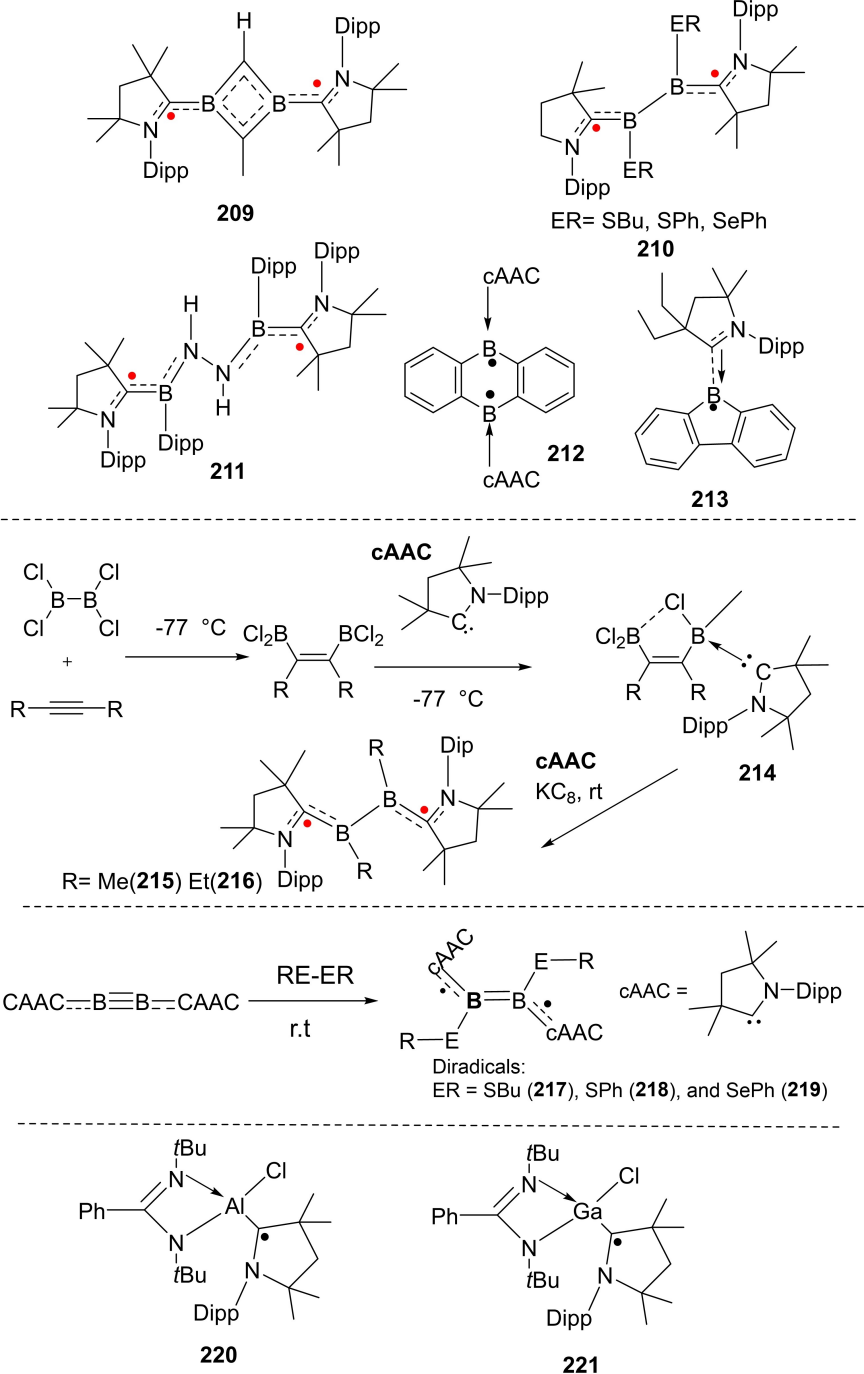
Dianionic and neutral boron‐based diradicals.

cAACs have been increasingly used as neutral donor atoms in the stabilization of the main group, transition metal, and organic radical species. However, neutral boron‐based triplet diradical species were unknown until Braunschweig et al. isolated such species featuring two cAAC stabilized boron centers (**209**, **210**, **211**).[^285^, ^286^] These diradical species were prepared by oxidation reactions of diborynes^[285]^ or through the protonation of a dianionic product of N_2_ fixation by borylene species.^[286]^ Recently, Braunschweig et al. prepared unusual halide bridged, cAAC stabilized diborylalkenes (**214**) which on reduction give unusual radical species spanned by two‐carbon‐atom‐bridges (**215** and **216**).^[287]^ These compounds behave as diradicals in which radical centers are weakly coupled. Such neutral boron‐based diradicals have been rarely reported (Scheme 67). Braunschweig and co‐workers have also reported the isolation of diborenes and their 90°‐twisted diradical congeners (**217**, **218**, **219**) (Scheme 67, bottom).^[285]^ They have also reported the isolation of 9,10‐diboraanthracenes (**212**) with open‐shell singlet biradical character in the ground state, which was only theoretically predicted until now.^[288]^ The introduction of boron atoms in the anthracene lowers HOMO‐LUMO gap, which makes it possible to isolate such singlet open‐shell biradical species. Recently, Yang et al. have also reported cAAC‐stabilized borafluorene radical (**213**).^[289]^ The heavier congeners of boron radical species stabilized by cAAC ligands have also been reported. Recently, Siddiqui et al. and Banerjee et al. isolated cAAC stabilized radical species of aluminum and gallium (**220** and **221**).[^290^, ^291^] The synthesis of **221** is the first report of a neutral gallium radical stabilized by a carbene ligand. Both radicals are stable at room temperature in solution as well as a solid state, for several days.

Boron‐based biradical compounds possess interesting properties controlled by steric and electronic factors. For instance, cAAC stabilized boron moieties having a C_2_ bridge are closed‐shell species in the ground state; because it allows π ‐electron delocalization. However, when alkyl‐substituted C_2_R_2_ bridge is present in between B−cAAC moieties, the molecule adopts a twisted geometry and the molecule results in an open‐shell singlet ground state. Apart from substitution on C_2_ bridge, the substitution on cAAC moiety also controls the molecular twisting. These factors have been computationally studied.^[292]^

**Figure 27:**
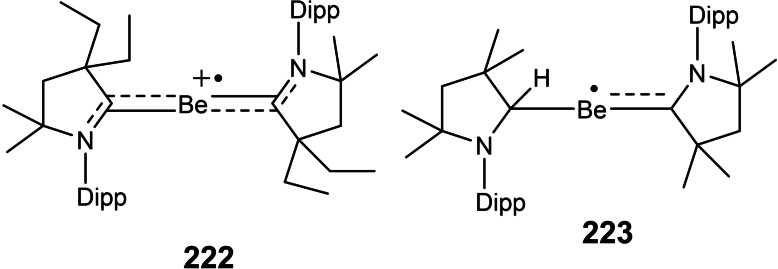
cAAC – stabilized beryllium radical cation and neutral Be^I^ radical.

Transition metals and p‐block elements dominate cAAC chemistry. There are very few reports of alkaline earth metals being stabilized by cAAC ligands. These metals, in general, form diamagnetic compounds with a +2 oxidation state. However, recently, some progress in cAAC chemistry of s‐block elements has been seen. Wang et al. isolated a beryllium radical cation species (**222**) which is stabilized by cAAC ligands.^[293]^ This represents the first paramagnetic beryllium complex as a radical cation. Additionally, neutral Be^I^ radical (**223**) has also been stabilized by cAAC (Figure 27).^[294]^ The Be^I^ radical complex was synthesized by reduction of organoberyllium chloride and structure was confirmed by single‐crystal XRD and EPR spectroscopy.

### 3.13 cAACs Stabilized Single‐Molecule Magnets (SMMs)

**Figure 28:**
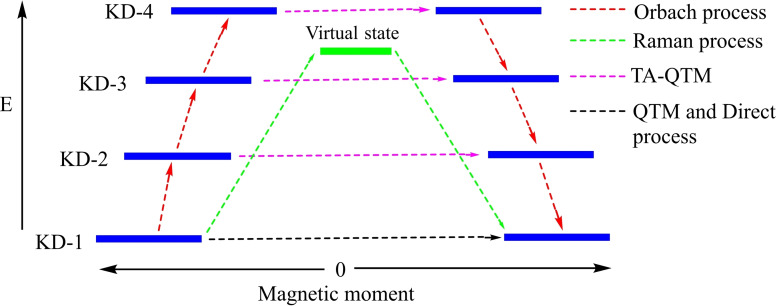
Possible relaxation pathways/mechanism in a hypothetical lanthanide‐based SMM consisting of four Kramer‘s doublets. Reproduced with permission from Ref. [295]. Copyright (2019) American Chemical Society.

Single‐molecule magnets (SMMs) are metal‐organic compounds that are magnetized due to spin orientation in a magnetic field and exhibit slow relaxation when the magnetic field is switched off. The large energy barrier (U_eff_) between the spin‐up and the spin‐down states is responsible for the slow magnetic relaxation. The relaxation of magnetization/spin reversal can follow different paths depending upon metal ion, ligand system, and geometry of the complex. The magnetic relaxation in transition metal‐based SMMs is largely different than lanthanide ions‐based SMMs. In particular, lanthanide ions exhibit large spin‐orbit coupling that yields not only large magnetic anisotropy but also allows mixing of m_J_ levels that leads to the quantum tunneling mechanism (QTM) between the ground or first excited Kramers doublet (KD). Other than QTM, magnetic relaxation pathways observed in SMMs are direct pathways, Raman and Orbach process (Figure 28).^[295]^ QTM greatly reduces energy barrier at lower temperatures hence fastening the relaxation process that is not a desirable process for efficient SMMs; therefore, quenching of QTM is administered by modifying the metal skeleton of the clusters and ligands system. One of the prevalently used strategies to quench QTM is to replace some of the lanthanide ions with transition metal ions. In recent years, several lanthanoid ions based SMM/SIM with low‐coordination ligands have been synthesized. Such SMM/SIMs have been made by taking the advantage of anisotropic electron density distribution around lanthanoid ions and using suitable ligands which increase axial anisotropy. Basically, lanthanoid ions such as Dy^3+^, Ce^3+^, Ho^3+^, Nd^3+^, Tb^3+^ have pronounced oblate character in terms of electron density distribution, which means that most of the electron density is in equatorial xy‐plane rather than in axial z‐plane. Thus, when we use a ligand that coordinates to lanthanoid ion from the axial direction, it minimizes electronic repulsion. As a result, the probability of getting SMMs with higher energy barriers and blocking temperature increases. As per our literature survey, there is no report of cAAC‐stabilized lanthanoid ion‐based SMMs.

As discussed above, SMMs are characterized by a very high spin state and a large magnetic anisotropy. Transition metal ions (TMIs) have several unpaired electrons hence they possess a high spin state; on the other hand, lanthanide ions show slow magnetic relaxation due to large magnetic anisotropy. Due to the presence of such favorable factors, TMIs and lanthanide ions have been frequently utilized to construct several mixed metallic SMMs. Other important parameters that are important in designing good SMMs is blocking temperature (T_b_), the temperature at which magnetization gets blocked (zero magnetic moments). The higher blocking temperature for SMMs is desirable from point of view of the technological utility of SMMs. The highest blocking temperature achieved to date in any SMMs is 80 K which was reported by Guo et al.[^296^, ^297^, ^298^] in [(Cp^ttt^)_2_Dy]^+^ cation. It is important to note that complex geometry and coordination numbers also affect the efficiency of SMMs. The linear two coordinated complexes of lanthanides possess very high uniaxial symmetry; therefore energy gap between the ground state and the first excited state doublets will be the largest.^[299]^ The observations reveal that the construction of stable linear Ln^III^ complexes is synthetically challenging compared to transition metal analogs, therefore such complexes are rarely reported.

**Scheme 68:**
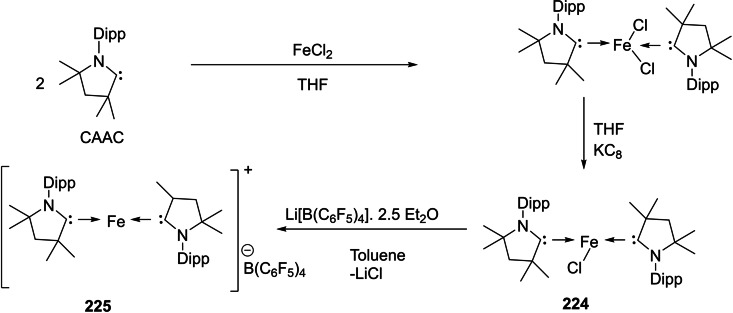
Synthesis of cAAC stabilized single‐molecule magnets, **224** and **225**_._

**Figure 29:**
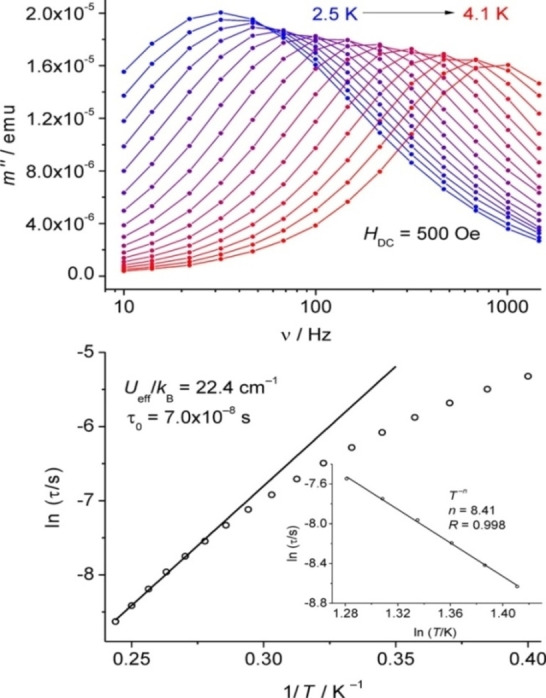
Frequency versus imaginary part of the ac susceptibility under the applied field of 500 Oe for **170** (top). Arrhenius plot of the temperature versus relaxation time τ (bottom). The black line shows a thermally activated relaxation. Inset: power‐law analysis in the form ln(τ) vs ln(T). Reproduced with permission from Ref. [75]. Copyright (2016) American Chemical Society.

In today's technological world, data processing and storage have become an important component of devices and the development of compact storage devices with high data storage capacity. In this connection, SMMs are potential candidates for such developments. Recently, SMMs have found vast applications in different fields including information processing, data storage, spintronics, quantum computing, magnetic refrigeration, and biomedical applications. Several ligand systems including Schiff‐bases have been used to prepare SMMs of different metal ions but the use of stable carbenes to design SMMs has not been so common. Recently, few cAAC‐stabilized single‐molecule magnets (SIMs) have been reported. For instance, we have reported tricoordinate [(cAAC)_2_Fe(I)Cl], **224** and bicoordinate [(cAAC)_2_Fe]^+^[B(C_6_F_5_)_4_],^−^ **225** cAAC stabilized iron complexes (Scheme 68).^[300]^ The complex **225** is the first species in which Fe(I) is stabilized in a three‐coordinate non‐chelating coordination environment and **171** is the first cationic complex with a bicoordinate Fe(I) center.^[300]^ Both complexes **224** and **225** show slow magnetic relaxation under the applied dc magnetic field which is behavior typical to the SMMs. The complex **170** exhibits slow magnetic relaxation under the applied dc field of 500 Oe (Figure 29, top). This indicates the occurrence of fast QTM under zero applied field which is negated on applying 500 Oe dc magnetic field. It is apparent from the Arrhenius plot. Moreover, slow magnetic relaxation is thermally possible at higher temperatures (Figure 29, bottom). A linear fit using τ=τ_0_ exp(U_eff_/kBT) gives an energy barrier of U_eff_/k_B_=22.4 cm^−1^ with τ_0_=7.0×10^−8^ s which indicates that **224** is an SMM. Complex **225** with two‐coordinate Fe^I^ also exhibits a detectable out‐of‐phase signal suggesting that it might be a single‐ion magnet (U_eff_<20 cm^−1^).^[75]^ Mössbauer spectroscopy reveals the +1 oxidation state and S=3/2 spin ground state of iron in the compounds **224** and **225**.

**Figure 30:**
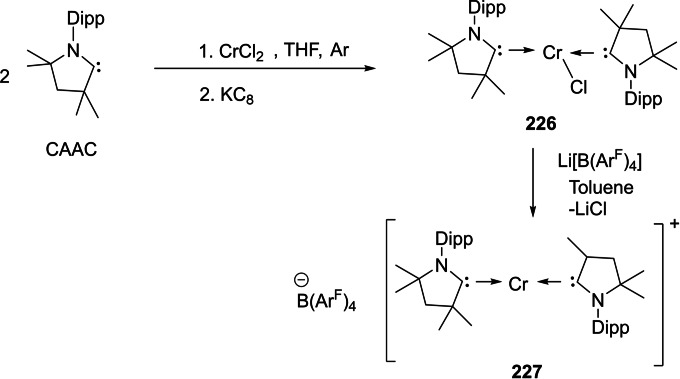
Structure of **226** and **227**.

**Figure 31:**
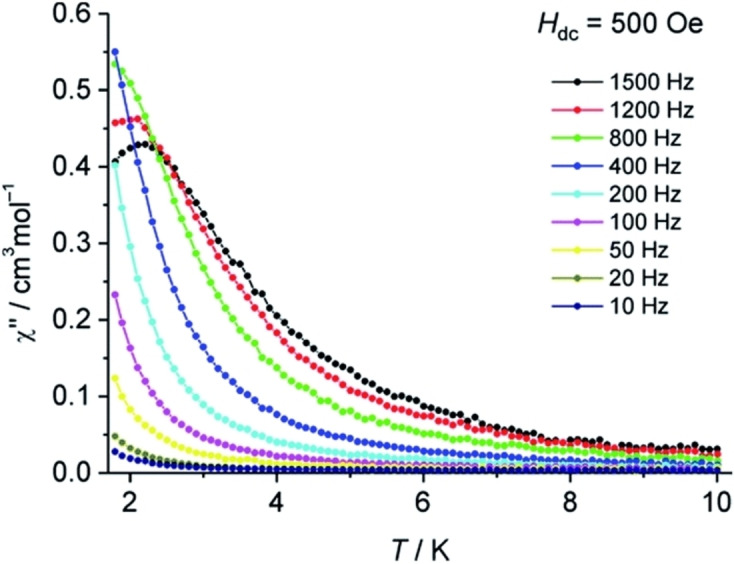
Temperature versus χ′′ for (Me_2_−cAAC)_2_Cr^I^Cl at various frequencies at 500 Oe of applied dc field. Reproduced with permission from Ref. [75]. Copyright (2016) American Chemical Society.

The high‐frequency EPR measurements confirm the 3/2 spin ground state with large, positive zero‐field splitting (20.4 cm^−1^)^[300]^ and reveal zero plane anisotropy for compound **224** (Scheme 68). (Me_2_−cAAC)_2_Cr^I^Cl (**226**) and [(Me_2_−cAAC)_2_Cr]^+^(BAr^F^_4_)^−^] (**227**) (Figure 30) are the other set of cAAC stabilized complexes with spin ground state S=5/2 which show slow magnetic relaxation under an applied dc magnetic field of 500 Oe hence SMMs behavior is observed.^[75]^ The application of dc fields suggests frequency dependence in the imaginary part of magnetic susceptibility (*χ*′′) thus suggesting slow magnetic relaxation in **226** at lower temperatures (Figure 31).

### 3.14 cAAC Supported Multinuclear Clusters

**Figure 32:**
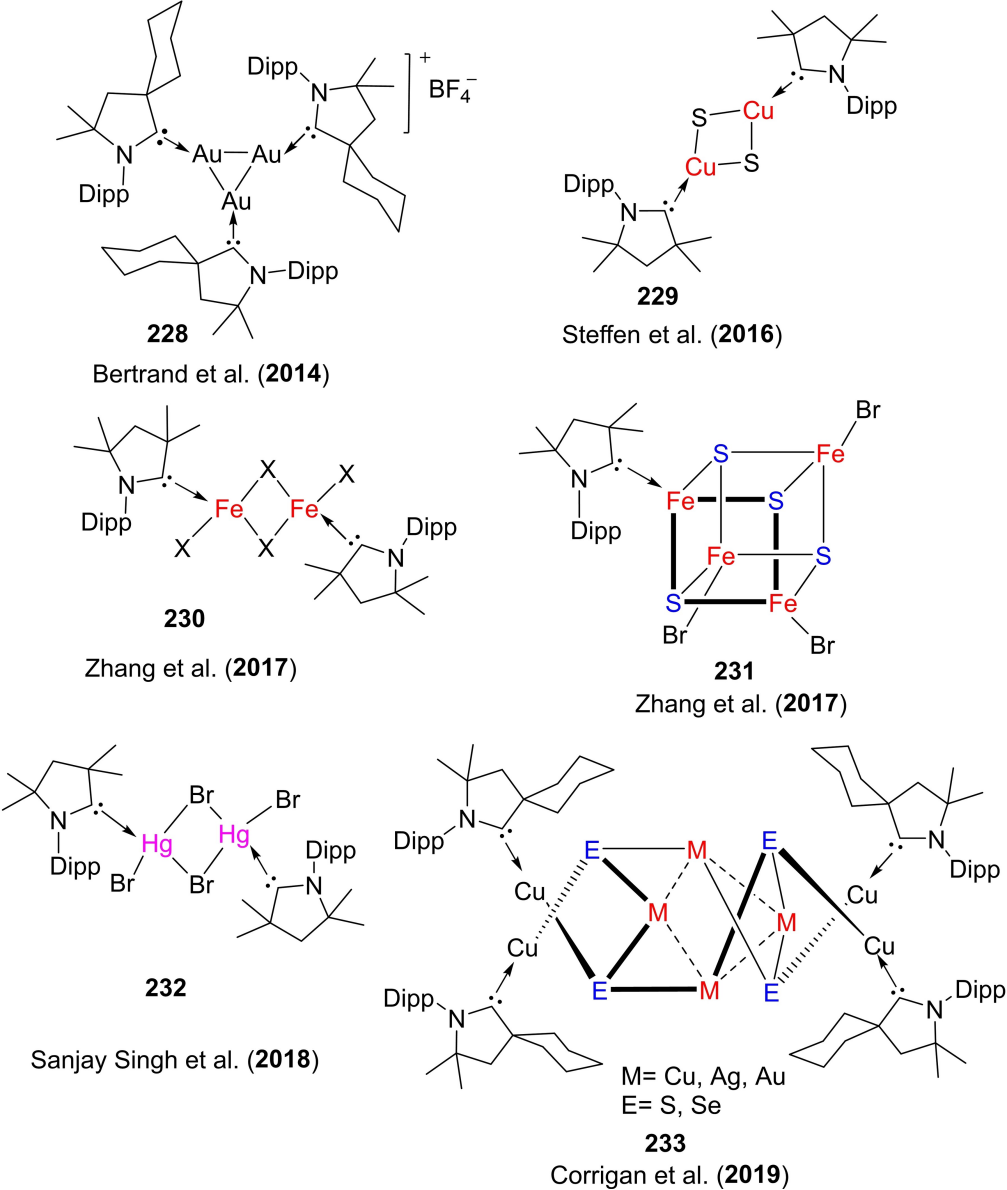
cAAC stabilized multinuclear clusters.

Multinuclear clusters of transition metals stabilized by different ligand systems have been reported in multitude; however, cAAC stabilizes multinuclear clusters have not been so common. The most of metal complexes formed by cAAC stabilization are mono‐metallic. Bertrand and co‐worker isolated an air‐ and moisture‐stable trinuclear mixed‐valence gold(I)/gold(0) cluster, Au_3_(cAAC)_3_ (**228**).^[301]^ These gold complexes act as mimics of gold heterogeneous catalysts. Recently cAAC stabilized dinuclear complexes of copper, iron, and mercury have been reported. Steffen et al.^[220]^ have isolated and investigated a series of copper (I)‐carbene complexes including a dimeric copper complex (**229**) which exhibits surprising photophysical properties. Recently, Zhang et al.^[302]^ have synthesized thiolate‐bridged diiron(II) complexes (**230**) containing Me_2_−cAAC ligands. Isolation of these unprecedented carbene complexes was made possible by the combined utilization of Me_2_−cAAC and thiolates. The coordination environment of each tetrahedral Fe(II) consists of one terminal bromide ion, one carbene carbon atom, and two thiolate sulfur atoms, which is similar to the carbide‐containing sulfur‐rich environment of Fe centers in the belt region of the FeMo‐cofactor. An interesting observation revealed that NaSCPh_3_ acting as the thiolate ligand, homolytic cleavage of C−S bond gives a rare [3 : 1] site‐differentiated cubane‐type cluster [(Me_2_−cAAC)Fe_4_S_4_(Br)_3_][Me_2_−cAACH] (**231**) (Figure 32).^[302]^ Singh et al. have reported the first isolation of cAAC stabilized dinuclear mercury (II) clusters (**232**) (Figure 32). This cAAC stabilized Hg(II) has been demonstrated to exhibit catalytic utility in intermolecular hydroamination of phenylacetylene with aniline.^[50]^ Corrigan et al. synthesized a series of phosphorescent homo‐ and heterometallic copper(I)‐chalcogenide clusters stabilized by cAAC‐ligands [Cu_4_M_4_(μ_3_‐E)_4_(cAAC^Cy^)_4_] (M=Cu, Ag, Au; E=S, Se) (**233**) by the reaction of the new copper(I) trimethylsilylchalcogenolate compounds [(cAAC^Cy^)CuESiMe_3_] with ligand‐supported group 11 acetates.^[303]^

### 3.15 Polymerization Reactions by cAAC‐Containing Compounds

**Scheme 69:**
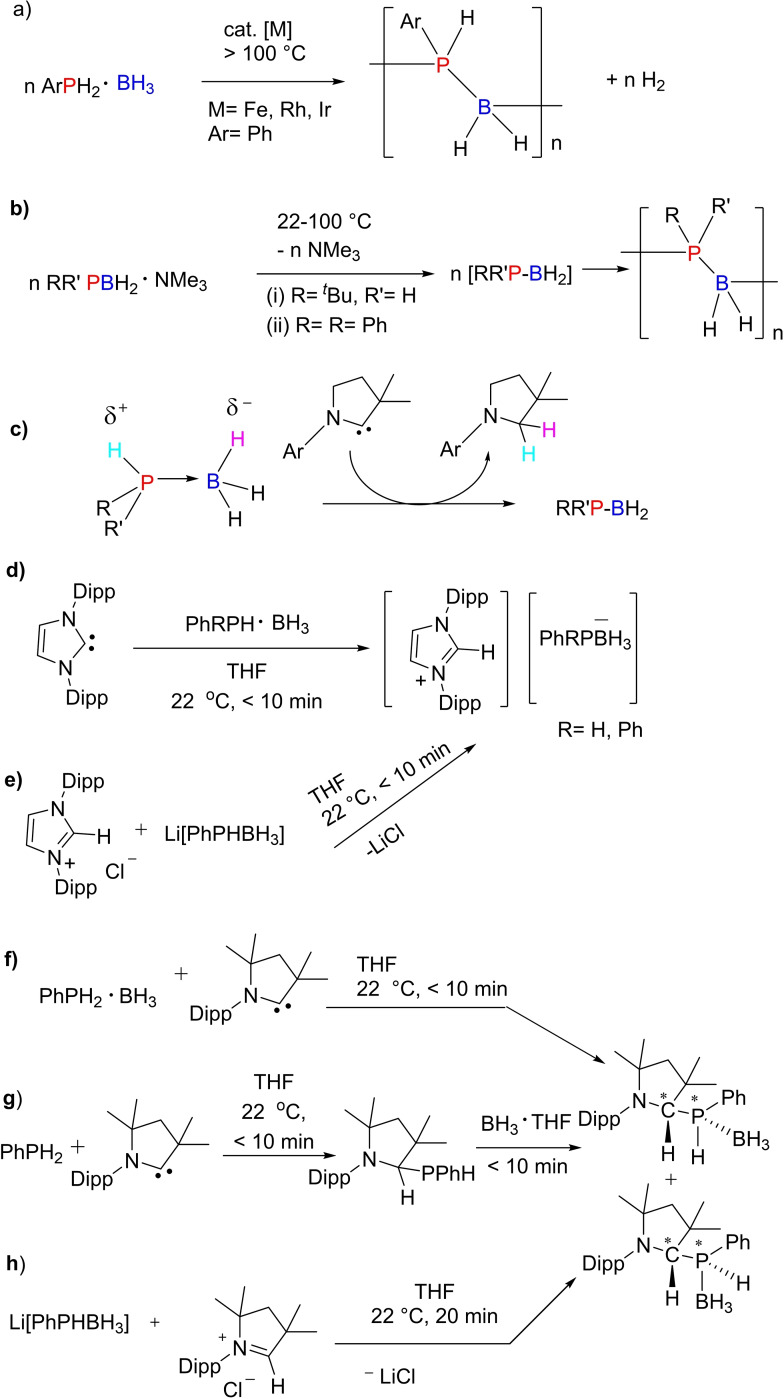
Metal and cAAC‐mediate synthesis of polyphosphinoboranes.

The polymers, which contain p‐block elements such as phosphorus and boron in the main chain, constitute an important class of materials due to their potential applications in making ceramic precursors, elastomers, polyelectrolytes, and optoelectronics.[^304^, ^305^] The metal‐catalyzed coupling pathways have been reported to access a broad range of inorganic polymers and materials. Especially, catalytic dehydrocoupling between main‐group substrates is an important method for the formation of E−E′ bonds. This method can also be utilized to prepare polymers via catalytic dehydropolymerisation **(**Scheme 69a).^[306]^ However, metal‐catalyzed protocols require harsh reaction conditions; therefore, a benign catalyst system is needed. Recently, some metal‐free polymerizations by NHCs and cAACs have been reported. Scheer and co‐workers^[307]^ have reported a metal‐free synthesis of polyphosphinoboranes by thermal decomposition of amine‐stabilized phosphinoboranes, RR′PBH_2_ ⋅ NMe_3_, which proceeds under milder conditions 22–40 °C. This synthetic route enables synthesis of high‐molecular‐weight poly‐tert‐butylphosphinoborane, [^t^BuHPBH_2_]_n_, presumably via the monomeric phosphinoborane [^t^BuHPBH_2_] (Scheme 69b). The divalent carbene carbon center in cAACs is known to mimic transition metals in terms of insertion into E−H σ‐bonds (E=H, N, Si, B, P, C, O) with the formation of new, strong C−E and C−H bonds. The transition metal‐like insertion by cAACs is attributed to the small HOMO‐LUMO gap in them. However, one of the limiting drawbacks is that the resulting C(sp^3^)−E and C(sp^3^)−H σ‐bonds are stronger which does not favor further reactivity of the H−C(sp^3^)−E products. Therefore, cAACs cannot behave like transition‐metal centers in synthetic utility. Manners et al.^[308]^ anticipated that a cAAC‐mediated dehydrogenation of primary and secondary phosphine‐boranes, species that contain both protic P−H and hydridic B−H bonds, might be possible (Scheme 69c). Dehydrogenation of phosphine‐boranes utilizing this strategy gives reactive phosphinoborane monomers, with appropriate substituents at the phosphorus and boron centers. The resulting polymers give soluble oligomeric and polymeric material. Manners et al. have also investigated the dehydrogenation potential of NHCs as well as cAACs with phosphine‐borane compounds. They have shown that cAACs mediate metal‐free dehydropolymerisation of phosphine‐boranes (Scheme 69).^[308]^

### 3.16 Crystallochromism in cAAC‐Complexes

**Scheme 70:**
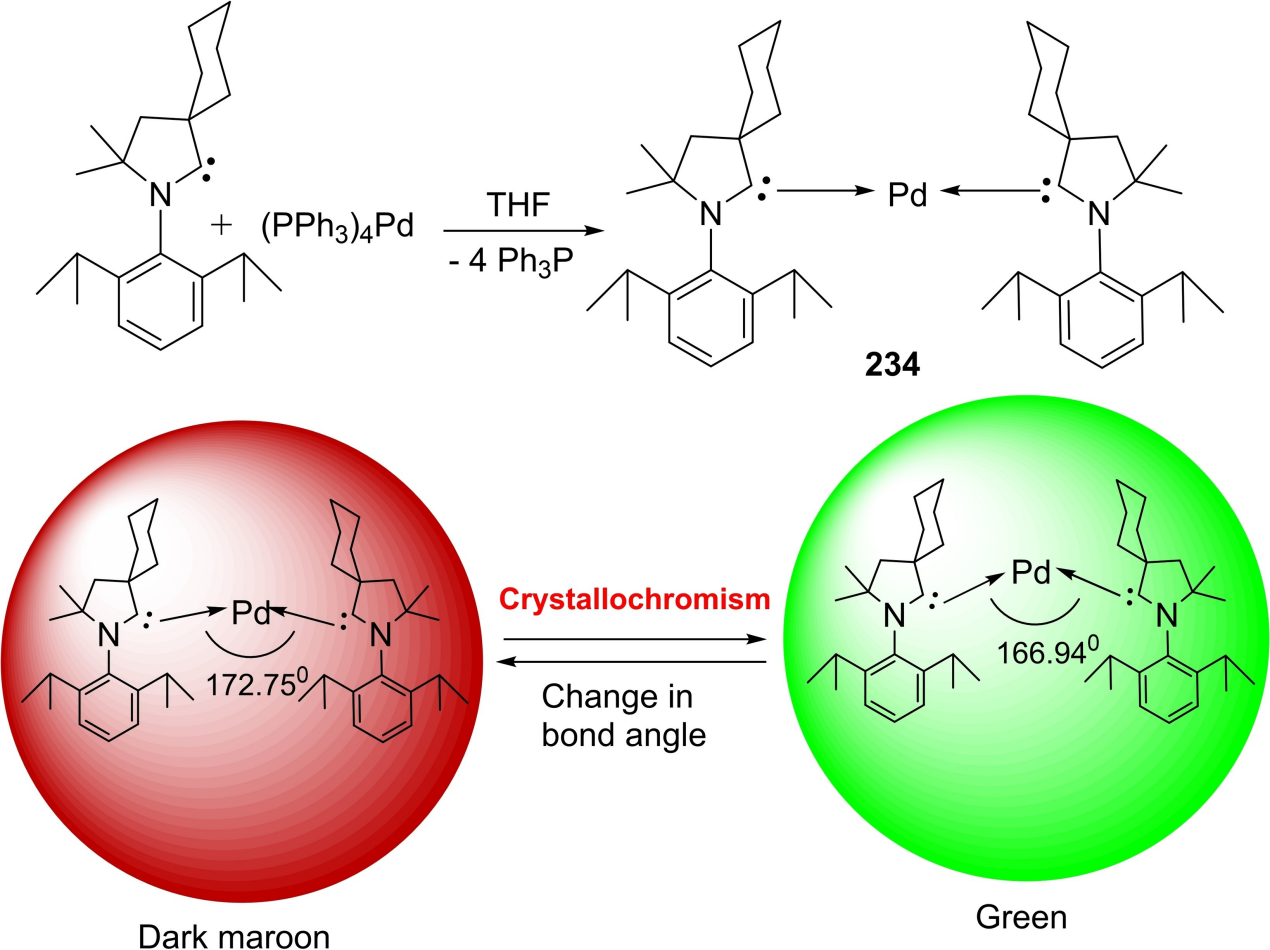
Synthesis of [(cy−cAAC)_2_Pd] (**234**) and (bottom) crystallochromism in **234**.

Chromism is a process that induces a color change in chemical compounds on the application of external stimuli such as heat, pressure, the polarity of solvent etc. The color change is witnessed due to changes in the electron states of molecules. The chromism phenomenon has been used in the commercial production of optical switches, sensors, optical memory, direct thermal printing etc. Based upon external stimuli, chromism has been classified into different types such as mechanochromism, solvatochromism, thermochromism, crystallochromism etc. The crystallochromism is one such type in which color change is observed due to alternation in the crystal structure of the molecule. In most cases, a change in bond angle triggers crystallochromism. Crystal packing during co‐aggregation of molecules is influenced by even a small change in molecular structure. The change in bond angles and bond lengths can influence the packing and hence crystal color may change leading to the observance of crystallochromism. As we discussed, crystallochromism is a phenomenon of change in color of a chromophore on changing its crystal structure^[309]^ and it is observed in innumerable organic chromophores. Though, crystallochromism in palladium complex of N‐heterocyclic (NHC), for example in [(NHC)_2_Pd],^[310]^ has been observed. However, the crystallochromism was not reported in cAAC complexes until we observed it in 2015. We reported crystallochromism in palladium‐cAAC complexes in 2015 for the first time.^[311]^ We synthesized [(cAAC)_2_Pd] complex (**234** and **234**⋅THF) which exhibits crystallochromism by changing color from dark maroon to bright green due to bending of the C−Pd−C bond angle from 172.75(6)8 to 166.94(6)8. **234** was synthesized by dissolving Cy−cAAC and [(Ph_3_P)_4_Pd] in 11 : 2 molar ratio in THF, forming a dark green solution of [(Cy−cAAC)_2_Pd], which was stirred at room temperature for 1 h then 20 min at 70 °C and finally 12 h at room temperature. The dark maroon crystals of rod‐shaped were isolated from green solution at −32 °C. The Yield of isolated crystals (**234** ⋅ THF) was calculated to be 84%. The crystal **234** ⋅ THF was characterized by single‐crystal XRD by carefully mounting crystal under inert atmosphere at liquid nitrogen temperature. On separating the crystals of **234** ⋅ THF by filtration, the maroon rods quickly turn to bright green powder indicating loss of solvent molecule (THF) from the lattice. To know the structure of these small green crystals, a single‐crystal X‐ray diffraction experiment was performed. The XRD measurement proved that **234** has exactly the same crystal structure, except coordinated THF molecule. This observation suggests that the interfering solvent molecule present in the crystal lattice may cause structural changes. The energy needed to reduce the size of the C−Pd−C angle from 172 to 166° was computationally calculated to be only 0.5 kcal/mol. When solvent was changed from THF to non‐coordinating solvents such as toluene or benzene, it does not give a different color, suggesting that this color change is due to slight changes in the structural parameters of [(cAAC)_2_Pd] like bond angle or bond length. This crystallochromism/vapochromism effect is clearly resolved in **234**/**234** ⋅ THF (Scheme 70).

### 3.17 Surface Activation of Materials

Most of the materials derive applications due to their surface functionality which is achieved by precisely controlled modification of surfaces. Therefore, more universal surface anchors are required that facilitate precise control over surface functionality/activation. In this regard, one of the most demanding surfaces is the silicon surface, which has been the foundation of the present‐day semiconductor industry. As we know semiconductors constitute an integral part of modern‐day devices, therefore, controlled surface modification is an important aspect. Silicon surfaces, and their defects, in particular, play an important role in charge recombination, which is an undesirable process that leads to reduced device performance. To reduce surface recombination velocity, freshly prepared Si−H(111) surfaces are employed. The surface properties are of paramount importance since the performance of the fabricated devices is dominated by semiconductor surfaces. Although, there are several routes to render the surface inactive, however, surface passivation routes via formation of Si−C bonds^[312]^ or Si−H/heteroatom bonds^[313]^ has addressed the problem of charge recombination to some extent, however, the future advancement of silicon‐based technologies can only be achieved by developing new methods for the precise and controlled modifications of surface functionality.

**Scheme 71:**
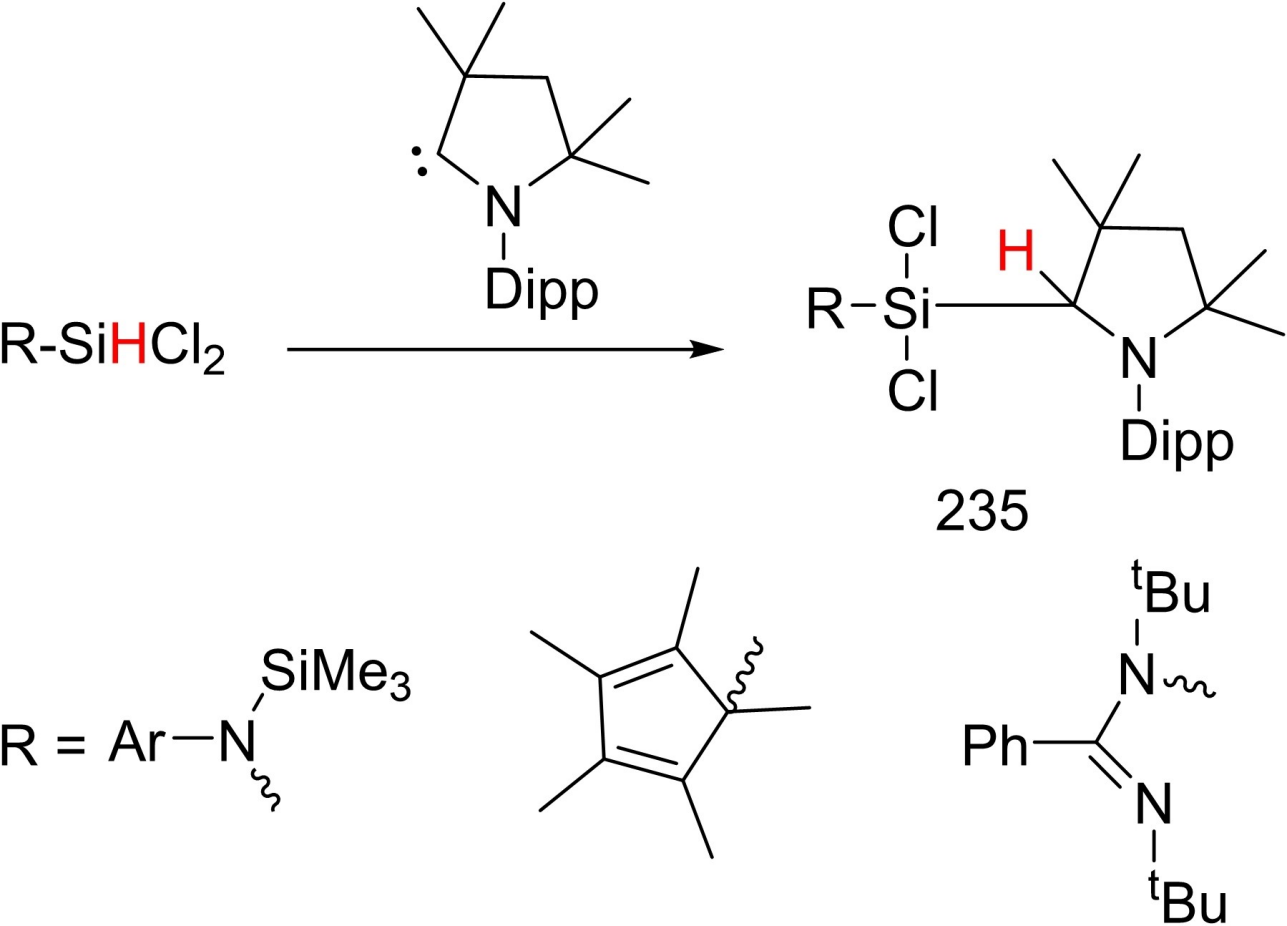
Synthesis of RSiCl_2_(cAACH), **235** by Si−H insertion reaction.

Johnson et al. reported^[314]^ the application of persistent aminocarbenes to functionalize hydrogen‐terminated silicon surfaces via Si−H insertion reactions. They demonstrated that cAACs and acyclic diaminocarbene(ADAC) can undergo insertion into Si−H bonds at the silicon surface, forming persistent C−Si bonds that provide amine or aminal functionality in the proximity to the silicon surface. Silicon surface passivation renders the surface less active by removing charge accumulation from it and making the surface interface more suitable for semiconductor materials. The passivation reaction can be carried out using compounds that form persistent bonds with silicon surface and provide inactive surface proximities. Johnson et al. carried out passivation reactions using model compounds (H−Si(TMS)_3_ and H−Si(OTMS)_3_), nanoparticles (H−SiNPs), and planar Si(111) wafers (H−Si(111)). For surface activation reaction, clean insertion under mild conditions is highly desirable. In this regard, cAACs exhibit notably clean insertion under mild conditions that produce monolayers with 21±3% coverage of Si(111) atop sites, which corresponds to the expected maximum value of 20%.[^90^, ^315^] Additionally, we^[316]^ have also demonstrated Si−H bond activation by insertion of cAACs (Scheme 71). They succeeded in selectively activating Si−H bond of hydrochlorosilanes (RSiHCl_2_) by inserting carbene carbon into the Si−H bonds (**235**).^[316]^ Since selective activation of the Si−H bond is difficult compared to the transition metals because carbenes tend to react with HCl and form adducts, this has been a notable success in silicon surface modification.

**Scheme 72:**
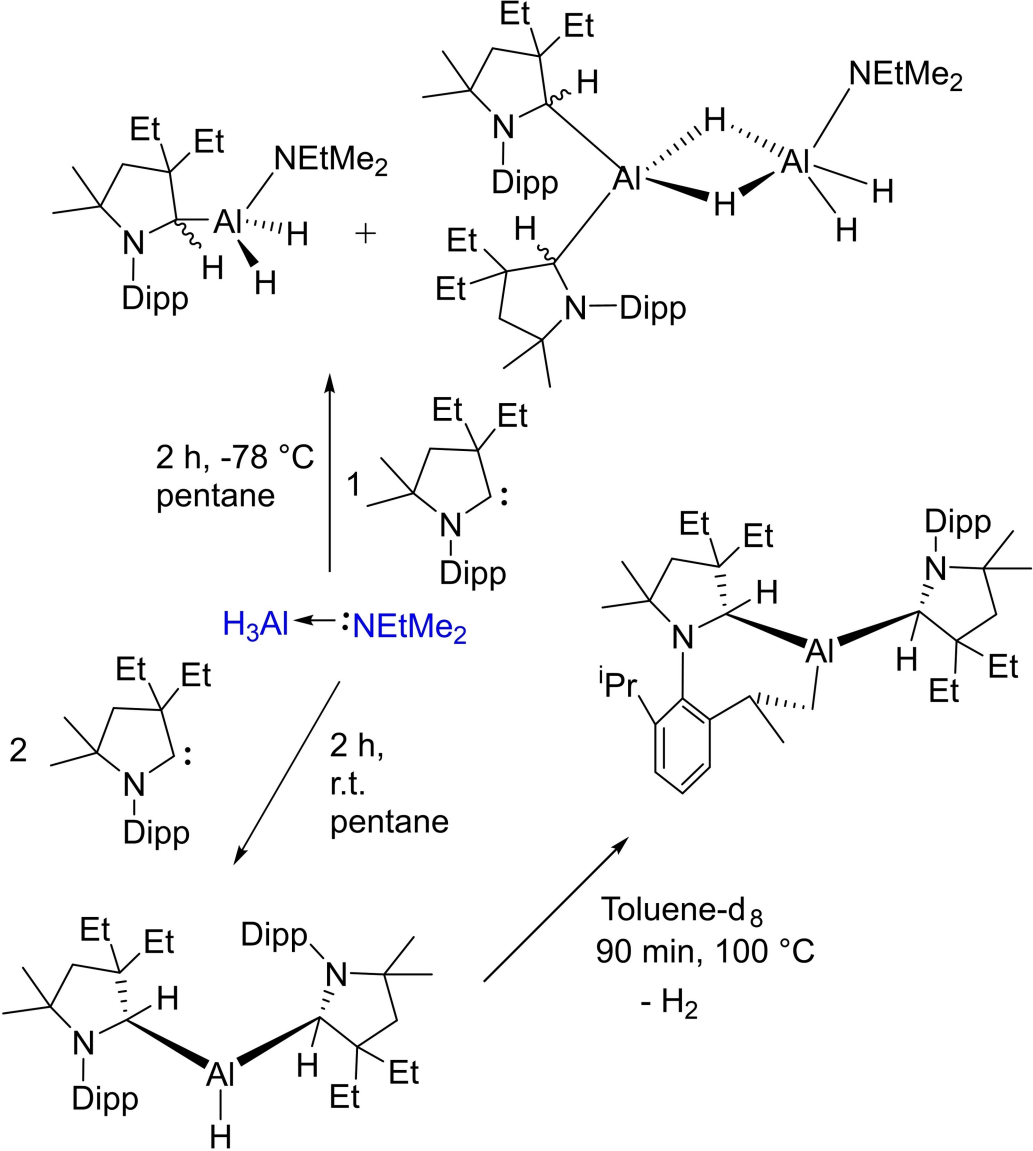
Isolation of Al−H complexes which can be activated for surface‐based applications.

Taking the analogy of NHC‐alane adducts,^[317]^ Stephan et al.^[318]^ investigated reactions of a cAAC and alane. The reaction of the cAAC^Et^ with an equimolar portion of AlH_3_‐NEtMe_2_ in pentane at room temperature was carried out (Scheme 72). The formation of the complex was also confirmed by different NMR techniques at different temperatures. The probe on resulting species reveals a rare case in which the hydride transfer from Al to the cAAC‐carbon is reversible, illustrating an unusual case of formal redox chemistry at the cAAC carbon. The cAAC‐alane adduct can activate Al−H and C−H bonds. The Al−H activation reaction presumably proceeds via cAAC‐alane adduct formation similar to that of analogous cAAC‐borane adducts. Additionally, Radius et al. have also reported insertions of carbene into Al−H bonds by cAACs and NHC‐alane compounds.^[319]^ Recently, Bekker et al. demonstrated that the coinage metal surfaces(Cu(111), Ag(111), and Au(111)) can be activated by cAACs.^[320]^ Since, cAACs constitute electron‐ rich ligand system, they can coordinate to the metal surface rendering it active. The metal surfaces are cleaned under a high vacuum before reacting with cAACs. The active metal surfaces can be used for catalytic applications.

## 4 Conclusion and Outlook

Since their discovery in 2005 by Bertrand and co‐workers, cAACs have been extensively explored for their unusual properties and applications. Activation of small molecules and enthalpically strong bonds, stabilization of transient paramagnetic transition metal species, and main group elements in lower or even in zero oxidation state have been foremost applications of cAACs. This is primarily due to their better donation properties and lower HOMO‐LUMO gap. cAAC enabled isolation of short‐lived transition metal complexes facilitates a better understanding of known catalytic reactions. Moreover, cAAC complexes of copper, gold, mercury, and ruthenium have played a major role in catalytic transformations. Furthermore, chiral cAACs have been used in enantioselective catalysis.^[83]^ Recently, cAAC complexes^[210]^ of copper, silver, and gold have been tested on a panel of human cancer cell lines such as Leukemia (HL 60), human lung adenocarcinoma epithelial cells (A549), and breast edenocarcinoma cells (MCF‐7), These complexes have been found to be active on HL 60 and MCF‐7 cell lines with the IC_50_ (half maximal inhibitory concentration) value in the range of micromolar to 100 nanomolar. Surprisingly, these complexes were found to be more active than cisplatin.^[210]^ These results are indeed encouraging and will give impetus to cancer chemotherapy in upcoming years. Recently, the synthesis of coinage metal complexes and the study of their photophysical properties has demonstrated that cAAC coinage metal complexes can be utilized in the fabrication of OLEDs.^[321]^ Notably, an interesting Cu(I) complex, [Cu(cAAC)_2_]PF_6_ has been synthesized that is, reportedly, the fastest copper(I) based triplet state emitter characterized to date. Due to inherent better electronic properties, cAACs have been instrumental in stabilizing several radical species which were otherwise highly challenging to stabilize. The discovery of BicAACs^[11]^ and cAACs as they are equipped with an even better donation and electronic properties than cAAC‐5, has given hope for the discovery of even more useful applications of cAACs in the future.

## Conflict of interest

The authors declare no conflict of interest.

## Biographical Information

*Saroj Kumar Kushvaha completed his B.Sc. from the University of Lucknow in 2010 and M.Sc. from DAVV Indore in 2012. Subsequently, he worked as a chemistry lecturer from 2013–2016. After working as a junior researcher at Defence research and development organization (DRDO) for a short period of time, he started working for his Ph.D. at the Indian Institute of Technology (IIT) Madras, Chennai, in 2017. Currently, he is working on cyclic (alkyl) (amino) carbenes and lanthanide‐based single‐molecule magnets*.

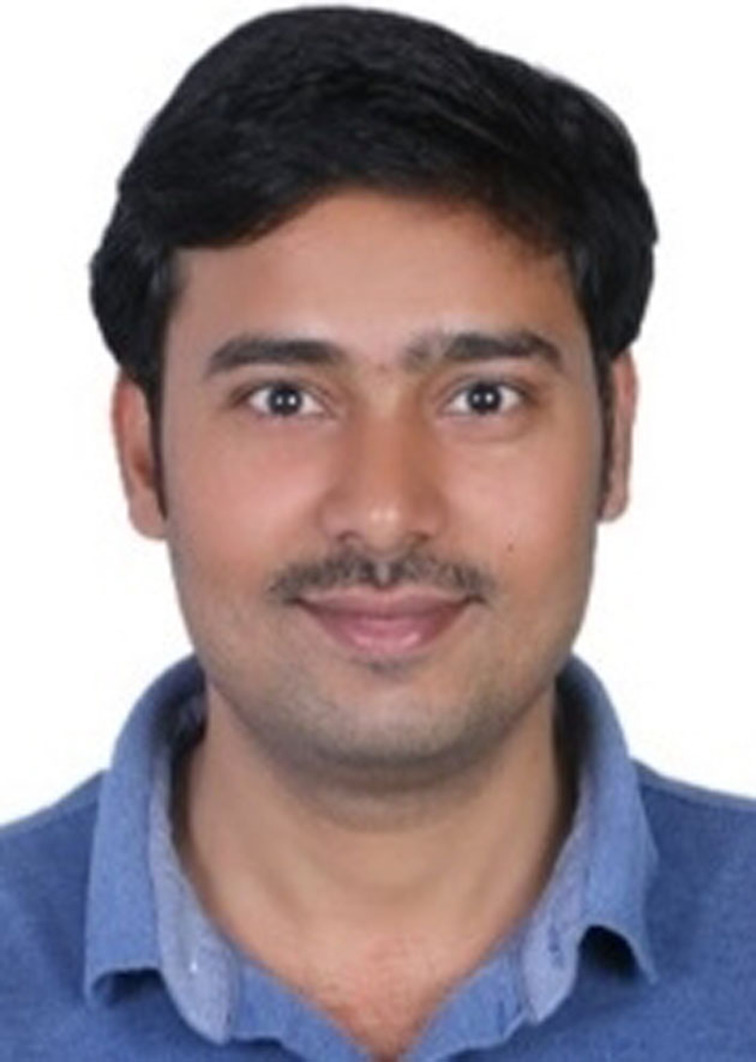

## Biographical Information

*Ankush Mishra completed his B.Sc. from Veer Bahadur Singh Purvanchal University, Jaunpur, in 2011 and M.Sc. from Allahabad University in 2013. He obtained his Ph.D. from Indian Institute of Technology (BHU), Varanasi under the supervision of Prof. Vandana Srivastava in 2019. Currently, he is working as Postdoc fellow in the group of Dr. Kartik Chandra Mondal, at Indian Institute of Technology Madras, Chennai, where he is working on organic transformations catalysed by cAAC stabilized metal complexes. He has published 13 research articles in reputed international journals*.

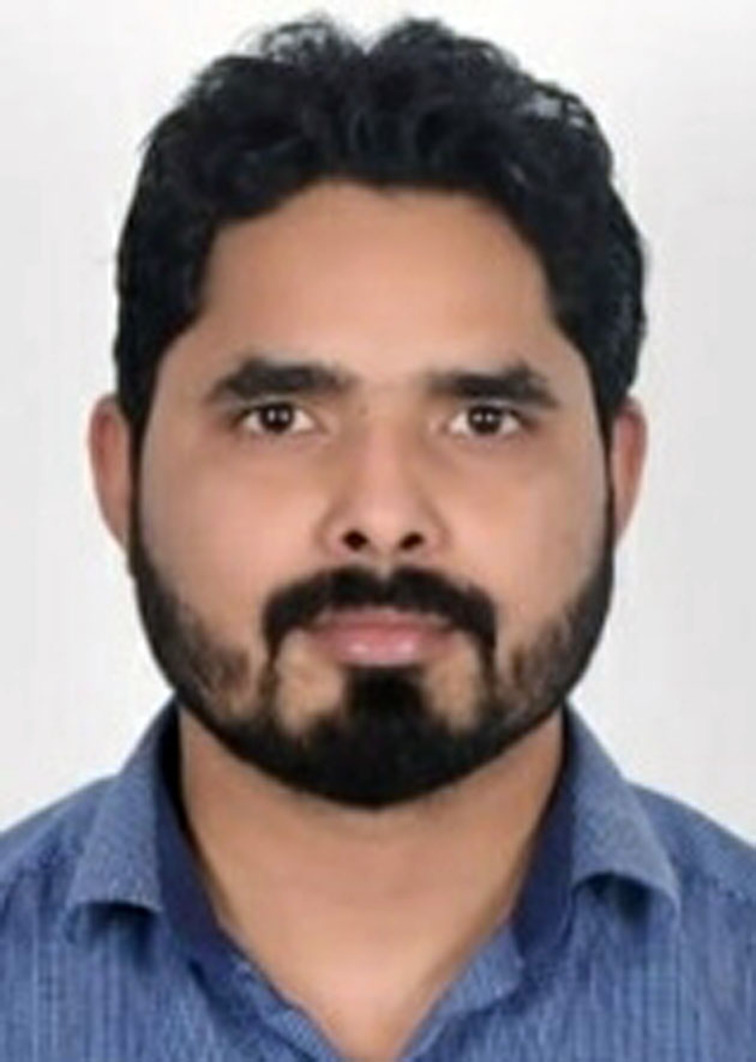

## Biographical Information

*Herbert W. Roesky received his doctorate from University of Göttingen. After working at Du Pont in the United States, he returned to Göttingen and finished his habilitation. In 1971, he became a professor at the Johann‐Wolfgang‐Goethe‐Universität, Frankfurt am Main. He moved to the University of Göttingen in 1980 and was the director of the Institute for Inorganic Chemistry until 2004. He is primarily known for his pioneering work on fluorides of both transition and main group elements. Currently, he is working on different aspects of cyclic (alkyl) (amino) carbenes. More than 1350 peer‐reviewed papers, articles, patents, and books record his research activities in the areas of Inorganic Chemistry and Material sciences*.

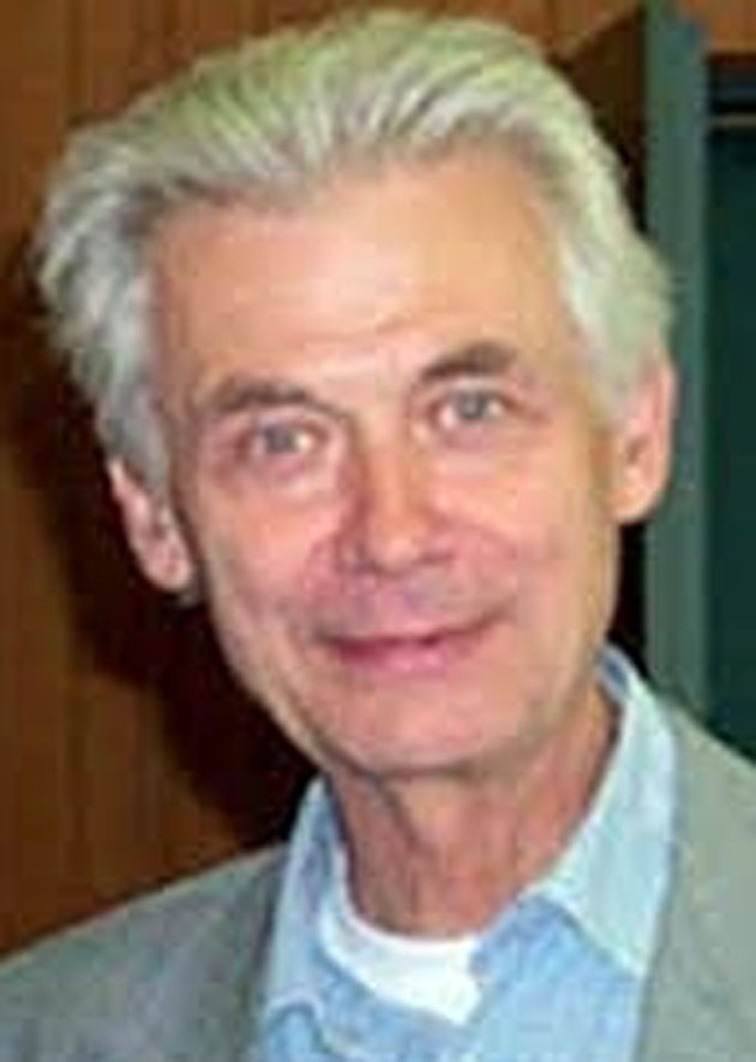

## Biographical Information

*Kartik Chandra Mondal obtained his Ph.D. from Karlsruhe Institute of Technology (KIT) under the supervision of Professor Annie K. Powell in 2011. He worked on 3d–4f molecular magnets. After a short stay as a postdoctoral researcher in the same group, he joined Professor Herbert W. Roesky as a postdoctoral researcher at the University of Göttingen (2011–2015). In 2016, he was appointed as an Assistant professor at the Indian Institute of Technology (IIT) Madras where he is working on cyclic (alkyl) (amino) carbenes. He has published more than 70 research articles in peer‐reviewed journals*.